# Supplementary material for: The effect of rehabilitation interventions on physical function and immobility-related complications in severe stroke: a systematic review
Source: BMJ Open. 2020 Feb 5;10(2):e033642. doi: 10.1136/bmjopen-2019-033642 (PMC7045156; doi:10.1136/bmjopen-2019-033642)
Supplement: Supplementary data [file bmjopen-2019-033642supp002.pdf]

Supplementary File 2

Supplementary Material- Medline Search Strategy

1. exp Stroke/
2. severe stroke.mp.
3. stroke severit*.mp.
4. stroke disabilit*.mp.
5. exp Physical Therapy Modalities/
6. exp Occupational Therapy/
7. exp Nursing Care/
8. physical rehabilitation.mp.
9. exp Stroke Rehabilitation/
10. exp Patient Positioning/
11. exp Posture/
12. exp Exercise/
13. exp Exercise Therapy/
14. passive exercise.mp.
15. exp "Range of Motion, Articular"/
16. manual technique.mp.
17. active exercise.mp.
18. Resistance Training/
19. exp Muscle Stretching Exercises/
20. exp Electric Stimulation/
21. exp Electric Stimulation Therapy/
22. exp Wheelchairs/
23. seat?.mp.
24. exp "Equipment and Supplies"/
25. exp Teaching/
26. exp Education/
27. exp Motor Skills/
28. exp Movement/
29. motor function.mp.
30. motor recovery.mp.
31. exp "Recovery of Function"/
32. exp "Activities of Daily Living"/
33. functional independence.mp.
34. physical independence.mp.
35. complicatio*.mp.
36. exp Pain/
37. exp Contracture/
38. exp Pressure Ulcer/
39. exp Respiratory Tract Infections/
40. exp Urinary Tract Infections
41. Muscle Spasticity/
42. Venous Thrombosis/

44. exp Pulmonary Embolism/

44. exp Accidental Falls/
45. exp Fatigue/
46. exp Depression/
47. 1 or 2 or 3 or 4

48. 5 or 6 or 7 or 8 or 9 or 10 or 11 or 12 or 13 or 14 or 15 or 16 or 17 or 18 or 19 or 20 or 21 or 22 or 23 or 24 or 25 or 26
49. 27 or 28 or 29 or 30
50. 31 or 32 or 33 or 34
51. 35 or 36 or 37 or 38 or 39 or 40 or 41 or 42 or 43 or 44 or 45 or 46
52. 47 and 48 and 49
53. 47 and 48 and 50
54. 47 and 48 and 51
55. limit 52 to ("all adult (19 plus years)" and randomized controlled trial)
56. limit 53 to ("all adult (19 plus years)" and randomized controlled trial)
57. limit 54 to ("all adult (19 plus years)" and randomized controlled trial)

Supplementary Table 1- Studies conducted in the acute – early subacute (<3 months) phase post-stroke

Study	Intervention Description	Intervention Duration	Intervention Delivered By	Stroke Severity Measure	Sample Size and Characteristics	Main Outcome Measures	Main Results	Quality of Evidence
AVERT trial collaboration group 2015 [1]	Very early mobilisation vs Usual care	Up to 14 days	PT and nursing staff	NIHSS	Very early mobilisation group NIHSS >16 (n=147)<br><br>Usual care group NIHSS >16 (n=144)	Favourable outcome (mRS 0-2) and mortality at 3 months	No difference in favourable outcome or mortality between groups	High
Bagley et al. 2005 [2]	Oswestry standing frame + standard physiotherapy vs Standard physiotherapy	14 daily sessions	PTs	BI^	Oswestry group (n=71) Median BI 1 (IQR 0-3)<br><br>Control group (n=69) Median BI 2 (IQR 1-3)	RMI, BI, HADS, NEADL, RMA, MAS (balance, sit to stand sections), TCT, CSI, GHQ-28	No differences between groups for all outcome measures. No differences in number of treatment sessions between groups or number of staff members required to treat each patient.	Low
Bradley et al. 1998 [3]	EMG biofeedback + conventional physiotherapy vs Placebo EMG + conventional physiotherapy	6 weeks	PTs	RMI	EMG group RMI ≤3 (n=7)<br><br>Conventional PT group RMI ≤3 (n=6)	MBS, mAS, 10MWT, RMI, sensation, proprioception NEADL	No differences between groups for MBS, RMI, NEADL and 10MWT. No improvements in mAS, sensation and proprioception for both groups.	Very low
Chang et al. 2012 [4]	Robot-assisted BWS treadmill gait training + conventional physiotherapy vs Conventional physiotherapy	2 weeks	PTs	FAC<br>LL FMA	Robot-assisted group (n=20) Mean FAC 0.5 (SD 0.5) Mean LL FMA 17.2 (SD 5.5)<br><br>Conventional group (n=17) Mean FAC 0.4 (SD 0.5) Mean LL FMA 16.8 (SD 5.7)	FAC, LL MI, LL FMA, Peak VO_2	Improvements in LL FMA and peak VO_2 in robot-assisted gait training group. No improvements in LL MI and FAC for both groups.	Low

Chen et al. 2011 [5]	Thermal stimulation + standard rehabilitation vs Standard rehabilitation	6 weeks	Thermal stimulation- PTs<br><br>Standard rehabilitation- PTs and OTs	FAC<br>LL FMA	Thermal stimulation group (n=17)<br>Median FAC 0 (IQR 0-1)<br>Median LL FMA 7 (4-11.5)<br><br>Standard rehab group (n=16)<br>Median FAC 0 (IQR 0-1)<br>Median LL FMA 6 (4.3-12.0)	LL FMA, LL MRC, mAS, mMAS, PASS (trunk control items), BBS, FAC	Thermal stimulation group demonstrated greater recovery gains compared to standard rehabilitation group in all outcomes except PASS. No difference between groups in MAS.	Low
Di Lauro et al. 2003 [6]	Intensive rehabilitative treatment vs Ordinary rehabilitative treatment	14 days	Therapists and nursing staff	BI^	Intensive rehab group (n=29)<br>Mean BI 1.4 (SD 1.4)<br><br>Ordinary rehab group (n=31)<br>Mean BI 1.5 (SD 1.5)	BI, mNIHSS	No differences between groups in BI or mNIHSS	Very low
Fong et al. 2013 [7]	Cueing wristwatch + conventional rehabilitation vs Sham wristwatch + conventional rehabilitation	3 weeks	Wristwatch- OTs<br><br>Conventional rehab- OT, PT, ST	Motor FIM	Cueing wristwatch group (n=19) Mean motor FIM 25.6 (SD 8.3)<br><br>Sham wristwatch group (n=16)<br>Mean motor FIM 28.2 (SD 10.0)	UL FMA, FTHUE, motor FIM, total number of UL movements	No differences between groups for UL FMA, FTHUE and motor FIM. More total UL movements in cueing wristwatch group but not significantly different between groups.	Low
Franceschini et al. 2009 [8]	BWS treadmill gait training + conventional treatment vs Conventional treatment	4 weeks	PTs	BI^	Treadmill training group (n=52)<br>Median BI 6 (IQR 3-9)<br>Median FAC 0 (IQR 0-0)<br><br>Conventional group (n=45)<br>Median BI 5 (IQR 3-7)<br>Median FAC 0 (IQR 0-0)	MI, TCT, mRS, BI, FAC, AS, LL proprioception, 6MWT, 10MWT, BS, WHS	No differences between groups. All patients were able to walk at discharge.	Low

Katz-Leurer et al. 2003 [9]	Leg cycle ergometer + regular therapy<br>vs<br>Regular therapy	8 weeks	Leg cycle ergometer- PTs<br><br>Regular therapy- PT, OT, ST	SSS	Leg cycle ergometer and regular rehabilitation groups- actual number of patients with severe stroke (SSS <30) not reported	FAI	No differences in decline in FAI between groups	Low
Liang et al. 2012 [20]	Thermal stimulation + standard rehabilitation<br>vs<br>Standard rehabilitation	6 weeks	Thermal stimulation- PTs<br><br>Standard rehabilitation- PTs and OTs	BI*	Thermal stimulation group (n=15)<br>Mean BI 30.3 (SD 11.1)<br><br>Standard rehab group (n=15)<br>Mean BI 27.7 (SD 14.3)	LL FMA, LL MRC, FAC, BBS, mMAS, BI	Improvements in LL FMA, LL MRC, FAC and mMAS in thermal stimulation group post-intervention and at 3-month follow-up. Improvements in BBS and BI in thermal stimulation group only at 3-month follow-up. Except for LL-FMA, all improvements disappeared at 6-month and 12-month follow-up.	Low
Lincoln et al. 1999 [11]	Standard physiotherapy + additional qualified PT therapy<br>vs<br>Standard physiotherapy + additional PTA therapy<br>vs<br>Standard physiotherapy	5 weeks	PTs/ PTAs	BI^	Qualified PT group (n=94)<br>Median BI 6 (IQR 3-9)<br><br>PTA group (n=93)<br>Median BI 6 (IQR 4-8)<br><br>Standard PT group (n=95)<br>Median BI 7 (IQR 3-9)	RMA- arm scale, ARAT, THPT, grip strength, mAS, BI, MCA	No differences between the groups across all outcomes	Low
Min et al. 2008 [12]	Acupuncture + systemic functional exercise<br>vs<br>Systemic functional exercise	? 3 months	Not reported	BI*	Acupuncture group (n=30)<br>Mean BI 27.28 (SD 5.41)<br><br>Systemic exercise group (n=30)<br>Mean BI 28.01 (SD 4.48)	FMA, BI	Acupuncture group demonstrated greater improvements in FMA and BI compared to the systemic exercise group	Very low

Ochi et al. 2015 [13]	Robot-assisted treadmill gait training + standard physiotherapy vs Conventional overground gait training + standard physiotherapy	4 weeks	Robot-assisted gait training- not reported Conventional gait training- PTs	FIM mobility FAC	Robot-assisted group (n=13) Median FAC 0 (IQR 0-1) Median FIM mobility 7 (IQR 6-10)<br><br>Conventional group (n=13) Median FAC 1 (IQR 0-1) Median FIM mobility 7 (IQR 7-9)	FAC, FMA, LL muscle torque, 10MWT, FIM (mobility scores)	Robot-assisted gait training group demonstrated greater improvements in FAC and peak LL muscle torque compared to the conventional group	Low
Rosewilliam et al. 2012 [14]	Wrist and finger NMES + usual care vs Usual care	6 weeks	NMES- staff group not reported, patients and carers<br><br>Usual care- PTs	BI^	NMES group (n=31) Mean BI 4.4 (SD 3.9) Mean ARAT 0.0 (SD 0.0)<br><br>Usual care group (n=36) Mean BI 2.5 (SD 2.9) Mean ARAT 0.6 (SD 3.5)	ARAT, BI, wrist AROM, wrist strength, grip strength	No differences in ARAT, BI or wrist AROM between groups. Improvements in wrist extensor and grip strength in the NMES group post-intervention but not maintained at follow-up.	Moderate
Sanchez-Sanchez et al. 2014 [15]	Functionally targeted physiotherapy techniques + conventional physiotherapy vs Conventional physiotherapy	Not reported	PTs	BI*	Functional techniques group (n=5) Mean BI 13 (SD 10.95)<br><br>Conventional therapy group (n=8) Mean BI 11.43 (SD 13.13)	BI	Functionally targeted physiotherapy group demonstrated greater improvement compared to the conventional physiotherapy group when using functional principal component analysis	Very low
Tang et al. 2014 [16]	Contemporary Bobath approach with early sitting, standing and walking vs Contemporary Bobath approach	8 weeks	PTs	STREAM BBS	Early contemporary group (n=24) Mean STREAM 1.4 (SD 1.0) Mean BBS 0 (SD 0)<br><br>Contemporary group (n=24) Mean STREAM 1.3 (SD 0.9) Mean BBS 0 (SD 0)	STREAM, BBS	Improvements in STREAM and BBS in the contemporary Bobath approach with early mobilisation group	Low

Supplementary Table 2- Studies conducted in the acute – late subacute (<6 months) phase post-stroke

Study	Intervention Description	Intervention Duration	Intervention Delivered By	Stroke Severity Measure	Sample Size and Characteristics	Main Outcome Measures	Main Results	Quality of Evidence
Bai et al. 2014 [17]	Staged physical rehabilitation interventions + routine care vs Routine care	6 months	PTs and OTs	BI*	Staged rehab group (n=83) Mean BI 28 (range 24-31)<br><br>Routine care group (n=82) Mean BI 23 (range 19-27)	BI, mAS	Staged rehab group demonstrated higher BI scores than the routine care group at 1, 3- and 6-months post-stroke. 42.9% of patients in the routine care group demonstrated spasticity in at least one body part compared to 36.4% of patients in the staged rehab group.	Low
Calabrò et al. 2015 [18]	Robotic verticalisation + standard physiotherapy vs Physiotherapy-assisted verticalisation + standard physiotherapy	6 weeks	PTs	PASS<br>LL FMA	Robotic group (n=10) Mean PASS 3 (SD 1) Mean LL FMA 13 (SD 3)<br><br>Physiotherapy group (n=10) Mean PASS 3 (SD 3) Mean LL FMA 12 (SD 6)	PASS, LL FMA, MRC, vertical posture tolerance	Both interventions were well tolerated. Robotic group demonstrated greater improvements in MRC, LL FMA and PASS compared to the physiotherapy group	Very low
Chaiyawat and Kulkantrakorn 2012 [19,20]	Home based physiotherapy programme vs Usual care	6 months	PTs	BI*	Home PT group (n=30) Mean BI 31.7 (SD 5.9) Mean NIHSS 16.4 (SD 4.1)<br><br>Usual care group (n=30) Mean BI 33.2 (SD 4.8) Mean NIHSS 17.8 (SD 3.9)	BI, HADS, mRS, EQ-5D	Home therapy group demonstrated greater improvements in BI, HADS, mRS and EQ-5D compared to the usual care group which were maintained at 2-year follow-up.	Very low
Jongbloed et al. 1989 [21]	Functional treatment approach vs Sensorimotor integrative treatment approach	8 weeks	OTs	BI*	Functional treatment group (n=13) Mean BI 31.5<br><br>Sensorimotor integrative treatment group (n=9) Mean BI 30	BI, meal preparation, eight subtests of Sensorimotor Integration Test Battery	No differences between groups on all outcome measures	Very low

Kwakkel et al. 1999 [22] 2002 [23] 2002 [24]	Additional UL training + usual care vs Additional LL training + usual care vs UL/LL pressure splint immobilisation + usual care	20 weeks	PTs and OTs	BI^	UL training group (n=33)<br>Median BI 5 (IQR 3-7)<br>LL training (n=31)<br>Median BI 6 (IQR 3-8)<br>Splint control group (n=37)<br>Median BI 5.5 (IQR 3-7)	BI, FAC, ARAT, 10MWT, SIP, NHP, FAI	LL training group had significantly higher BI, FAC, walking speed and ARAT than splint control group post-intervention. UL training group had significantly higher ARAT than splint control group post-intervention. No significant differences in all outcomes were seen between groups from 6 months onwards up until 12-month follow-up.	Moderate
					CRP sub-study<br>UL training group (n=18)<br>Mean BI 5.0 (SD 2.0)<br>LL training (n=17)<br>Mean BI 6.3 (SD 2.7)<br>Splint control group (n=18)<br>Mean BI 5.3 (SD 2.7)	10MWT, mean CRP of arm/leg movements	LL training group had significantly higher comfortable walking speed than UL and splint control groups post-intervention. No differences were seen for the mean CRP of arm/leg movements between groups.	
Morone et al. 2011 [25] 2012 [26]	Robot-assisted BWS treadmill gait training + standard physiotherapy vs Conventional gait training + standard physiotherapy	3 months	PTs	BI*	Robotic groups<br>Low motricity (n=12)<br>Mean BI 14.2 (SD 11.8)<br>High motricity (n=12)<br>Mean BI 20.0 (SD 17.2)<br><br>Conventional groups<br>Low motricity (n=12)<br>Mean BI 7.9 (SD 8.9)<br>High motricity (n=12)<br>Mean BI 24.6 (SD 15.3)	FAC, LL AS, RMI, MI, TCT, CNS, BI, RS, 6MWT, 10MWT	Higher FAC in low motricity robotic training group compared to low motricity conventional training group post-intervention. At discharge, higher RMI, BI, TCT, RS and 6MWT in low motricity robotic training group compared to low motricity conventional training group. No differences were seen between the higher motricity groups post-intervention or on discharge.<br>At 12-month follow-up, low motricity robotic training group had higher FAC, BI and RMI compared to low motricity conventional training group. No differences were seen between the higher motricity groups.	Very low

Yang et al. 2014 [27]	Acupuncture + rehabilitation training vs Rehabilitation training	8 weeks	Acupuncture- not reported<br><br>Rehabilitation- PTs	NIHSS BI*	Acupuncture group (n=33)<br>Mean NIHSS 25.5 (SD 2.4)<br>Mean BI 39.4 (SD 3.9)<br><br>Rehabilitation group (n=31)<br>Mean NIHSS 24.1 (SD 3.1)<br>Mean BI 38.1 (SD 4.3)	NIHSS, FMA, BI	Acupuncture group demonstrated higher scores on all outcome measures compared to the rehabilitation group	Very low
Yue et al. 2012 [28]	Acupressure treatment + routine care vs Routine care	3 months	Nurses	BI*	Acupressure group (n=35)<br>Mean BI 26.8 (SD 15.2)<br><br>Routine care group (n=34)<br>Mean BI 24.4 (SD 16.8)	FMA, BI	Acupressure group demonstrated greater improvements in BI and FMA only at 3-month time frame	Very low

Supplementary Table 3- Studies conducted in the chronic (>6 months) phase post-stroke

Study	Intervention Description	Intervention Duration	Intervention Delivered By	Stroke Severity Measure	Sample Size and Characteristics	Main Outcome Measures	Main Results	Quality of Evidence
Rodrigues et al. 2017 [29]	Robot-assisted BWS treadmill gait training with progressively increased speeds<br>vs<br>Robot-assisted bodyweight supported treadmill gait training with progressively decreased speeds	6 weeks	Not reported	LL FMA<br>FAC	Faster speed group (n=10)<br>Median FAC 1.5 (1–2)<br>Mean LL FMA 19.5 (SD 4.6)<br><br>Slower speed group (n=10)<br>Median FAC 1 (1–2)<br>Mean LL FMA 17.5 (SD 2.8)	FAC, TUG, 6MWT, 10MWT, BBS, LL FMA	Improvements in FAC, FMA, TUG and 6MWT in the slower speed group compared to the faster speed group.	Very low
Sackley et al. 2015 [30]	OT intervention<br>vs<br>Usual care	3 months	OTs	BI^	OT intervention group-<br>BI 0-4 n=268<br>BI 5-9 n=129<br><br>Usual care group-<br>BI 0-4 n=234<br>BI 5-9 n=104	BI, RMI, GDS, EQ-5D-3L	No differences between the groups on any outcome measure at 3-, 6- and 12-months post-randomisation. Higher fall rate per resident in OT intervention group at 3 months.	High
Volpe et al. 2008 [31]	Intensive standard UL therapy<br>vs<br>Intensive robot-assisted UL therapy	6 weeks	Therapists	NIHSS	Therapist group (n=10)<br>Mean NIHSS 17 (SD 1)<br><br>Robot group (n=11)<br>Mean NIHSS 17 (SD 1)	FMA- UL, MRC-shoulder/ elbow, mAS, UL PROM, SIS, ARAT, BDS, shoulder dislocation, pain	No difference between groups in shoulder and elbow strength and motor function. No improvements in other outcome measures for both groups.	Very low
Zhang and Li 2014 [32]	Trunk acupuncture + rehabilitation training<br>vs<br>Rehabilitation training alone	16 weeks	Not reported	BI*	Acupuncture group (n=30)<br>Mean BI 22.50 (SD 6.79)<br><br>Rehabilitation group (n=29)<br>Mean BI 24.48 (SD 7.23)	BI, BBS	Acupuncture group demonstrated higher scores on BI and BBS compared to the rehabilitation group.	Very low

ARAT- Action Research Arm Test, AROM- active range of movement, AS- Ashworth Scale, BBS- Berg Balance Scale, BDS- Becks Depression Scale, BI*- Barthel Index (original version scored out of 100), BI^ - Barthel Index(revised version score out of 20), BS- Borg Scale, BWS- bodyweight supported, CNS- Canadian Neurological Scale, CRP- continuous relative phase, CSI- Caregiver Strain Index, EQ-5D-3L- EuroQoL questionnaire, FAC- Functional Ambulation Category, FAI- Frenchay Activities Index, FIM- Functional Independence Measure, FMA- Fugl-Meyer Assessment, FTHUE- Functional Test for the Hemiplegic Upper Extremity, GDS- Geriatric Depression Scale, GHQ-28- General Health Questionnaire-28, HADS- Hospital Anxiety and Depression Scale, LL- lower limb, MAS- Motor Assessment Scale, mAS- Modified Ashworth Scale, MCA- Motor Club Assessment, MI- Motricity Index, mMAS- Modified Motor Assessment Scale, MMSE- Mini-Mental State Examination, mNIHSS- Modified National Institutes of Health Stroke Scale, mRS- Modified Rankin Scale, MRC- Medical Research Council Scale for Muscle Strength, NEADL- Nottingham Extended Activities of Daily Living, NHP- Nottingham Health Profile, NIHSS- National Institutes of Health Stroke Scale, OT- occupational therapist, PASS- Postural Assessment Scale for Stroke Patients, PROM- passive range of movement, PT- physiotherapist, PTA- physiotherapy assistant, RMA- Rivermead Motor Assessment, RMI- Rivermead Mobility Index, RS- Rankin Scale, SIP- Stroke Impact Profile, SIS- Stroke Impact Scale, ST- speech therapist, STREAM- Stroke Rehabilitation Assessment of Movement, TCT- Trunk Control Test, THPT- Ten-Hole Peg Test, TUG- Timed Up and Go, UL- upper limb, WHS- Walking Handicap Scale, 6MWT- 6 minute walk test, 10MWT- 10 metre walk test

Supplementary Table 4- Overview of Measures of Physical Function and Immobility-Related Complications

Body Function	Activity	Participation	Complications
**Cardiorespiratory Function**	**Activities of Daily Living**	**Instrumental Activities of Daily Living**	**Adverse Effects**
Aerobic capacity	Barthel Index	Frenchay Activities Index	Pain
Borg scale	Functional Independence Measure- motor	Nottingham Extended ADL Scale	Shoulder dislocation
Cardiovascular response	Functional Independence Measure- total	Meal preparation	
Ventilatory response	Modified Rankin Scale		
			**Caregiver Burden**
		**Perceived Health Status**	Caregiver Strain Index
**Neurological Impairment**	**Balance and Postural Control**	Stroke Impact Scale	
Canadian Neurological Scale	Berg Balance Scale	General Health Questionnaire-28	
National Institutes of Health Stroke Scale	Postural Assessment Scale for Stroke		**Depression**
	Trunk Control Test		Beck Depression Scale
	Vertical Posture Test	**Quality of Life**	Geriatric Depression Scale
**Sensorimotor Function**		EQ-5D	Hospital and Depression Scale
Active range of movement- UL		Nottingham Health Profile	
Grip strength	**Gait**	Sickness Impact Profile	
Fugl Meyer- UL	Continuous relative phase between UL/LL movement		**Mortality**
Fugl Meyer- LL	Comfortable/maximal walking speed		Mortality
Fugl Meyer- UL and LL	Functional Ambulation Category		
Motricity Index	Number of independent walkers		
Medical Research Council strength- UL	Time taken to walk 50 metres independently		**Spasticity**
Medical Research Council strength- LL	Walking Handicap Scale		Modified Ashworth Scale
Medical Research Council strength- UL and LL	6 minutes walking test		
Number of upper limb movements	10 metre walking test		
Sensation/proprioception			
Sensorimotor integration test			
	**General Physical Activity**		
	Modified Bobath Scale		
	Motor Assessment Scale		
	Rivermead Motor Assessment		
	Rivermead Mobility Index		
	Stroke Rehabilitation Assessment of Movement		
	Timed Up and Go		
	**Upper Limb Function**		
	Action Research Arm Test		
	Functional Test for Hemiplegic Upper Extremity		
	9 Hole Peg Test		
	10 Hole Peg Test		

Supplementary Results- Outcomes Supported by Low or Very Low-Quality Evidence

Body function

Cardiorespiratory Function

Two studies explored participants' cardiorespiratory response to different types of treadmill gait training within the acute to early subacute phase post-stroke.[4,8] There was low-quality evidence that 2 weeks of robot-assisted bodyweight supported treadmill gait training delivered in the first 6 weeks post-stroke improved peak VO_2 compared to conventional gait training. [4] There was low-quality evidence that a 4-week course of bodyweight supported treadmill training delivered in the first 3 months post-stroke was not perceived to be more effortful than conventional gait training.[8]

Neurological Impairment

Three studies evaluated changes in neurological function. [6,25,27] In the acute to early subacute phase post-stroke, there was very low-quality evidence that there was no difference in an intensive or ordinary 2-week acute physical rehabilitation programme on reducing neurological impairment at 2 weeks and 6 months post-stroke.[6] In the acute to late subacute phase post-stroke, there was very low-quality evidence that a 3-month course of robot-assisted bodyweight supported treadmill gait training commenced within the first 6 weeks post-stroke was just as effective as conventional gait training on improving neurological function.[25] There was very low-quality evidence that an 8-week course of acupuncture provided in conjunction with rehabilitation during the subacute phase of stroke reduced neurological impairment compared to rehabilitation alone.[27]

Sensorimotor Function

Sixteen studies evaluated changes in sensorimotor function. Nine studies were performed in the acute to early subacute phase post-stroke, [3-5,7,8,10-13] five studies in the acute to late subacute phase post-stroke,[18,21,25,27,28] and two studies in the chronic phase post-stroke.[29,31] In the acute to early subacute phase post-stroke, there was low quality evidence from two studies that thermal stimulation in conjunction with standard rehabilitation resulted in improvements in lower limb sensorimotor function and strength when compared to standard rehabilitation alone.[5,10] Improvements in lower limb sensorimotor function were maintained at 12 months post-intervention. There was low quality evidence that 2 weeks of robot-assisted bodyweight supported treadmill gait training resulted in improvements in lower limb sensorimotor function but not strength compared to conventional gait training.[4] There was low quality evidence that there was no difference between: 4 weeks of robot-assisted treadmill gait training and conventional gait training on improving lower limb sensorimotor function;[13] wearing a cueing wristwatch and wearing a sham wristwatch for 3 hours per weekday for 3 weeks during rehabilitation on improving upper limb sensorimotor function and number of arm movements;[7] a 4-week course of bodyweight supported treadmill training and conventional overground gait training on improving lower limb strength;[8] and a 5-week course of additional upper limb therapy provided by a qualified physiotherapist or a physiotherapy assistant and standard physiotherapy on improving upper limb motor activity and grip strength.[11]

There was very low-quality evidence that a thrice weekly, 6-week course of electromyography (EMG) biofeedback combined with conventional physiotherapy had no effect on improving lower limb active range of movement when compared to conventional

physiotherapy alone.[3] There was very low-quality evidence that a 3-month course of acupuncture in conjunction with rehabilitation resulted in better upper and lower limb sensorimotor function when compared to rehabilitation alone.[12]
In the acute to late subacute phase post-stroke, there was very low quality evidence that a 6-week course of robotic tilt-table verticalisation that combines cyclic leg movements and FES and used in conjunction with standard physiotherapy resulted in better lower limb strength and sensorimotor function compared to physiotherapy-assisted verticalisation using a standard tilt-table and used in conjunction with standard physiotherapy.[18] There was very low-quality evidence that an 8-week course of acupuncture provided in conjunction with rehabilitation resulted in improvements in upper and lower limb sensorimotor function compared to rehabilitation alone.[27] There was very low-quality evidence that a 3-month course of nurse-led acupressure resulted in improvements in upper and lower limb motor function compared to routine care.[28] There was very low quality evidence that there was no difference between: a functionally-orientated and a sensorimotor integrative occupational therapy treatment approach delivered over 8 weeks on improving upper limb sensorimotor function;[21] and a 3-month course of robot-assisted bodyweight supported treadmill gait training and conventional gait training on improving lower limb power.[25]
In the chronic phase post-stroke, there was very low-quality evidence that a 6-week course of robot-assisted bodyweight supported treadmill gait training using slower treadmill speeds resulted in improvements in lower limb sensorimotor function compared to similar treadmill training using faster treadmill speeds.[29] There was very low-quality evidence that either an intensive therapist-driven UL protocol or an intensive robotic-driven UL protocol delivered thrice weekly for 6 weeks resulted in an improvement in shoulder and elbow sensorimotor function.[31]

Activity

Activities of Daily Living

Sixteen studies explored independence and ability to perform activities of daily living (ADLs). Nine studies were completed in the acute to early subacute phase,[2,6-8,10-13,15] six studies were completed in acute to late subacute phase[17,19-21,25,27,28] and one study was completed in the chronic phase.[32]
In the acute to early subacute phase, there was low quality evidence that a 6-week course of thermal stimulation used in conjunction with standard rehabilitation resulted in improvements in ADL independence 3 months post-stroke compared to standard rehabilitation alone, although improvements were not seen at 6 months post-stroke.[10]
There was low quality evidence that there was no difference between: regular physiotherapy and regular physiotherapy in conjunction with use of an Oswestry standing frame delivered over 14 consecutive weekdays in the first 3 months post-stroke on ADL independence;[2] wearing a cueing wristwatch and wearing a sham wristwatch for 3 hours per weekday for 3 weeks during rehabilitation on ADL independence;[7] a 4-week course of bodyweight supported treadmill training and conventional overground gait training on improving ADL independence;[8] a 5-week course of additional upper limb therapy provided by a qualified physiotherapist or a physiotherapy assistant and standard physiotherapy on improving ADL independence;[11] and 4 weeks of robot-assisted treadmill gait and conventional overground gait training on ADL independence.[13]
There was very low-quality evidence that there was no difference in an intensive or ordinary 2-week acute physical rehabilitation programme in improving ADL independence at 2 weeks

and 6 months post-stroke.[6] There was very low-quality evidence that a 3-month course of acupuncture in conjunction with rehabilitation resulted in better ADL independence when compared to rehabilitation alone.[12] There was very low-quality evidence that providing additional physiotherapy in conjunction to regular rehabilitation in the first few weeks post-stroke resulted in improvements in ADL independence at 6 months post-stroke compared to regular rehabilitation alone.[15]

In the acute to late subacute phase, there was low quality evidence that a 6-month course of a staged physical rehabilitation programme resulted in greater improvements in ADL independence compared to usual care that did not involve formal rehabilitation.[17] There was very low-quality evidence that a monthly home-based physiotherapy programme delivered over 6 months resulted in improvements in ADL independence compared to standard care.[19,20] There was very low-quality evidence that there was no difference between a functionally orientated or a sensorimotor integrative occupational therapy treatment approach delivered over 8 weeks on ADL independence.[21] There was very low-quality evidence that a 3-month course of robot-assisted bodyweight supported treadmill gait training resulted in improvements in ADL independence compared to conventional gait training.[25] Improvements were only seen in the cohort of participants who demonstrated significant motor impairment. Improvements were maintained at the 2-year follow-up.[26] There was very low-quality evidence that an 8-week course of acupuncture provided in conjunction with rehabilitation during the subacute phase of stroke improved ADL independence compared to rehabilitation alone.[27] There was very low-quality evidence that a 3-month course of nurse-led acupressure resulted in improvements in ADL independence compared to routine care.[28]

In the chronic phase, there was very low-quality evidence that a 16-week course of trunk acupuncture combined with rehabilitation training resulted in greater improvements in ADL independence compared to rehabilitation training alone.[32]

Balance and Postural Control

Eight studies investigated balance and postural control. Four studies were completed in the acute to early subacute phase,[2,5,10,16] two studies were completed in the acute to late subacute phase[18,25] and two studies were completed in the chronic phase.[29,32]

In the acute to early subacute phase, there was low quality evidence that a 6-week course of thermal stimulation in conjunction with standard rehabilitation resulted in improvements in trunk postural control but not balance compared to standard rehabilitation alone.[5] In a separate study, there was low quality evidence that a 6-week course of thermal stimulation in conjunction with standard rehabilitation resulted in improvements in balance 3 months post-stroke compared to standard rehabilitation alone, although improvements were not seen at 6 months post-stroke.[10] There was low quality evidence that there was no difference between regular physiotherapy and regular physiotherapy in conjunction with use of an Oswestry standing frame delivered over 14 consecutive weekdays in the first 3 months post-stroke on trunk postural control.[2] There was low quality evidence that an 8-week course of physiotherapy involving early mobilisation combined with the Bobath approach resulted in improvements in balance when compared to physiotherapy just involving the Bobath approach.[16]

In the acute to late subacute phase, there was very low quality evidence that a 6-week course of robotic tilt-table verticalisation that combines cyclic leg movements and FES and used in conjunction with standard physiotherapy resulted in improved postural control

during different activities compared to physiotherapy-assisted verticalisation using a standard tilt-table and used in conjunction with standard physiotherapy.[18] There was very low-quality evidence that a 3-month course of robot-assisted bodyweight supported treadmill gait training resulted in improvements in trunk control compared to conventional gait training.[25] Improvements were only seen in the cohort of participants who demonstrated significant motor impairment.

In the chronic phase, there was very low-quality evidence that a 6-week course of robot-assisted bodyweight supported treadmill gait training resulted in improvements in balance regardless if slower or faster treadmill training speeds were used.[29] There was very low-quality evidence that a 16-week course of trunk acupuncture combined with rehabilitation training resulted in greater improvements in balance compared to rehabilitation training alone.[32]

Gait

Eight studies investigated gait, which included gait ability and gait speed. Six studies were performed in the acute to early subacute phase,[3-5,8,10,13] one study was performed in the acute to late subacute phase[25] and one study was performed in the chronic phase.[29] In the acute to early subacute phase, there was low quality evidence from two studies that a 6-week course of thermal stimulation in conjunction with standard rehabilitation resulted in improvements in gait ability compared to standard rehabilitation alone.[5,10] There was low quality evidence that 4 weeks of robot-assisted treadmill gait training resulted in better gait ability than conventional gait training.[13] There was low quality evidence that there was no difference between: a 2-week course of robot-assisted bodyweight supported treadmill gait training and conventional gait training delivered in the first 6 weeks post-stroke on improving gait ability;[4] a 4-week course of bodyweight supported treadmill training and conventional overground gait training on improving gait ability;[8] and a thrice weekly, 6-week course of EMG biofeedback combined with conventional physiotherapy and conventional physiotherapy alone in improving gait speed.[3] In the acute to late subacute phase, there was very low-quality evidence that a 3-month course of robot-assisted bodyweight supported treadmill gait training resulted in improvements in gait ability compared to conventional gait training.[25] Improvements were only seen in the cohort of participants who demonstrated significant motor impairment. Improvements were maintained at the 2-year follow-up.[26]

In the chronic phase, there was very low-quality evidence that a 6-week course of robot-assisted bodyweight supported treadmill gait training using slower treadmill speeds resulted in improvements gait ability compared to similar treadmill training using faster treadmill speeds.[29]

General Physical Activity

Seven studies examined the effects of different interventions on improving general physical activity. Six studies were performed in the acute to early subacute phase[2,3,5,10,11,16] and one study was performed in the acute to late subacute phase.[25] In the acute to early subacute phase, there was low quality evidence from two studies that thermal stimulation in conjunction with standard rehabilitation resulted in improvements in physical activity when compared to standard rehabilitation alone.[5,10] Improvements were seen up until 3 months post-intervention but disappeared at the 6-month follow-up. There was low quality evidence that an 8-week course of physiotherapy involving early mobilisation combined with the Bobath approach resulted in improvements in physical activity when compared to

physiotherapy just involving the Bobath approach.[16] There was low quality evidence that there was no difference between: regular physiotherapy and regular physiotherapy in conjunction with use of an Oswestry standing frame delivered over 14 consecutive weekdays in the first 3 months post-stroke on physical activity;[2] and a 5-week course of additional upper limb therapy provided by a qualified physiotherapist or a physiotherapy assistant and standard physiotherapy on improving physical activity.[11] There was very low-quality evidence that there was no difference between a thrice weekly, 6-week course EMG biofeedback combined with conventional physiotherapy and conventional physiotherapy alone on improving physical activity.[3]
In the acute to late subacute phase, there was very low-quality evidence that a 3-month course of robot-assisted bodyweight supported treadmill gait training resulted in improvements in physical activity compared to conventional gait training.[25] Improvements were only seen in the cohort of participants who demonstrated significant motor impairment. Improvements were maintained at the 2-year follow-up.[26]

Upper Limb Function
Two studies investigated changes in upper limb function.[11,31] In the acute to early subacute phase, there was low quality evidence that a 5-week course of additional upper limb therapy provided by a qualified physiotherapist was no more effective at improving upper limb function than additional upper limb therapy provided by a physiotherapy assistant or to standard physiotherapy.[11] In the chronic phase, there was very low-quality evidence that there was no improvement in upper limb function with either an intensive therapist-driven UL protocol or an intensive robotic-driven UL protocol delivered thrice weekly for 6 weeks.[31]

Participation
Instrumental Activities of Daily Living
Four studies investigated the effect of different interventions on instrumental ADLs.[2,3,9,21] In the acute to early subacute phase, there was low quality evidence that there was no difference between: regular physiotherapy and regular physiotherapy in conjunction with use of an Oswestry standing frame delivered over 14 consecutive weekdays on ability to perform instrumental ADLs at 6 months post-stroke,[2] and an 8-week course of rehabilitation with the addition of a leg cycling machine compared to regular rehabilitation alone on instrumental ADLs 6 months post stroke.[9] There was very low-quality evidence that there was no difference between a thrice weekly, 6-week course of electromyography (EMG) biofeedback combined with conventional physiotherapy and conventional physiotherapy alone in improving performance in instrumental ADLs.[3]
In the acute to late subacute phase, there was very low-quality evidence that there was no difference between a functionally orientated or a sensorimotor integrative occupational therapy treatment approach delivered over 8 weeks on the ability to prepare meals.[21]

Perceived Health Status
Two studies explored carers' and patients' perceived health status.[2,31] In the acute to early subacute phase, there was low quality evidence that there was no difference between regular physiotherapy and regular physiotherapy in conjunction with use of an Oswestry standing frame delivered over 14 consecutive weekdays on carer's perceived health status at 12 weeks and 6 months post-stroke.[2] In the chronic phase, there was very low-quality evidence that there was no change in patient's perceived health status with the provision of

either an intensive therapist-driven UL protocol or an intensive robotic-driven UL protocol delivered thrice weekly for 6 weeks.[31]

Quality of Life

There was very low-quality evidence that a monthly home-based physiotherapy programme delivered over 6 months resulted in an improvement in quality of life compared to standard care.[19]

Complications

Caregiver Burden

There was low quality evidence that there was no difference between regular physiotherapy and regular physiotherapy in conjunction with use of an Oswestry standing frame delivered over 14 consecutive weekdays in the first 3 months post-stroke on caregiver strain and psychological well-being at 12 weeks and 6 months post-stroke.[2]

Depression

Three studies explored changes in depression.[2,20,31] In the acute to early subacute phase, there was low quality evidence that there was no difference between regular physiotherapy and regular physiotherapy in conjunction with use of an Oswestry standing frame delivered over 14 consecutive weekdays on depression at 12 weeks and 6 months post-stroke.[2] In the acute to late subacute phase, there was very low-quality evidence that a monthly home-based physiotherapy programme delivered over 6 months resulted in a reduction in level of depression compared to standard care.[20] In the chronic phase, there was very low-quality evidence that there was no difference between an intensive therapist-driven UL protocol and an intensive robotic-driven UL protocol delivered thrice weekly for 6 weeks in reducing depression.[31]

Shoulder Pain/Dislocation

There was very low-quality evidence that either an intensive therapist-driven UL protocol or an intensive robotic-driven UL protocol delivered thrice weekly for 6 weeks had no effect on shoulder pain nor caused any shoulder dislocation when delivered to participants in the chronic phase post-stroke.[31]

Spasticity

Six studies explored the effect of different interventions on spasticity.[3,8,11,17,25,31] In the acute to early subacute phase, there was low quality evidence that there was no difference between: bodyweight supported treadmill training and conventional overground gait training delivered over 4 weeks on reducing lower limb spasticity;[8] and a 5-week course of additional upper limb therapy provided by a qualified physiotherapist or a physiotherapy assistant and standard physiotherapy on reducing upper limb spasticity.[11] There was very low-quality evidence that there was no reduction in spasticity with a 6-week course of conventional physiotherapy with or without EMG biofeedback.[3]

In the acute to late subacute phase, there was low quality evidence that a 6-month course of a staged physical rehabilitation programme resulted in a lower incidence of upper and lower limb spasticity compared to usual care that did not involve formal rehabilitation.[17]

There was very low-quality evidence that a 3-month course of either robot-assisted

bodyweight supported treadmill training or conventional gait training had no effect on reducing lower limb spasticity.[25]
In the chronic phase, there was very low-quality evidence that there was no difference between an intensive therapist-driven UL protocol and an intensive robotic-driven UL protocol delivered thrice weekly for 6 weeks in reducing UL spasticity.[31]

Supplemental References

1 AVERT Trial Collaboration group. Efficacy and safety of very early mobilisation within 24 h of stroke onset (AVERT): a randomised controlled trial. *Lancet* 2015;386:46-55.
2 Bagley P, Hudson M, Forster A, et al. A randomized trial evaluation of the Oswestry Standing Frame for patients after stroke. *Clin Rehabil* 2005;19:354-364.
3 Bradley L, Hart BB, Mandana S, et al. Electromyographic biofeedback for gait training after stroke. *Clin Rehabil* 1998;12:11-22.
4 Chang WH, Kim MS, Huh JP, et al. Effects of Robot-Assisted Gait Training on Cardiopulmonary Fitness in Subacute Stroke Patients: A Randomized Controlled Study. *Neurorehab Neural Re* 2012;26(4):318-324.
5 Chen JC, Lin CH, Wei YC, et al. Facilitation of motor and balance recovery by thermal intervention for the paretic lower limb of acute stroke: a single-blind randomized clinical trial. *Clin Rehabil* 2011;25(9):823-832.
6 Di Lauro A, Pellegrino L, Savastano G, et al. A randomized trial on the efficacy of intensive rehabilitation in the acute phase of ischemic stroke. *J Neurol* 2003;250:1206-1208.
7 Fong K, Yang N, Chan M, et al. Combined effects of sensory cueing and limb activation on unilateral neglect in subacute left hemiplegic stroke patients: a randomized controlled pilot study. *Clin Rehabil* 2013;27(7):628-637.
8 Franceschini M, Carda S, Agosti M, et al. Walking After Stroke: What Does Treadmill Training With Body Weight Support Add to Overground Gait Training in Patients Early After Stroke? A Single-Blind, Randomized, Controlled Trial. *Stroke* 2009;40:3079-3085.
9 Katz-Leurer M, Carmeli E and Shochina M. The effect of early aerobic training on independence six months post stroke. *Clin Rehabil* 2003;17:735-741.
10 Liang CC, Hsieh TC, Lin CH, et al. Effectiveness of Thermal Stimulation for the Moderately to Severely Paretic Leg After Stroke: Serial Changes at One-Year Follow-Up. *Arch Phys Med Rehab* 2012;93:1903-1910.
11 Lincoln NB, Parry RH and Vass CD. Randomized, Controlled Trial to Evaluate Increased Intensity of Physiotherapy Treatment of Arm Function After Stroke. *Stroke* 1999;30:573-579.
12 Min M, Xin C, Yuefeng C, et al. Stage-oriented Comprehensive Acupuncture Treatment plus Rehabilitation Training for Apoplectic Hemiplegia. *J Tradit Chin Med* 2008;28(2):90-93.
13 Ochi M, Wada F, Saeki S, et al. Gait training in subacute non-ambulatory stroke patients using a full weight-bearing gait-assistance robot: A prospective, randomized, open, blinded-endpoint trial. *J Neurol Sci* 2015;353:130-136.

14 Rosewilliam S, Malhotra S, Roffe C, et al. Can surface neuromuscular electrical stimulation of the wrist and hand combined with routine therapy facilitate recovery of arm function in patients with stroke? *Arch Physl Med Rehab* 2012;93(10):1715-1721.
15 Sanchez-Sanchez ML, Belda-Lois JM, Horno SM, et al. Functional principal component analysis as a new methodology for the analysis of the impact of two rehabilitation protocols in functional recovery after stroke. *J Neuroeng Rehabil* 2014;11:134.
16 Tang Q, Tan L, Li B, et al. Early Sitting, Standing, and Walking in Conjunction With Contemporary Bobath Approach for Stroke Patients With Severe Motor Deficit. *Top Stroke Rehabil* 2014;21(2):120-127.
17 Bai YL, Hu YS, Wu Y, et al. Long-term three-stage rehabilitation intervention alleviates spasticity of the elbows, fingers, and plantar flexors and improves activities of daily living in ischemic stroke patients: a randomized, controlled trial. *Neuroreport* 2014;25:998-1005.
18 Calabro RS, Naro A, Russo M, et al. Do post-stroke patients benefit from robotic verticalization? A pilot-study focusing on a novel neurophysiological approach. *Restor Neurol and Neuros* 2015:33:671-681.
19 Chaiyawat P and Kulkantrakorn K. Effectiveness of home rehabilitation program for ischemic stroke upon disability and quality of life: A randomized controlled trial. *Clin Neurol Neuros* 2012:114:866-870.
20 Chaiyawat P and Kulkantrakorn K. Randomized controlled trial of home rehabilitation for patients with ischemic stroke: impact upon disability and elderly depression. *Psychogeriatrics* 2012;12:193-199.
21 Jongbloed L, Stacey S and Brighton C. Stroke Rehabilitation: Sensorimotor Integrative Treatment Versus Functional Treatment. *Am J Occup Ther* 1989;43(6):391-397.
22 Kwakkel G, Wagenaar RC, Twisk JWR, et al. Intensity of leg and arm training after primary middle-cerebral artery stroke: a randomised trial. *Lancet* 1999;354:191-196.
23 Kwakkel G, Kollen BJ and Wagenaar RC. Long term effects of intensity of upper and lower limb training after stroke: a randomised trial. *J Neurol Neurosur Ps* 2002;72:473-479.
24 Kwakkel G and Wagenaar RC. Effect of Duration of Upper- and Lower-Extremity Rehabilitation Sessions and Walking Speed on Recovery of Interlimb Coordination in Hemiplegic Gait. *Phys Ther* 2002;82(5):432-448.
25 Morone G, Bragoni M, Iosa M, De Angelis D, Venturiero V, Coiro P. Who May Benefit From Robotic- Assisted Gait Training? A Randomized Clinical Trial in Patients With Subacute Stroke. *Neurorehab Neural Re* 2011;25(7):636-644.
26 Morone G, Iosa M, Bragoni M, De Angelis D, Venturiero V, Coiro P. Who May Have Durable Benefit From Robotic Gait Training? A 2-Year Follow-Up Randomized Controlled Trial in Patients With Subacute Stroke. *Stroke* 2012;43:1140-1142.
27 Yang XH, Bing L and Ba-si O. Effect of acupuncture combined with rehabilitative training on neural functional recovery of stroke patients during recovery phase: a randomized controlled trial. *World J Acupunct Mox* 2014;24(4):17-23.
28 Yue S, Jian X and Wong T. Effects of a nurse-led acupressure programme for stroke patients in China. *J Clin Nurs* 2012;22:1182-1188.

29 Rodrigues TA, Goroso DG, Westgate PM, et al. Slow Versus Fast Robot-Assisted Locomotor Training After Severe Stroke. A Randomized Controlled Trial. *Am J Phys Med Rehab* 2017;96(Suppl):S165–S170.
30 Sackley CM, Walker MF, Burton CR, et al. An occupational therapy intervention for residents with stroke related disabilities in UK care homes (OTCH): cluster randomised controlled trial. *BMJ* 2015;350:h468.
31 Volpe BT, Lynch D, Rykman-Berland A, et al. Intensive Sensorimotor Arm Training Mediated by Therapist or Robot Improves Hemiparesis in Patients With Chronic Stroke. *Neurorehabi Neural Re* 2008;22:305-310.
32 Zhang HY and Li PF. Observation on efficacy of acupuncture combined with rehabilitation training for post-stroke balance disorders. *World J Acupunct Mox* 2014;24(2):25-29.
