# Supplementary material for: The effect of rehabilitation interventions on physical function and immobility-related complications in severe stroke: a systematic review
Source: BMJ Open. 2020 Feb 5;10(2):e033642. doi: 10.1136/bmjopen-2019-033642 (PMC7045156; doi:10.1136/bmjopen-2019-033642)
Supplement: Author's manuscript [file bmjopen-2019-033642.draft_revisions.pdf]

BMJ Open

BMJ Open is committed to open peer review. As part of this commitment we make the peer review history of every article we publish publicly available.

When an article is published we post the peer reviewers' comments and the authors' responses online. We also post the versions of the paper that were used during peer review. These are the versions that the peer review comments apply to.

The versions of the paper that follow are the versions that were submitted during the peer review process. They are not the versions of record or the final published versions. They should not be cited or distributed as the published version of this manuscript.

BMJ Open is an open access journal and the full, final, typeset and author-corrected version of record of the manuscript is available on our site with no access controls, subscription charges or pay-per-view fees (http://bmjopen.bmj.com).

If you have any questions on BMJ Open's open peer review process please email info.bmjopen@bmj.com

BMJ Open

The effect of rehabilitation interventions on physical function and immobility-related complications in severe stroke- a systematic review

Journal:	*BMJ Open*
Manuscript ID	bmjopen-2019-033642
Article Type:	Research
Date Submitted by the Author:	14-Aug-2019

Complete List of Authors:	McGlinchey, Mark; King's College School, School of Population Health and Environmental Sciences; Guy's and Saint Thomas' Hospitals NHS Trust, Physiotherapy Department<br>James, Jimmy; Guy's and Saint Thomas' Hospitals NHS Trust, Physiotherapy Department<br>McKevitt, Christopher; King's College School, School of Population Health and Environmental Sciences<br>Douiri, Abdel; King's College School, School of Population Health and Environmental Sciences<br>Sackley, Catherine; King's College School, School of Population Health and Environmental Sciences

Keywords:	STROKE MEDICINE, PHYSIOTHERAPY, OCCUPATIONAL THERAPY, REHABILITATION MEDICINE

SCHOLARONE™
Manuscripts

The effect of rehabilitation interventions on physical function and immobility-related complications in severe stroke- a systematic review

Authors
Mark P McGlinchey [1,2], Jimmy James [2], Christopher McKevitt [1], Abdel Douiri [1], Catherine M Sackley [1]

1- School of Population Health and Environmental Sciences, Faculty of Life Sciences and Medicine, King's College London, London, SE1 1UL, England
2- Physiotherapy Department, St Thomas' Hospital, Guy's and St Thomas' NHS Foundation Trust, London, England

Corresponding Author
Mark P McGlinchey
School of Population Health and Environmental Sciences, Faculty of Life Sciences and Medicine, 6th floor Addison House, Guy's Campus, King's College London, London, SE1 1UL, England
Email address: mark.p.mcglinchey@kcl.ac.uk
Telephone: 0207 278 3456

Word Count- 4,068

For peer review only

For peer review only - http://bmjopen.bmj.com/site/about/guidelines.xhtml

ABSTRACT

Objective: To evaluate the effectiveness of rehabilitation interventions on physical function and immobility-related complications in severe stroke.

Design: Systematic review of electronic databases (MEDLINE, EMBASE, CINAHL, AMED, PEDro, DORIS, CENTRAL) searched between January 1987 and November 2018.
Methods: The PRISMA statement guided the review. Randomised controlled trials comparing the effect of one type of rehabilitation intervention to another or usual care on physical function and immobility-related complications for patients with severe stroke were included. Studies that recruited participants with all levels of stroke severity were included only if subgroup analysis based on stroke severity was performed. Two reviewers screened search results, selected studies using pre-defined selection criteria, extracted data and assessed risk of bias for selected studies using piloted proformas. Marked heterogeneity prevented meta-analysis and a descriptive review was performed. The GRADE approach was used to assess the strength of the evidence.

Results: 28 studies (n=2,677, mean age 72.7 years, 49.3% male) were included in the review. 24 studies were rated low or very low quality due to high risk of bias and small sample sizes. There was high quality evidence that very early mobilisation and OT intervention in care homes were no more effective than usual care. There was moderate quality evidence supporting short-term benefits of wrist and finger neuromuscular electrical stimulation in improving wrist extensor and grip strength, additional upper limb training on improving upper limb function and additional lower limb on improving upper limb function, independence in activities of daily living, and gait speed and independence.

Conclusions: There is a paucity of high-quality evidence to support the use of rehabilitation interventions to improve physical function and reduce immobility-related complications after severe stroke. Future research investigating more commonly used rehabilitation interventions, particularly to reduce post-stroke complications, is required.

PROSPERO registration number: CRD42017077737

Keywords: stroke rehabilitation, physiotherapy, occupational therapy

For peer review only - http://bmjopen.bmj.com/site/about/guidelines.xhtml

STRENGTHS AND LIMITATIONS OF THIS STUDY

- This is the first systematic review to investigate rehabilitation interventions specifically to survivors of severe stroke
- The review included outcomes on immobility-related post-stroke complications, which contribute to high levels of caregiver burden
- Marked heterogeneity of included studies prevented meta-analysis
- Most included studies were rated as low or very low-quality evidence due to unclear or high risk of bias as well as recruitment of very small samples

For peer review only

For peer review only - http://bmjopen.bmj.com/site/about/guidelines.xhtml

INTRODUCTION

Despite advances in stroke management over recent decades, stroke remains one of the most common causes of death and disability globally.|1,2| The mainstay of treating stroke is stroke rehabilitation, which aims to enable a person to achieve their optimal physical, cognitive, communicative, emotional and social level of function.|3-5| Rehabilitation of physical function comprises a large component of stroke rehabilitation programmes delivered by health-care professionals, such as physiotherapists and occupational therapists.6-8 Whilst several systematic reviews support the use of rehabilitation interventions to improve aspects of physical function, such as motor function, balance, walking speed and activities of daily living,|9-11| it is not clear from these reviews if these interventions are effective for survivors of differing levels of stroke severity, particularly severe stroke.

Severe stroke can be understood as a stroke resulting in a significant amount of brain tissue damage and multiple neurological impairments, which leads to a significant loss of function and residual disability.|12| Dependent upon how it is measured, 14 - 31% of people who sustain a stroke globally are classified as having a severe stroke,|13-18| a cohort of the stroke population that experiences worse outcomes compared to survivors of less severe stroke.|19-30| In the initial hospitalisation phase post-stroke, they are more likely to develop acute medical complications, which are negatively associated with functional recovery.|19| Three month mortality can be as high as 40%, compared to just under 5% for those patients with mild stroke.|20-22| Survivors of severe stroke pend longer in hospital, resulting in increased hospital costs, and demonstrate slower and less functional recovery, resulting in greater dependency when they are discharged from hospital.|14,15,23,25| For those discharged from hospital, survivors of severe stroke are at least eight times more likely to be discharged to a nursing home.|25,26| Longer-term care costs, which mostly support survivors of severe stroke, represent 49% of total stroke care spending globally.|27| In the first year post severe stroke, mortality can be as high as 60%|20| and survivors of severe stroke also experience very high levels of immobility-related complications, such as falls, contracture, pain, and pressure sores.|28,29| Due to this residual disability, the physical assistance provided by caregivers to look after survivors of

For peer review only - http://bmjopen.bmj.com/site/about/guidelines.xhtml

severe stroke as well as the psychosocial and emotional impact of the stroke on caregivers result in high levels of caregiver burden.|30|

As there are a number of significant issues faced by survivors of severe stroke, rehabilitation of severe stroke should focus on addressing these poor outcomes, particularly reduced physical function and its associated complications. However, the extent to which rehabilitation can address these outcomes is not clear. A previous systematic review demonstrated positive benefits of inpatient stroke rehabilitation, such as reduced mortality and hospital length of stay, and uncertain benefit on improving functional recovery.|31| However, this review did not explore the effect of specific interventions delivered within inpatient rehabilitation on improving physical function or on reducing immobility-related complications. Most trials investigating the efficacy of rehabilitation interventions on physical function have either not recruited survivors of severe stroke or not reported results specifically for survivors of severe stroke.|9-11| Therefore, it is not known if research findings are applicable to survivors of severe stroke. It is not clear whether rehabilitation should focus more on functional restoration, which may be incomplete or not possible, or reducing immobility-related complications, which may lessen longer-term burden for caregivers of severe stroke survivors. Due to this lack of clarity, there is an urgent need to summarise evidence-based rehabilitation interventions designed to optimise physical function and reduce immobility-related complications for this cohort of the stroke population.

This systematic review aims to establish the effectiveness of rehabilitation interventions on physical function and immobility-related complications for survivors of severe stroke and identify areas for future rehabilitation research for these patients.

METHODS

The systematic review has been reported according to the Preferred Reporting Items for Systematic Reviews and Meta-analysis (PRISMA) statement (see supplementary file 1).|32| The protocol for the systematic review has been published previously.|33|

For peer review only - http://bmjopen.bmj.com/site/about/guidelines.xhtml

Study design

The systematic review included randomised controlled trials (RCTs). The systematic review excluded quasi-experimental, correlational and descriptive study designs. Studies were selected according to the PICO (participant, intervention, comparator and outcome) format. The systematic review protocol provides full details of the PICO components.| 33|

Search strategy

Information sources

Electronic searches of the following databases were conducted: MEDLINE, EMBASE, Cumulative Index to Nursing and Allied Health Literature (CINAHL), Allied and Complementary Medicine Database (AMED), Physiotherapy Evidence Database (PEDro), Database of Research in Stroke (DORIS) and the Cochrane Central Register of Controlled Trials (CENTRAL). An example search strategy is shown in supplementary file 2. Databases were searched from January 1987 to November 2018. The search timeframe was guided by a scoping review of the literature (demonstrating very few published RCTs before 2000) and a consideration to include studies reflecting current clinical practice. Ongoing studies were identified by searching the Stroke Trials Registry (www.strokecenter.org/trials/) and clinicaltrials.gov. These sources were searched from 2012 to 2018 as it was assumed that studies before these dates would have been completed and published. References from included studies were hand searched and any potentially relevant study was included for review. Forward citation checks of included studies were also performed. To avoid language or cultural bias, studies in any language or geographical location were included.

Data management and study selection

The results from the literature search were uploaded to a reference management programme (Refworks) and duplicate references were removed. A final list of non-duplicated references was generated by one author (MM). The titles and abstracts of the search results were screened independently by two review authors (MM and JJ) and full text articles were obtained for relevant studies. Full text articles were reviewed by the same two authors (MM and JJ) independently to determine if studies met the inclusion criteria using an inclusion/exclusion checklist previously piloted. Any difference in opinion between the

For peer review only - http://bmjopen.bmj.com/site/about/guidelines.xhtml

two authors were resolved by a third review author (CS). Two review authors (MM and JJ) independently performed data extraction for all eligible articles using a data extraction proforma previously piloted.

Risk of bias and quality assessment

Risk of bias was assessed by two review authors independently (MM and JJ) using the Cochrane Collaboration tool for assessing the risk of bias across six main domains (sequence generation, allocation concealment, blinding, incomplete outcome data selective outcome reporting, other bias) .|34| A risk of bias judgement of 'high', 'low' or 'unclear' was determined for each of these main domains. The strength of evidence was assessed using the Grading of Recommendations Assessment, Development and Evaluation (GRADE) approach.|34| The five domains considered by the GRADE approach included risk of bias, inconsistencies between studies, indirectness, imprecision and publication bias. The quality of the evidence was ranked high, medium, low or very low by two review authors independently (MM and JJ).

Data analysis

As stated in the systematic review protocol, it was decided that if more than five adequately powered studies demonstrate homogeneity in terms of rehabilitation interventions and outcomes, results for individual outcomes would be pooled quantitatively using meta-analysis. Due to the limited number and marked heterogeneity in rehabilitation interventions and outcomes of the selected studies, it was not appropriate to undertake a meta-analysis. Therefore, a descriptive review of results was performed. As there may be differences in recovery rates and outcomes according to the time post-stroke, studies were grouped into three timeframes post-stroke determined on the basis of when participants were recruited to the study and when the study finished. These timeframes were the acute to early subacute stage (up to 3 months post-stroke), acute to late-subacute stage (up to 6 months post-stroke) and chronic stage (greater than 6 months post-stroke). These timeframes were chosen based on recommendations for the standardised measurement of sensorimotor recovery in stroke trials.|35| Study findings were presented according to these three timeframes.

For peer review only - http://bmjopen.bmj.com/site/about/guidelines.xhtml

RESULTS

The initial literature review identified 7589 articles (Figure 1). After removing duplicates and screening titles and abstracts, 1083 full text articles were assessed for eligibility. 28 studies were included in the systematic review.[36-67] 2677 participants were recruited to these studies- mean participant age was 72.7 years and 49.3% were male. The main reasons for excluding studies were due to not recruiting participants with severe stroke, not providing results separately for participants with severe stroke or not providing sufficient information to determine if the participants had sustained a severe stroke.

The characteristics of the included studies are provided as tables in the supplementary file 2. 16 studies were completed within the acute-early subacute phase, eight studies were completed within the acute-late subacute phase and four studies were completed within the chronic phase post-stroke. 20 different interventions were evaluated across the 28 studies. The assessment of risk of bias for each study is presented in Figure 2.

Outcomes

60 measures of physical function and immobility-related post-stroke complications were identified across the studies. The measures were classified as measures of body function (n=18), activity (n=26), participation (n=8) and post-stroke complications (n=8). These measures were grouped together as 16 different outcomes:

- Body function: cardiorespiratory function, neurological impairment, sensorimotor function
- Activity: activities of daily living (ADLs), balance and postural control, gait, general physical activity, upper limb function
- Participation: extended ADLs, perceived health status, quality of life
- Complications: caregiver burden, depression, mortality, shoulder pain/dislocation, spasticity

For each outcome, there was usually only one study investigating the effectiveness of a specific rehabilitation intervention in each time frame post-stroke. Most of these studies were rated as providing very low or low-quality evidence for these outcomes (see supplementary file 2). Outcomes which were supported by studies providing moderate or

For peer review only - http://bmjopen.bmj.com/site/about/guidelines.xhtml

high quality of evidence are reported in this section. Forest plots are included for key outcomes, although effect sizes were not estimable for studies that did not provide raw data and were not pooled due to heterogeneity in rehabilitation outcomes.

Body function

Sensorimotor Function

Seventeen studies evaluated changes in sensorimotor function. Ten studies were completed in the acute to early subacute phase post-stroke,|38-40,42,43,45-49| five studies were completed in the acute to late subacute phase post-stroke|53,56,60,62,63| and two studies were completed in the chronic phase post-stroke.|64,66| The most frequently used outcome measures of sensorimotor function were the Fugl-Meyer Assessment, used in 11 studies, and the MRC scale for muscle strength, used in 5 studies. Figure 3 provides a visual representation of the studies' effect sizes.

In the acute to early subacute phase post-stroke, there was moderate quality evidence from one study that a 6-week course of neuromuscular electrical stimulation (NMES) applied to the wrist and finger extensors in conjunction with usual therapy resulted in no improvement in wrist active movement compared to usual therapy.|49| Wrist strength and grip strength improved in the NMES group during the treatment period although these improvements were not evident at the 9-month follow-up.

Activity

Activities of Daily Living

Twenty studies explored independence and ability to perform activities of daily living (ADLs). Eleven studies were completed in the acute to early subacute phase,|36,37,41-43,45-50| seven studies were completed in acute to late subacute phase|52,54-57,60-63| and two studies were completed in the chronic phase.|65,67| Eighteen studies used the Barthel Index as the main outcome measure to assess independence in ADLs. Four studies used the Modified Rankin Scale and three studies used the Functional Independence Measure. Figure 4 provides a visual representation of studies' effect sizes.

In the acute to early subacute phase, there was high quality evidence that frequent, higher dose, very early mobilisation commencing within 24 hours post-stroke did not result in more patients being less dependent in ADLs at 3 months post-stroke compared to usual care,

For peer review only - http://bmjopen.bmj.com/site/about/guidelines.xhtml

which traditionally started more than 24 hours post-stroke.|36| However, caution is required with interpreting this finding as the sub-group analysis of patients with severe stroke was not powered for this outcome. There was moderate quality evidence that a 6-week course of NMES applied to the wrist and finger extensors in conjunction with usual therapy resulted in no difference in ADL independence compared to usual care.|49|

In the acute to late subacute phase, there was moderate quality evidence that additional LL therapy in conjunction with regular physical rehabilitation performed in the first 20 weeks post-stroke improved ADL independence whilst the intervention was being delivered when compared to regular physical rehabilitation alone.|57| However, these improvements were not seen 6 months post-stroke.

In the chronic phase, there was high quality evidence that a 3-month OT intervention provided to residents in care homes resulted in no difference in ADL independence compared to usual care.|65| Similar caution is required with interpreting this finding as the sub-group analysis of patients who were severely or very severely disabled was not powered for this outcome.

Gait

Nine studies investigated gait, which included gait ability and gait speed. Six studies were performed in the acute to early subacute phase,|38-40,43,45,48| two studies were performed in the acute to late subacute phase|57,60| and one study was performed in the chronic phase.|64| The Functional Ambulation Classification was used in eight studies, making it the most frequently used outcome measure of gait ability. The 10-metre walk test was used in five studies, making it the most frequently used outcome measure of gait speed. Figure 5 provides a visual representation of studies' effect sizes.

Only one study demonstrated moderate quality evidence. In the acute to late subacute phase, additional LL therapy in conjunction with regular physical rehabilitation performed in the first 20 weeks post-stroke improved gait ability and speed when compared to regular physical rehabilitation alone.|57| However, these improvements were not seen 6 months post-stroke.

For peer review only - http://bmjopen.bmj.com/site/about/guidelines.xhtml

General Physical Activity

Eight studies examined the effects of different interventions on improving general physical activity. Six studies were performed in the acute to early subacute phase,|37,38,40,45,46,51| one study was performed in the acute to late subacute phase|60| and one study was performed in the chronic phase.|65| General physical activity was defined as a composite of multiple physical tasks completed within one assessment, such as upper or lower limb function, transfers, gait and balance. Outcome measures used to assess general physical activity included the Rivermead Mobility Index, Rivermead Mobility Assessment and Motor Assessment Scale. Only one study demonstrated high quality evidence. In the chronic phase, a 3-month OT intervention provided to residents in care homes resulted in no difference in physical activity compared to usual care.|65|

Upper Limb Function

Four studies investigated changes in upper limb function,|46,49,57,66| of which two provided moderate quality evidence.|49,57| In the acute to early subacute phase, a 6-week course of NMES applied to the wrist and finger extensors in conjunction with usual therapy resulted in no difference in upper limb function compared to usual care.|49| In the acute to late subacute phase, additional UL or LL therapy in conjunction with regular physical rehabilitation performed in the first 20 weeks post-stroke improved upper limb function 6 months post-stroke when compared to regular rehabilitation.|57|

Participation

Extended Activities of Daily Living

Five studies investigated the effect of different interventions on extended ADLs,|37,38,44,56,57| of which one provided moderate quality evidence. In the acute to late subacute phase, additional UL or LL therapy in conjunction with regular physical rehabilitation performed in the first 20 weeks post-stroke improved performance in extended ADLs 6 months post-stroke when compared to regular rehabilitation.|57|

Quality of Life

Three studies examined quality of life,|54,57,65| of which two were moderate or high quality.|57,65| In the acute to late subacute phase, there was moderate quality evidence

For peer review only - http://bmjopen.bmj.com/site/about/guidelines.xhtml

that there was no benefit of additional UL or LL therapy to regular physical rehabilitation performed in the first 20 weeks post-stroke on improving quality of life 6 months post-stroke.|57| In the chronic phase, there was high quality evidence that a 3-month OT intervention provided to residents in care homes resulted in no difference in quality of life compared to usual care.|65|

Complications

Depression

Four studies explored changes in depression,|37,55,65,66| of which one was high quality.|65| In the chronic phase, a 3-month OT intervention provided to residents in care homes resulted in no difference in depression compared to usual care.

Mortality

One study investigated the effect of very early mobilisation on mortality.|36| There was high quality evidence that frequent, higher dose, very early mobilisation commencing within 24 hours post-stroke did not result in more patients dying at 3 months when compared to usual care, which traditionally started more than 24 hours post-stroke.

Other Outcomes

There was low quality of evidence for cardiorespiratory function (2 studies)|39,43| and caregiver burden (1 study).|37| There was very low to low quality of evidence for neurological impairment (3 studies),|41,60,62| balance and postural control (8 studies),|37,40,45,51,53,60,64,67| perceived health status (2 studies),|37,66| shoulder pain and dislocation (1 study),|66| and spasticity (6 studies).|38,43,46,52,60,66| Further details of these outcome and studies are included in the supplementary file 2.

DISCUSSION

Main Findings

Although 28 RCTs investigating 20 different rehabilitation interventions were identified in this review, there was a paucity of high-quality evidence to support the use of these interventions to improve physical function and reduce immobility-related complications after severe stroke. Most studies were rated as low or very low-quality evidence due to

For peer review only - http://bmjopen.bmj.com/site/about/guidelines.xhtml

unclear or high risk of bias as well as recruitment of very small samples. The majority of these studies were single centre RCTs, further reducing their generalisability to wider clinical practice. Only two large, multicentre studies were rated as high quality.|36,65| In both studies, results suggested that the treatment intervention was no more effective than usual care practice. However, patients with severe stroke or severe disability post-stroke comprised a smaller sample within these larger trials. Analyses of data from these sub-groups may not be powered to detect changes between the treatment and usual care interventions and therefore caution is required in interpreting the studies' findings.

In the AVERT trial,|36| very early and frequent mobilisation commencing within 24 hours post-stroke did not result in more patients being less dependent in ADLs or dying at 3 months post-stroke compared to usual care, which traditionally started more than 24 hours post-stroke. However, there was a trend in the data towards favouring usual care practice for patients with severe stroke. It could be argued that patients with severe stroke, who often present with multiple physical and cognitive impairments, may be less likely tolerate very early and intensive therapy in the first few days after stroke. This would suggest that mobilising patients less intensively after 24 hours may be more beneficial than very early and frequent mobilisation. In the OT in care home trial,|65| a 3-month, goal-orientated OT intervention for stroke survivors living in care homes did not result in improved ADL ability, quality of life or reduced depression up to 1-year post-intervention. The authors hypothesised that the lack of treatment effect may have been due to the care home residents' disability severity, which may have limited their engagement in therapy. However, a content analysis of the OT intervention by the research team revealed that the mean number of OT visits over the period was 5.1 (SD 3.0), the median session time was 30 minutes (IQR 15-60 minutes) and only 15% of OT time was used to provide ADL and mobility training. Although session length and duration were dependent upon the care home resident's ability to engage, it is possible that a more frequent OT intervention that focussed more on ADL and mobility training may have resulted in different findings.

Implications for Practice and Research

In light of these findings, it may be necessary to re-evaluate the design of future trials investigating rehabilitation interventions in severe stroke. As it is not known if survivors of severe stroke respond to interventions in the same ways as survivors of milder stroke, there

For peer review only - http://bmjopen.bmj.com/site/about/guidelines.xhtml

may be a need for more proof of concept studies to understand the mechanisms of recovery in severe stroke more fully. The high number of small, low-quality, single-centre RCTs investigating a broad range of interventions may suggest that larger, high-quality multi-centre RCTs investigating fewer interventions are warranted. However, outcome evaluations alone are insufficient to understand why certain interventions do or do not work. It is recommended that evaluations of complex interventions, such as stroke rehabilitation, use process evaluations alongside outcome evaluations.|68| Process evaluations enable an understanding of how to implement an intervention as well as how participants respond to and interact with the intervention. Therefore, future trials should be guided by more proof of concept research and involve both outcome and process evaluations.

In this review, the most frequently investigated outcomes were functional tasks, such as ADLs and gait ability. However, Pereira et al. has suggested that individuals with severe stroke are likely to make limited functional improvement with inpatient rehabilitation in the their review of rehabilitation after severe stroke.|31| They also advocated more focus on discharge planning and reducing post-stroke complications during inpatient rehabilitation for patients with severe stroke. Whilst the extent to which patients can improve functionally after severe stroke is not clear, there is merit in further exploring the effect of rehabilitation in the prevention and management of post-stroke complications in severe stroke. Sackley et al. investigated the prevalence of immobility-related complications in the first year after severely disabling stroke and found a very high prevalence of falls, contractures, pain and pressure sores.|28| However, with the exception of spasticity, there was very little focus on the prevention or management of post-stroke complications in the studies selected for our systematic review. In addition to a lack of focus on immobility-related complications, only one study explored caregiver burden, known to be very high amongst carers looking after survivors of severe stroke.|30| Future research in the rehabilitation of severe stroke should therefore focus more on the effectiveness of rehabilitation interventions in the prevention and management of immobility-related complications in severe stroke.

This review identified several studies investigating technological interventions, such as treadmill training and robot-assistive devices, and more novel interventions, such as thermal stimulation. However, it is not clear how commonly used these interventions are in clinical practice. Additionally, there were no trials studies of interventions commonly used

For peer review only - http://bmjopen.bmj.com/site/about/guidelines.xhtml

with survivors of severe stroke, such as positioning, sitting balance and seating.|69| This mismatch between available research evidence, which may not reflect current practice, and clinical practice, which may have limited research evidence to support its use, may present a dilemma for therapists, who are expected to base healthcare decisions on the best available and relevant evidence.|70| Therefore, future research is required to understand what interventions are currently being used in clinical practice. Knowledge of currently used rehabilitation interventions may guide future trials investigating their efficacy in improving physical function and reducing immobility-related post-stroke complications.

Strengths and Limitations

In terms of strengths, this is the first systematic review to investigate rehabilitation interventions specifically to survivors of severe stroke, who tend to be underrepresented in stroke rehabilitation research, and the identification of topics for future rehabilitation research will hopefully guide much needed research for this cohort of the stroke population. As well, the outcomes of the review focussed on not just physical function but immobility-related post-stroke complications, which are known to be higher in the severe stroke population and contribute to high levels of caregiver burden.|28-30| In terms of limitations, it has been reported that the defining severe stroke is difficult due to different criteria used to classify severity.|71| The use of objective scores on validated outcome measures to classify stroke severity in our systematic review, necessary to ensure that participants had actually sustained a severe stroke, may have precluded the inclusion of studies that either used different scoring systems or outcome measures to classify stroke severity. However, these studies were discussed in detail amongst three review authors to determine suitability for inclusion and therefore it is likely that the number of relevant studies excluded from the review was minimal. Another limitation is the use of data from subgroups within larger clinical trials. As subgroup analyses may not be powered to detect changes between groups, caution is required in the interpretation of findings from these trials. In addition, raw subgroup data were not fully reported in some studies preventing estimation of effect sizes.

CONCLUSION

There was a paucity of high-quality evidence to support the use of rehabilitation interventions to improve physical function and reduced immobility-related complications

For peer review only - http://bmjopen.bmj.com/site/about/guidelines.xhtml

after severe stroke. Future research should be guided by more proof of concept studies and involve outcome and process evaluations to more fully understand the impact of different interventions on patients with severe stroke. Future research should investigate the effect of more clinically used interventions, such as positioning, sitting balance and seating. Future research should also investigate the effect of interventions on post-stroke complications known to be high after severe stroke, such as contracture, pressure sores and caregiver burden.

Authors' Contributions

MM is the guarantor of the review. MM, CS and CM were involved in the design of the protocol and systematic review. MM conducted scoping searches. MM and JJ piloted the inclusion/exclusion form. MM piloted the data extraction form. MM was the first reviewer and JJ was the second reviewer for the systematic review. AD provided statistical support for the systematic review. MM drafted the manuscript. All authors read and approved the final manuscript.

Competing Interests

None declared.

Funding

This project forms part of MM's PhD which is funded by The Dunhill Medical Trust [grant number RT62/0116]. The funder has had no input on the design of the protocol and will have no input on the analysis and interpretation of the results of the systematic review, or publication of the systematic review.

Patient consent

Not required.

Patient/public involvement

Patients or the public were not involved in the design, conduct, reporting or dissemination of this research.

For peer review only - http://bmjopen.bmj.com/site/about/guidelines.xhtml

REFERENCES

1 GBD 2016 Causes of Death Collaborators. Global, regional, and national age-sex specific mortality for 264 causes of death, 1980–2016: a systematic analysis for the Global Burden of Disease Study 2016. *Lancet* 2017;390:1151–1210.

2 GBD 2016 DALYs and HALE Collaborators. Global, regional, and national disability-adjusted life-years (DALYs) for 333 diseases and injuries and healthy life expectancy (HALE) for 195 countries and territories, 1990–2016: a systematic analysis for the Global Burden of Disease Study 2016. *Lancet* 2017;390:1260–1344.

3 National Institute for Health and Care Excellence. Stroke rehabilitation in adults, https://www.nice.org.uk/guidance/cg162 (2013, accessed 1 August 2019).

4 Hebert D, Lindsay MP, McIntyre A, et al. Canadian stroke best practice recommendations: Stroke rehabilitation practice guidelines, update 2015. *Int J Stroke* 2016;11(4):459-484.

5 Langhorne P, Bernhardt J and Kwakkel G. Stroke rehabilitation. *Lancet* 2011;377:1693–1702.

6 Bode R, Heinemann A, Semik P, et al. Patterns of Therapy Activity Across Length of Stay and Impairment Levels: Peering Inside the "Black Box" of Inpatient Stroke Rehabilitation. *Arch Phys Med Rehab* 2004;85:1901-1908.

7 De Wit L, Putman K, Lincoln N, et al. Stroke rehabilitation in Europe- what do physiotherapists and occupational therapists actually do? *Stroke* 2006;37:1483-1489.

8 Lang C, MacDonald J, Reisman D, et al. Observation of Amounts of Movement Practice Provided During Stroke Rehabilitation. *Arch Phys Med Rehab* 2009;90:1692-1698.

9 Veerbeek JM, van Wegen E, van Peppen R, et al. What is the evidence for physical therapy poststroke? A systematic review and meta-analysis. *PLoS One* 2014;9(2)e87987.

10 Pollock A, Baer G, Campbell P, et al. Physical rehabilitation approaches for the recovery of function and mobility following stroke. *Cochrane DB Syst Rev* 2014. doi: 10.1002/14651858.CD001920.pub3

11 Legg LA, Lewis SR, Schofield-Robinson OJ, et al. Occupational therapy for adults with problems in activities of daily living after stroke. *Cochrane DB Syst Rev* 2017, doi:10.1002/14651858.CD003585.pub3

12 Teasell R, Pereira S and Cotoi A. The Rehabilitation of Severe Stroke, http://www.ebrsr.com/sites/default/files/v18-SREBR-CH22-NET-1.pdf (2018, accessed 1 August 2019).

13 Kelly-Hayes M, Wolf PA, Kennel WB, et al. Factors influencing survival and need for institutionalization following stroke: the Framingham study. *Arch Phys Med Rehab* 1988;69:415-418.

14 Jorgensen HS, Nakayama H, Raaschou HO, et al. Outcome and time course of recovery in stroke. Part I: Outcome. The Copenhagen stroke study. *Arch Phys Med Rehab* 1995;76:399-405.

15 Jorgensen HS, Nakayama H, Rancho HO, et al. Outcome and time course of recovery in stroke. Part II: Time Course of Recovery. The Copenhagen stroke study. *Arch Phys Med Rehab* 1995;76:406-412.

16 Islam MS, Anderson CS, Hankey GJ, et al. Trends in incidence and outcome of stroke in Perth, Western Australia during 1989 to 2001. The Perth community stroke study. *Stroke* 2008;39:776-782.

For peer review only - http://bmjopen.bmj.com/site/about/guidelines.xhtml

17 Luengo-Fernandez R, Paul N, Gray A, et al. Population-Based Study of Disability and Institutionalization After Transient Ischemic Attack and Stroke 10-Year Results of the Oxford Vascular Study. *Stroke* 2013;44:2854-2861.
18 McGlinchey MP, Paley L, Hoffman A, et al. Physiotherapy provision to hospitalised stroke patients: analysis from the UK Sentinel Stroke National Audit Programme. *Eur Stroke J* 2019;4(1):75-84
19 Kim BR, Lee, J, Sohn MK, Kim DY, Lee SG, Shin YI et al. Risk factors and functional impact of medical complications in stroke. *Ann Rehabil Med* 2017;41(5):753-760.
20 Bamford J, Santrock P, Dennis M, et al. Classification and natural history of clinically identifiable subtypes of cerebral infarction. *Lancet* 1991;337:1521-1526.
21 Heuschmann P, Kolominsky-Rabas P, Misselwitz B, et al. Predictors of In-Hospital Mortality and Attributable Risks of Death After Ischemic Stroke: The German Stroke Registers Study Group. *Arch Intern Med* 2004;1761-1768.
22 Bhaskar S, Stanwell P, Bivard A, et al. The influence of initial stroke severity on mortality, overall functional outcome and in-hospital placement at 90 days following acute ischemic stroke: A tertiary hospital stroke register study. *Neurol India* 2017;65(6):1252-1259.
23 Xu XM, Vestesson E, Paley L, et al. The economic burden of stroke care in England, Wales and Northern Ireland: Using a national stroke register to estimate and report patient-level health economic outcomes in stroke. *Eur Stroke J* 2018;3(1):82-91.
24 Douiri A, Grace J, Sarker SJ, et al. Patient-specific prediction of functional recovery after stroke. *Int J Stroke* 2017;12(5):539-548
25 Saxena SK, Ng TP, Yong D, et al. Total direct cost, length of hospital stay, institutional discharges and their determinants from rehabilitation settings in stroke patients. *Acta Neurol Scand* 2006;114:307-314.
26 McKenna K, Tooth L, Strong J, et al. Predicting discharge outcomes for stroke patients in Australia. *Am J Phys Med Rehab* 2002;81:47-56.
27 Evers SM, Struijs JN, Ament AJ, et al. International comparison of stroke cost studies. *Stroke* 2004;35:1209–1215
28 Sackley C, Brittle N, Patel S, et al. The prevalence of joint contractures, pressure sores, painful shoulder, other pain, falls and depression in the year after a severely disabling stroke. *Stroke* 2008;39:3329-3334.
29 Langhorne P, Stott DJ, Robertson L, et al. Medical Complications After Stroke: A Multicentre Study. *Stroke* 2000;31:1223-1229.
30 Rigby H, Gubitz G and Phillips S. A systematic review of caregiver burden following stroke. *Int J Stroke* 2009;4:285-292.
31 Pereira S, Graham JR, Shahabaz A, et al. Rehabilitation of individuals with severe stroke: synthesis of best evidence and challenges in implementation. *Top Stroke Rehabil* 2012;19(2),122-131.
32 Moher D, Liberati A, Tetzlaff J, et al. Preferred reporting Items for systematic reviews and meta-Analyses: The PRISMA statement. *PLoS Med* 2009;6(7):e1000097.
33 McGlinchey MP, James J, McKevitt C, et al. The effect of rehabilitation interventions of physical function and immobility-related complications in severe stroke- protocol for a systematic review. *Syst Rev* 2018;7:197
34 Higgins JPT and Green S. Cochrane handbook for systematic reviews of interventions Version 5.1.0. The Cochrane Collaboration, http://handbook-5-1.cochrane.org/ (2011, Accessed 1 August 2019).

For peer review only - http://bmjopen.bmj.com/site/about/guidelines.xhtml

35 Kwakkel G, Lannin NA, Borschmann K, et al. Standardised measurement of sensorimotor recovery in stroke trials: consensus-based core recommendations from the Stroke Recovery and Rehabilitation Roundtable. *Int J Stroke* 2017;12:451-461.
36 AVERT Trial Collaboration group. Efficacy and safety of very early mobilisation within 24 h of stroke onset (AVERT): a randomised controlled trial. *Lancet* 2015;386:46-55.
37 Bagley P, Hudson M, Forster A, et al. A randomized trial evaluation of the Oswestry Standing Frame for patients after stroke. *Clin Rehabil* 2005;19:354-364.
38 Bradley L, Hart BB, Mandana S, et al. Electromyographic biofeedback for gait training after stroke. *Clin Rehabil* 1998;12:11-22.
39 Chang WH, Kim MS, Huh JP, et al. Effects of Robot-Assisted Gait Training on Cardiopulmonary Fitness in Subacute Stroke Patients: A Randomized Controlled Study. *Neurorehab Neural Re* 2012;26(4):318-324.
40 Chen JC, Lin CH, Wei YC, et al. Facilitation of motor and balance recovery by thermal intervention for the paretic lower limb of acute stroke: a single-blind randomized clinical trial. *Clin Rehabil* 2011;25(9):823-832.
41 Di Lauro A, Pellegrino L, Savastano G, et al. A randomized trial on the efficacy of intensive rehabilitation in the acute phase of ischemic stroke. *J Neurol* 2003;250:1206-1208.
42 Fong K, Yang N, Chan M, et al. Combined effects of sensory cueing and limb activation on unilateral neglect in subacute left hemiplegic stroke patients: a randomized controlled pilot study. *Clin Rehabil* 2013;27(7):628-637.
43 Franceschini M, Carda S, Agosti M, et al. Walking After Stroke: What Does Treadmill Training With Body Weight Support Add to Overground Gait Training in Patients Early After Stroke? A Single-Blind, Randomized, Controlled Trial. *Stroke* 2009;40:3079-3085.
44 Katz-Leurer M, Carmeli E and Shochina M. The effect of early aerobic training on independence six months post stroke. *Clin Rehabil* 2003;17:735-741.
45 Liang CC, Hsieh TC, Lin CH, et al. Effectiveness of Thermal Stimulation for the Moderately to Severely Paretic Leg After Stroke: Serial Changes at One-Year Follow-Up. *Arch Phys Med Rehab* 2012;93:1903-1910.
46 Lincoln NB, Parry RH and Vass CD. Randomized, Controlled Trial to Evaluate Increased Intensity of Physiotherapy Treatment of Arm Function After Stroke. *Stroke* 1999;30:573-579.
47 Min M, Xin C, Yuefeng C, et al. Stage-oriented Comprehensive Acupuncture Treatment plus Rehabilitation Training for Apoplectic Hemiplegia. *J Tradit Chin Med* 2008;28(2):90-93.
48 Ochi M, Wada F, Saeki S, et al. Gait training in subacute non-ambulatory stroke patients using a full weight-bearing gait-assistance robot: A prospective, randomized, open, blinded-endpoint trial. *J Neurol Sci* 2015;353:130-136.
49 Rosewilliam S, Malhotra S, Roffe C, et al. Can surface neuromuscular electrical stimulation of the wrist and hand combined with routine therapy facilitate recovery of arm function in patients with stroke? *Arch Physl Med Rehab* 2012;93(10):1715-1721.
50 Sanchez-Sanchez ML, Belda-Lois JM, Horno SM, et al. Functional principal component analysis as a new methodology for the analysis of the impact of two rehabilitation protocols in functional recovery after stroke. *J Neuroeng Rehabil* 2014;11:134.

For peer review only - http://bmjopen.bmj.com/site/about/guidelines.xhtml

51 Tang Q, Tan L, Li B, et al. Early Sitting, Standing, and Walking in Conjunction With Contemporary Bobath Approach for Stroke Patients With Severe Motor Deficit. *Top Stroke Rehabil* 2014;21(2):120-127.
52 Bai YL, Hu YS, Wu Y, et al. Long-term three-stage rehabilitation intervention alleviates spasticity of the elbows, fingers, and plantar flexors and improves activities of daily living in ischemic stroke patients: a randomized, controlled trial. *Neuroreport* 2014;25:998-1005.
53 Calabro RS, Naro A, Russo M, et al. Do post-stroke patients benefit from robotic verticalization? A pilot-study focusing on a novel neurophysiological approach. *Restor Neurol and Neuros* 2015:33:671-681.
54 Chaiyawat P and Kulkantrakorn K. Effectiveness of home rehabilitation program for ischemic stroke upon disability and quality of life: A randomized controlled trial. *Clin Neurol Neuros* 2012:114:866-870.
55 Chaiyawat P and Kulkantrakorn K. Randomized controlled trial of home rehabilitation for patients with ischemic stroke: impact upon disability and elderly depression. *Psychogeriatrics* 2012;12:193-199.
56 Jongbloed L, Stacey S and Brighton C. Stroke Rehabilitation: Sensorimotor Integrative Treatment Versus Functional Treatment. *Am J Occup Ther* 1989;43(6):391-397.
57 Kwakkel G, Wagenaar RC, Twisk JWR, et al. Intensity of leg and arm training after primary middle-cerebral artery stroke: a randomised trial. *Lancet* 1999;354:191-196.
58 Kwakkel G, Kollen BJ and Wagenaar RC. Long term effects of intensity of upper and lower limb training after stroke: a randomised trial. *J Neurol Neurosur Ps* 2002;72:473-479.
59 Kwakkel G and Wagenaar RC. Effect of Duration of Upper- and Lower-Extremity Rehabilitation Sessions and Walking Speed on Recovery of Interlimb Coordination in Hemiplegic Gait. *Phys Ther* 2002;82(5):432-448.
60 Morone G, Bragoni M, Iosa M, De Angelis D, Venturiero V, Coiro P. Who May Benefit From Robotic- Assisted Gait Training? A Randomized Clinical Trial in Patients With Subacute Stroke. *Neurorehab Neural Re* 2011;25(7):636-644.
61 Morone G, Iosa M, Bragoni M, De Angelis D, Venturiero V, Coiro P. Who May Have Durable Benefit From Robotic Gait Training? A 2-Year Follow-Up Randomized Controlled Trial in Patients With Subacute Stroke. *Stroke* 2012;43:1140-1142.
62 Yang XH, Bing L and Ba-si O. Effect of acupuncture combined with rehabilitative training on neural functional recovery of stroke patients during recovery phase: a randomized controlled trial. *World J Acupunct Mox* 2014;24(4):17-23.
63 Yue S, Jian X and Wong T. Effects of a nurse-led acupressure programme for stroke patients in China. *J Clin Nurs* 2012;22:1182-1188.
64 Rodrigues TA, Goroso DG, Westgate PM, et al. Slow Versus Fast Robot-Assisted Locomotor Training After Severe Stroke. A Randomized Controlled Trial. *Am J Phys Med Rehab* 2017;96(Suppl):S165–S170.
65 Sackley CM, Walker MF, Burton CR, et al. An occupational therapy intervention for residents with stroke related disabilities in UK care homes (OTCH): cluster randomised controlled trial. *BMJ* 2015;350:h468.
66 Volpe BT, Lynch D, Rykman-Berland A, et al. Intensive Sensorimotor Arm Training Mediated by Therapist or Robot Improves Hemiparesis in Patients With Chronic Stroke. *Neurorehabi Neural Re* 2008;22:305-310.

For peer review only - http://bmjopen.bmj.com/site/about/guidelines.xhtml

67 Zhang HY and Li PF. Observation on efficacy of acupuncture combined with rehabilitation training for post-stroke balance disorders. *World J Acupunct Mox* 2014;24(2):25-29.
68 Moore G, Audrey S, Barker M, et al. Process evaluation of complex interventions: Medical Research Council guidance. *BMJ* 2015;350:h1258.
69 McGlinchey M, Walmsley N and Cluckie G. Positioning and pressure care. In: Bhalla A, Birns J,eds. Management of Post-Stroke Complications. Switzerland: Springer 2015:189-225.
70 Dawes M, Summerskill W, Glasziou P, et al. Sicily statement on evidence-based practice. *BMC Med Educ* 2005;5(1) doi:10.1186/1472-6920-5-1.
71 Peters S, Ivanona TD, Teasell R, et al. Is the Recovery of Functional Balance and Mobility Accompanied by Physiological Recovery in People With Severe Impairments After Stroke? *Neurorehab Neural Re* 2014;28(9):847-855.

For peer review only

FIGURE LEGENDS

Figure 1- Flow chart of studies

Figure 2- Risk of bias of individual domains in the included studies

Figure 3- Interventions for sensorimotor function

Figure 4- Interventions for ADL ability and independence

Figure 5- Interventions for gait ability and speed

For peer review only - http://bmjopen.bmj.com/site/about/guidelines.xhtml

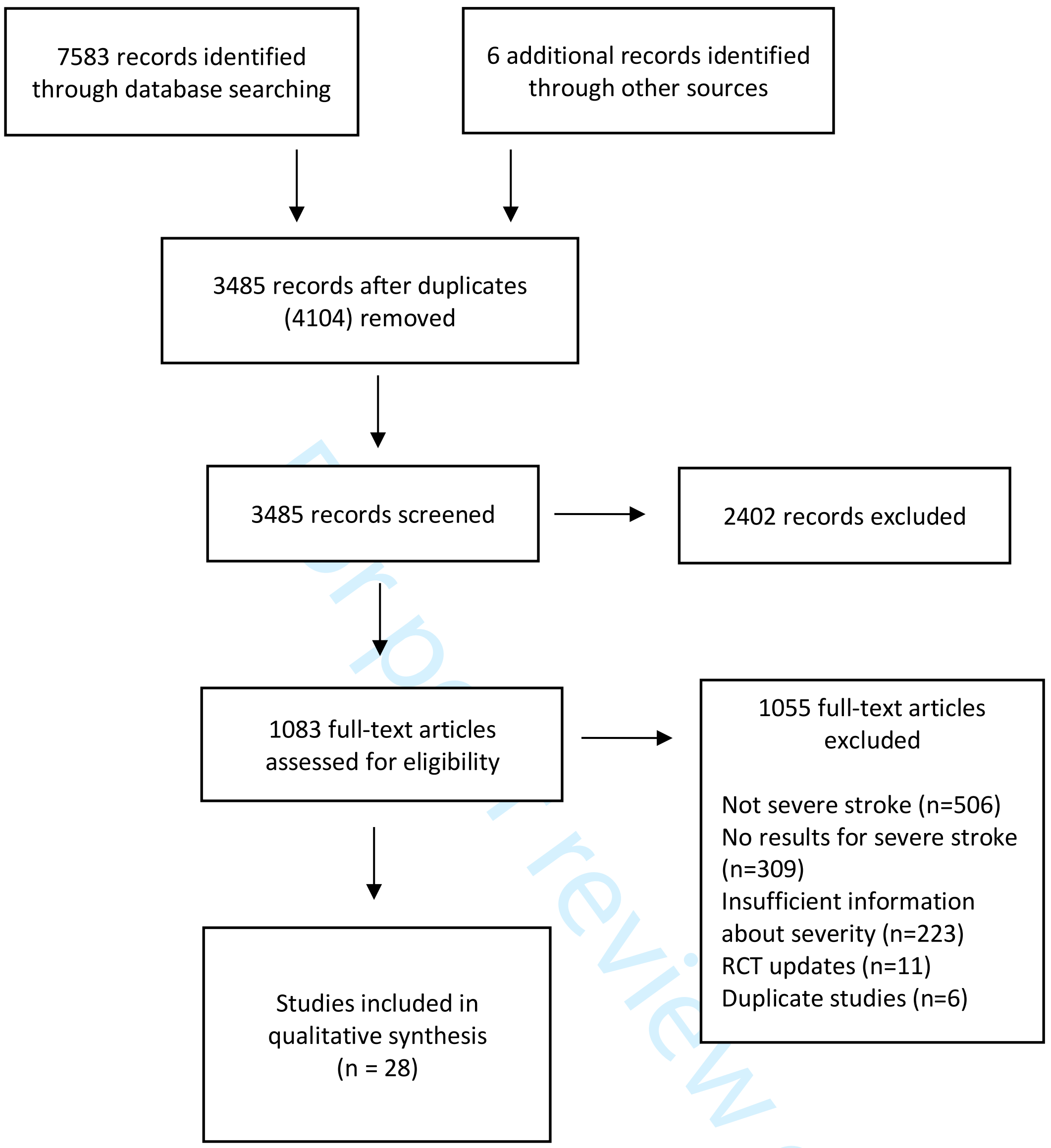

For peer review only

	Random sequence generation (selection bias)	Allocation concealment (selection bias)	Blinding of participants and personnel (performance bias)	Blinding of outcome assessment (detection bias)	Incomplete outcome data (attrition bias)	Selective reporting (reporting bias)	Other bias
AVERT Triallists' Colloboration 2015	+	+	+	+	+	+	?
Bagley et al. 2005	+	+	−	+	+	?	−
Bai et al. 2014	+	?	−	+	+	−	−
Bradley et al. 1998	+	?	−	+	?	?	+
Calabro et al. 2015	+	?	?	+	?	?	+
Chaiyawat and Kulkantrakorn 2012	+	+	−	−	+	?	−
Chang et al. 2012	+	+	?	+	+	?	+
Chen et al. 2011	+	+	?	+	+	?	+
di Lauro et al. 2003	+	+	?	+	+	?	−
Fong et al. 2013	+	+	?	+	+	?	+
Franceschini et al. 2009	+	?	?	+	−	?	?
Jongbloed et al. 1989	+	?	+	+	?	?	+
Katz-Leurer et al. 2003	+	?	?	+	?	?	+
Kwakkel et al. 1999	+	+	+	+	+	?	+
Liang et al. 2012	+	+	?	+	+	?	+
Lincoln et al. 1999	+	+	−	+	?	?	+
Min et al. 2008	?	?	−	?	?	?	+
Morone et al. 2011	+	?	?	+	+	?	+
Ochi et al. 2015	+	+	?	+	+	?	+
Rodrigues et al. 2017	+	+	?	+	?	?	+

For peer review only - http://bmjopen.bmj.com/site/about/guidelines.xhtml

Rosewilliam et al. 2012	+	+	?	+	+	?	?
Sackley et al. 2015	+	+	+	+	+	+	+
Sanchez-Sanchez et al. 2014	+	?	−	+	?	?	−
Tang et al. 2014	+	+	+	+	+	?	+
Volpe et al. 2008	?	?	?	+	+	?	+
Yang et al. 2014	+	?	−	?	+	?	+
Yue et al. 2012	+	+	−	?	+	?	+
Zhang and Li 2014	−	?	−	?	?	?	+

Study or Subgroup	Experimental Mean	Experimental SD	Experimental Total	Control Mean	Control SD	Control Total	Std. Mean Difference IV, Random, 95% CI
**1.3.1 Acute to early subacute**							
Bradley et al. 1998	0	0	7	0	0	6	Not estimable
Chang et al. 2012	22.7	5.7	20	19.6	5.6	17	0.54 [−0.12, 1.20]
Chen et al. 2011	14	3.7	17	6	5.04	16	1.77 [0.95, 2.59]
Fong et al. 2013	12	11.3	19	10.6	9.5	16	0.13 [−0.54, 0.80]
Franceschini et al. 2009	59	30.37	52	76	22.59	42	−0.62 [−1.04, −0.20]
Liang et al. 2012	22.2	1.1	15	16.1	1.1	15	5.40 [3.77, 7.03]
Lincoln et al. 1999	11	36.3	94	0	25.19	95	0.35 [0.06, 0.64]
Min et al. 2008	75.37	10.02	30	64.21	8.83	30	1.17 [0.62, 1.72]
Ochi et al. 2015	9	8.89	13	9	7.41	13	0.00 [−0.77, 0.77]
Rosewilliam et al. 2012	10.2	16.2	39	6.3	10.3	41	0.29 [−0.15, 0.73]
**1.3.2 Acute to late subacute**							
Calabro et al. 2015	25	6	10	19	7	10	0.88 [−0.05, 1.81]
Jongbloed et al. 1989	0	0	7	0	0	12	Not estimable
Morone et al. 2011	0	0	12	0	0	12	Not estimable
Yang et al. 2014	37.55	2.09	33	28.71	4.07	31	2.72 [2.03, 3.42]
Xue et al. 2012	65.93	24.05	35	58.79	26.17	34	0.28 [−0.19, 0.76]
**1.3.3 Chronic**							
Rodrigues et al. 2017	20	1.48	10	18	3.7	8	0.71 [−0.26, 1.67]
Volpe et al. 2008	15.73	2	11	15.1	1.7	10	0.32 [−0.54, 1.19]

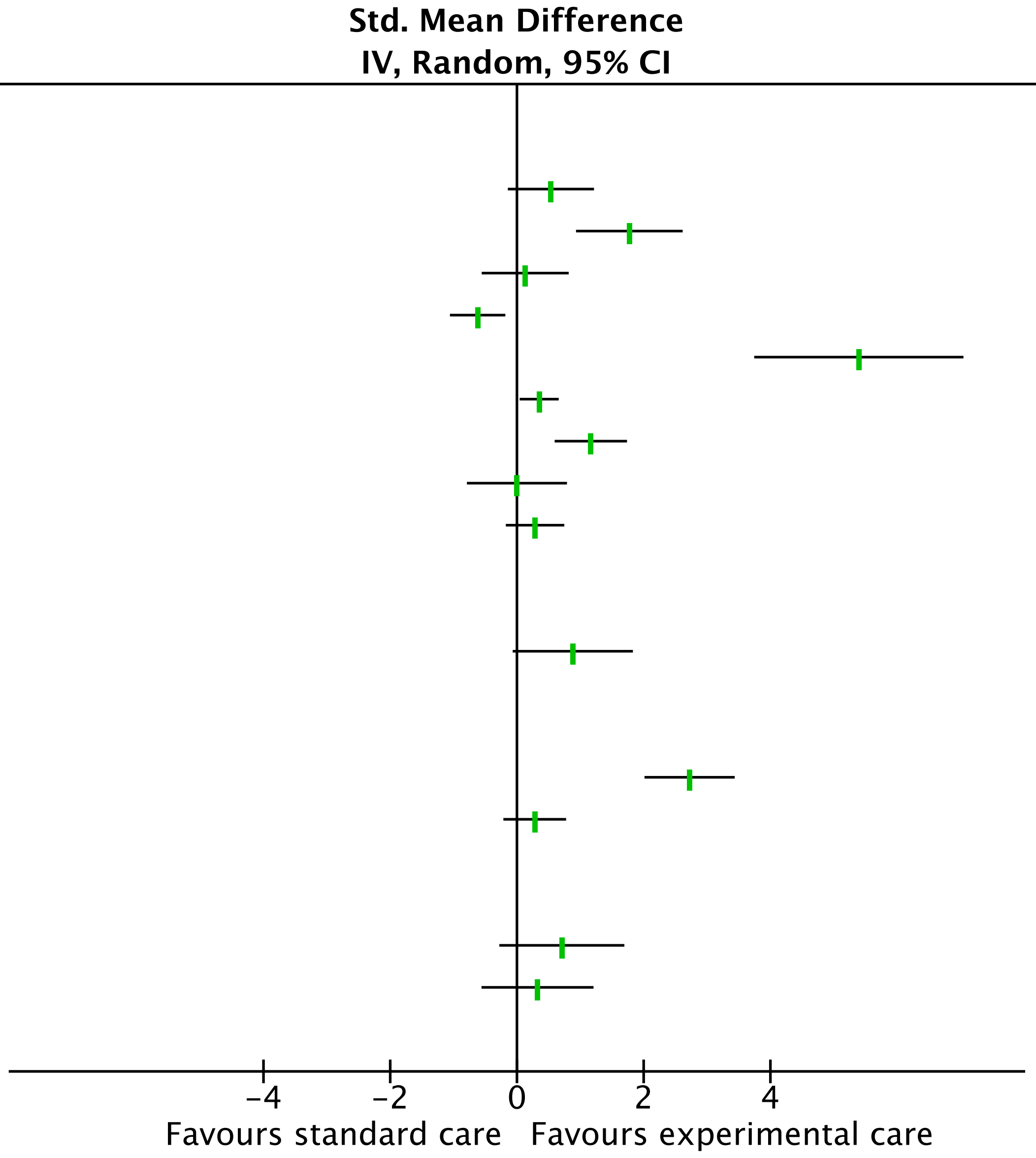

For peer review only

Study or Subgroup	Experimental Mean	Experimental SD	Experimental Total	Control Mean	Control SD	Control Total	Std. Mean Difference IV, Random, 95% CI	Year
**1.1.1 Acute to early subacute**								
Lincoln et al. 1999	12	5.93	94	13	7.41	95	−0.15 [−0.43, 0.14]	1999
di Lauro et al. 2003	3.2	2	26	3.2	2.6	27	0.00 [−0.54, 0.54]	2003
Bagley et al. 2005	3.2	4.3	65	2.89	3.6	67	0.08 [−0.26, 0.42]	2005
Min et al. 2008	80.78	12.79	30	60.08	11.92	30	1.65 [1.06, 2.24]	2008
Franceschini et al. 2009	13	5.56	52	12	5.56	45	0.18 [−0.22, 0.58]	2009
Liang et al. 2012	76.7	3.7	15	64.7	5	15	2.65 [1.64, 3.67]	2012
Rosewilliam et al. 2012	5.4	4	39	5.8	5.2	41	−0.09 [−0.52, 0.35]	2012
Fong et al. 2013	42.8	14.9	19	47.7	14.9	16	−0.32 [−0.99, 0.35]	2013
Sanchez–Sanchez et al. 2014	0	0	5	0	0	8	Not estimable	2014
Ochi et al. 2015	13	5.93	13	13	3.7	13	0.00 [−0.77, 0.77]	2015
AVERT Triallists' Colloboration 2015	0	0	147	0	0	144	Not estimable	2015
**1.1.2 Acute to late subacute**								
Jongbloed et al. 1989	54.3	0	7	54.6	0	12	Not estimable	1989
Kwakkel et al. 1999	19	2.96	31	16	6.67	37	0.56 [0.07, 1.04]	1999
Morone et al. 2011	69.6	15.1	12	52.1	14.1	12	1.16 [0.28, 2.03]	2011
Chaiyawat and Kulkantrakorn 2012	97.2	2.8	30	76.4	9.4	28	3.00 [2.24, 3.77]	2012
Yue et al. 2012	70.03	26.92	35	61.46	24.09	34	0.33 [−0.14, 0.81]	2012
Bai et al. 2014	82	5.93	77	56	9.63	77	3.24 [2.75, 3.72]	2014
Yang et al. 2014	63.76	4.65	33	53.82	4.35	31	2.18 [1.55, 2.80]	2014
**1.1.3 Chronic**								
Zhang and Li 2014	61.33	18.47	30	51.72	17.28	29	0.53 [0.01, 1.05]	2014
Sackley et al. 2015	0	0	397	0	0	338	Not estimable	2015

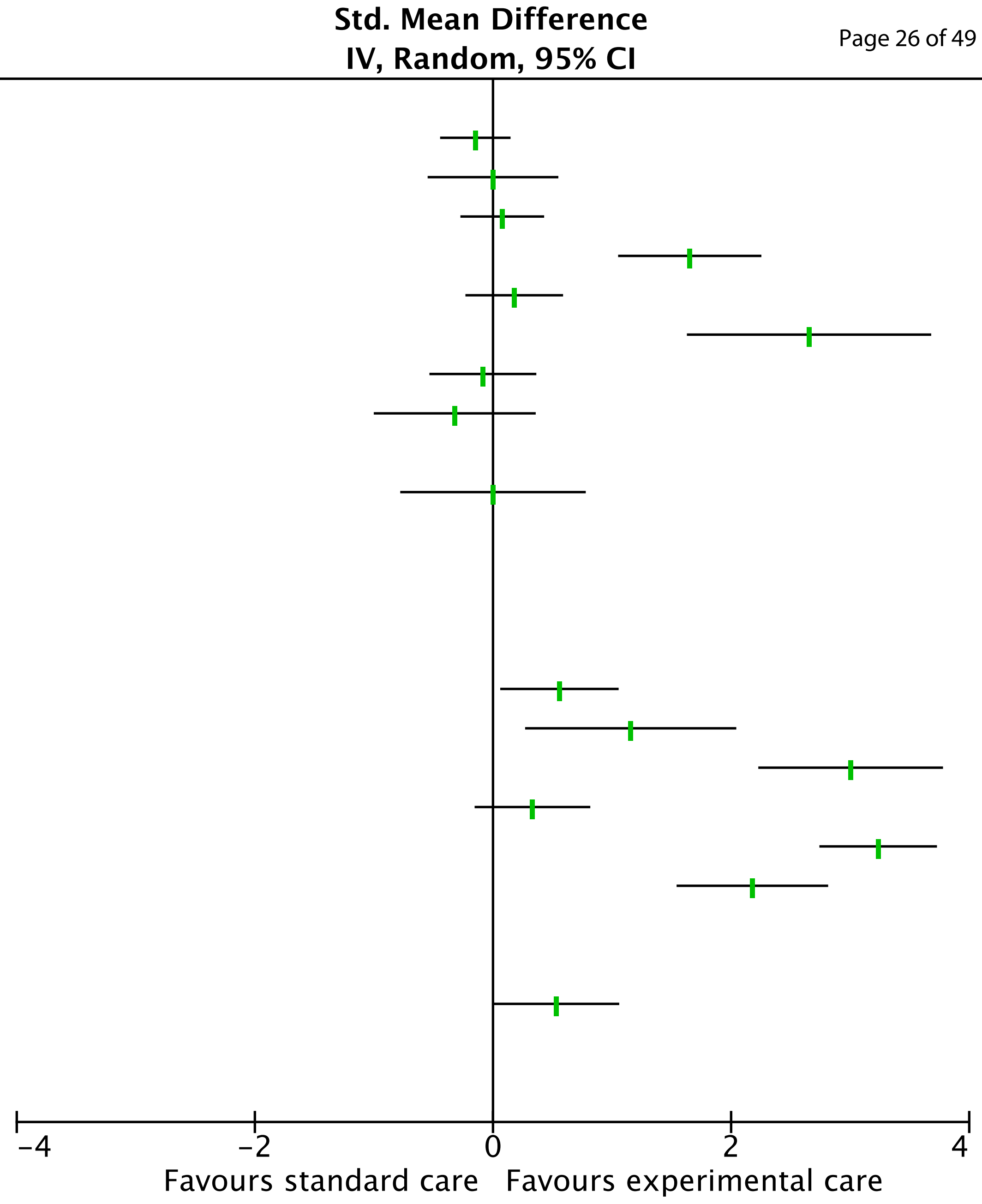

For peer review only

Study or Subgroup	Experimental Mean	Experimental SD	Experimental Total	Control Mean	Control SD	Control Total	Std. Mean Difference IV, Random, 95% CI
**1.2.1 Acute to early subacute**							
Bradley et al. 1998	0	0	6	0	0	4	Not estimable
Chang et al. 2012	1.3	0.7	20	1.4	0.8	17	−0.13 [−0.78, 0.52]
Chen et al. 2011	2	0.37	17	1	0.37	16	2.64 [1.67, 3.60]
Franceschini et al. 2009	3	1.48	52	2	1.85	42	0.60 [0.18, 1.02]
Liang et al. 2012	2.6	0.1	15	1.9	0.2	15	4.31 [2.94, 5.68]
Ochi et al. 2015	3	0.74	13	3	0.37	13	0.00 [−0.77, 0.77]
**1.2.2 Acute to late subacute**							
Kwakkel et al. 1999	4	1.48	31	3	2.22	37	0.52 [0.03, 1.00]
Morone et al. 2011	2.6	0.9	12	1.2	0.8	12	1.59 [0.65, 2.53]
**1.2.3 Chronic**							
Rodrigues et al. 2017	2.6	0.2	10	2.1	0.4	8	1.57 [0.47, 2.66]

Std. Mean Difference
IV, Random, 95% CI

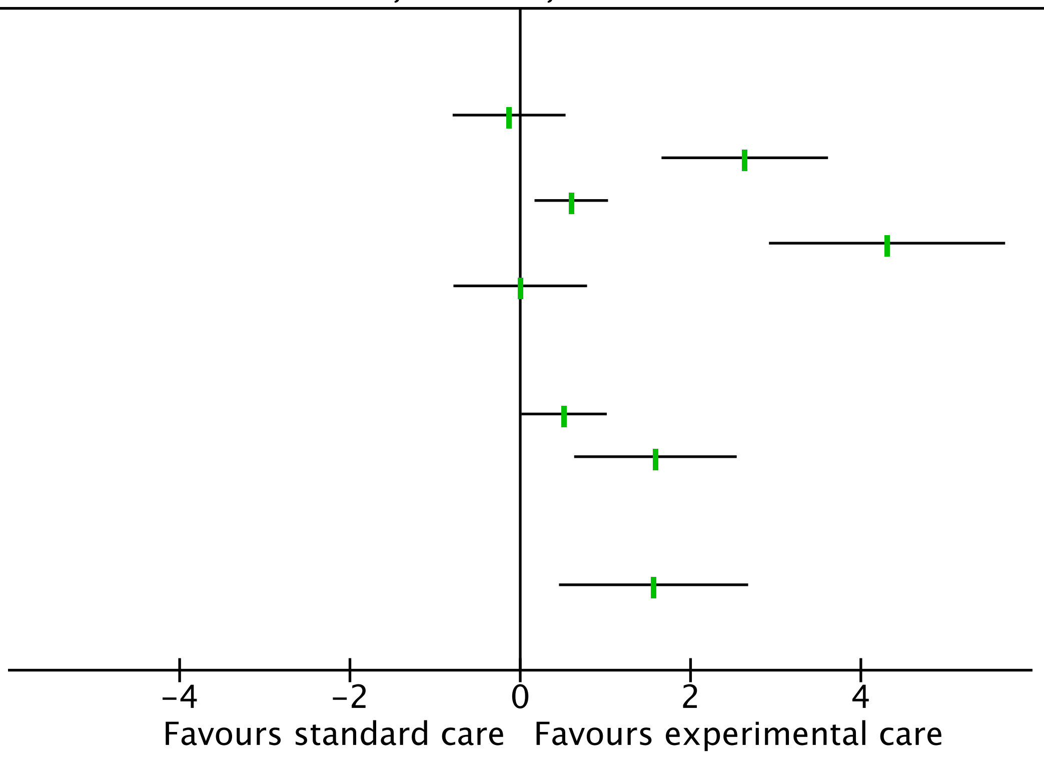

For peer review only

PRISMA 2009 Checklist

Section/topic	#	Checklist item	Reported on page #
**TITLE**			
Title	1	Identify the report as a systematic review, meta-analysis, or both.	Title page
**ABSTRACT**			
Structured summary	2	Provide a structured summary including, as applicable: background; objectives; data sources; study eligibility criteria, participants, and interventions; study appraisal and synthesis methods; results; limitations; conclusions and implications of key findings; systematic review registration number.	Abstract page
**INTRODUCTION**			
Rationale	3	Describe the rationale for the review in the context of what is already known.	1-2
Objectives	4	Provide an explicit statement of questions being addressed with reference to participants, interventions, comparisons, outcomes, and study design (PICOS).	2
**METHODS**			
Protocol and registration	5	Indicate if a review protocol exists, if and where it can be accessed (e.g., Web address), and, if available, provide registration information including registration number.	Abstract page, 2
Eligibility criteria	6	Specify study characteristics (e.g., PICOS, length of follow-up) and report characteristics (e.g., years considered, language, publication status) used as criteria for eligibility, giving rationale.	3
Information sources	7	Describe all information sources (e.g., databases with dates of coverage, contact with study authors to identify additional studies) in the search and date last searched.	4
Search	8	Present full electronic search strategy for at least one database, including any limits used, such that it could be repeated.	Supplementary file
Study selection	9	State the process for selecting studies (i.e., screening, eligibility, included in systematic review, and, if applicable, included in the meta-analysis).	3, 4
Data collection process	10	Describe method of data extraction from reports (e.g., piloted forms, independently, in duplicate) and any processes for obtaining and confirming data from investigators.	3, 4
Data items	11	List and define all variables for which data were sought (e.g., PICOS, funding sources) and any assumptions and simplifications made.	3
Risk of bias in individual studies	12	Describe methods used for assessing risk of bias of individual studies (including specification of whether this was done at the study or outcome level), and how this information is to be used in any data synthesis.	4
Summary measures	13	State the principal summary measures (e.g., risk ratio, difference in means).	N/A
Synthesis of results	14	Describe the methods of handling data and combining results of studies, if done, including measures of consistency (e.g., I^2) for each meta-analysis.	N/A

For peer review only - http://bmjopen.bmj.com/site/about/guidelines.xhtml

PRISMA 2009 Checklist

Section/topic	#	Checklist item	Reported on page #
Risk of bias across studies	15	Specify any assessment of risk of bias that may affect the cumulative evidence (e.g., publication bias, selective reporting within studies).	N/A
Additional analyses	16	Describe methods of additional analyses (e.g., sensitivity or subgroup analyses, meta-regression), if done, indicating which were pre-specified.	N/A
**RESULTS**			
Study selection	17	Give numbers of studies screened, assessed for eligibility, and included in the review, with reasons for exclusions at each stage, ideally with a flow diagram.	5
Study characteristics	18	For each study, present characteristics for which data were extracted (e.g., study size, PICOS, follow-up period) and provide the citations.	Supplementary file
Risk of bias within studies	19	Present data on risk of bias of each study and, if available, any outcome level assessment (see item 12).	5
Results of individual studies	20	For all outcomes considered (benefits or harms), present, for each study: (a) simple summary data for each intervention group (b) effect estimates and confidence intervals, ideally with a forest plot.	Supplementary file, 6-9
Synthesis of results	21	Present results of each meta-analysis done, including confidence intervals and measures of consistency.	N/A
Risk of bias across studies	22	Present results of any assessment of risk of bias across studies (see Item 15).	N/A
Additional analysis	23	Give results of additional analyses, if done (e.g., sensitivity or subgroup analyses, meta-regression [see Item 16]).	N/A
**DISCUSSION**			
Summary of evidence	24	Summarize the main findings including the strength of evidence for each main outcome; consider their relevance to key groups (e.g., healthcare providers, users, and policy makers).	9-12
Limitations	25	Discuss limitations at study and outcome level (e.g., risk of bias), and at review-level (e.g., incomplete retrieval of identified research, reporting bias).	12
Conclusions	26	Provide a general interpretation of the results in the context of other evidence, and implications for future research.	12, 13
**FUNDING**			
Funding	27	Describe sources of funding for the systematic review and other support (e.g., supply of data); role of funders for the systematic review.	13

From: Moher D, Liberati A, Tetzlaff J, Altman DG, The PRISMA Group (2009). Preferred Reporting Items for Systematic Reviews and Meta-Analyses: The PRISMA Statement. PLoS Med 6(7): e1000097. doi:10.1371/journal.pmed1000097

For more information, visit: **www.prisma-statement.org**.

For peer review only - http://bmjopen.bmj.com/site/about/guidelines.xhtml

Supplementary File 2

Supplementary Material- Medline Search Strategy

1. exp Stroke/
2. severe stroke.mp.
3. stroke severit*.mp.
4. stroke disabilit*.mp.
5. exp Physical Therapy Modalities/
6. exp Occupational Therapy/
7. exp Nursing Care/
8. physical rehabilitation.mp.
9. exp Stroke Rehabilitation/
10. exp Patient Positioning/
11. exp Posture/
12. exp Exercise/
13. exp Exercise Therapy/
14. passive exercise.mp.
15. exp "Range of Motion, Articular"/
16. manual technique.mp.
17. active exercise.mp.
18. Resistance Training/
19. exp Muscle Stretching Exercises/
20. exp Electric Stimulation/
21. exp Electric Stimulation Therapy/
22. exp Wheelchairs/
23. seat?.mp.
24. exp "Equipment and Supplies"/
25. exp Teaching/
26. exp Education/
27. exp Motor Skills/
28. exp Movement/
29. motor function.mp.
30. motor recovery.mp.
31. exp "Recovery of Function"/
32. exp "Activities of Daily Living"/
33. functional independence.mp.
34. physical independence.mp.
35. complicatio*.mp.
36. exp Pain/
37. exp Contracture/
38. exp Pressure Ulcer/
39. exp Respiratory Tract Infections/
40. exp Urinary Tract Infections
41. Muscle Spasticity/
42. Venous Thrombosis/
44. exp Pulmonary Embolism/
44. exp Accidental Falls/
45. exp Fatigue/
46. exp Depression/
47. 1 or 2 or 3 or 4

For peer review only

48. 5 or 6 or 7 or 8 or 9 or 10 or 11 or 12 or 13 or 14 or 15 or 16 or 17 or 18 or 19 or 20 or 21 or 22 or 23 or 24 or 25 or 26
49. 27 or 28 or 29 or 30
50. 31 or 32 or 33 or 34
51. 35 or 36 or 37 or 38 or 39 or 40 or 41 or 42 or 43 or 44 or 45 or 46
52. 47 and 48 and 49
53. 47 and 48 and 50
54. 47 and 48 and 51
55. limit 52 to ("all adult (19 plus years)" and randomized controlled trial)
56. limit 53 to ("all adult (19 plus years)" and randomized controlled trial)
57. limit 54 to ("all adult (19 plus years)" and randomized controlled trial)

For peer review only

Supplementary Table 1- Studies conducted in the acute – early subacute (<3 months) phase post-stroke

Study	Intervention Description	Intervention Duration	Intervention Delivered By	Stroke Severity Measure	Sample Size and Characteristics	Main Outcome Measures	Main Results	Quality of Evidence
AVERT trial collaboration group 2015 [1]	Very early mobilisation vs Usual care	Up to 14 days	PT and nursing staff	NIHSS	Very early mobilisation group NIHSS >16 (n=147)<br><br>Usual care group NIHSS >16 (n=144)	Favourable outcome (mRS 0-2) and mortality at 3 months	No difference in favourable outcome or mortality between groups	High
Bagley et al. 2005 [2]	Oswestry standing frame + standard physiotherapy vs Standard physiotherapy	14 daily sessions	PTs	BI^	Oswestry group (n=71) Median BI 1 (IQR 0-3)<br><br>Control group (n=69) Median BI 2 (IQR 1-3)	RMI, BI, HADS, NEADL, RMA, MAS (balance, sit to stand sections), TCT, CSI, GHQ-28	No differences between groups for all outcome measures. No differences in number of treatment sessions between groups or number of staff members required to treat each patient.	Low
Bradley et al. 1998 [3]	EMG biofeedback + conventional physiotherapy vs Placebo EMG + conventional physiotherapy	6 weeks	PTs	RMI	EMG group RMI ≤3 (n=7)<br><br>Conventional PT group RMI ≤3 (n=6)	MBS, mAS, 10MWT, RMI, sensation, proprioception NEADL	No differences between groups for MBS, RMI, NEADL and 10MWT. No improvements in mAS, sensation and proprioception for both groups.	Very low
Chang et al. 2012 [4]	Robot-assisted BWS treadmill gait training + conventional physiotherapy vs Conventional physiotherapy	2 weeks	PTs	FAC<br>LL FMA	Robot-assisted group (n=20) Mean FAC 0.5 (SD 0.5) Mean LL FMA 17.2 (SD 5.5)<br><br>Conventional group (n=17) Mean FAC 0.4 (SD 0.5) Mean LL FMA 16.8 (SD 5.7)	FAC, LL MI, LL FMA, Peak VO_2	Improvements in LL FMA and peak VO_2 in robot-assisted gait training group. No improvements in LL MI and FAC for both groups.	Low

For peer review only

Chen et al. 2011 [5]	Thermal stimulation + standard rehabilitation vs Standard rehabilitation	6 weeks	Thermal stimulation- PTs<br><br>Standard rehabilitation- PTs and OTs	FAC<br>LL FMA	Thermal stimulation group (n=17)<br>Median FAC 0 (IQR 0-1)<br>Median LL FMA 7 (4-11.5)<br><br>Standard rehab group (n=16)<br>Median FAC 0 (IQR 0-1)<br>Median LL FMA 6 (4.3-12.0)	LL FMA, LL MRC, mAS, mMAS, PASS (trunk control items), BBS, FAC	Thermal stimulation group demonstrated greater recovery gains compared to standard rehabilitation group in all outcomes except PASS. No difference between groups in MAS.	Low
Di Lauro et al. 2003 [6]	Intensive rehabilitative treatment vs Ordinary rehabilitative treatment	14 days	Therapists and nursing staff	BI^	Intensive rehab group (n=29)<br>Mean BI 1.4 (SD 1.4)<br><br>Ordinary rehab group (n=31)<br>Mean BI 1.5 (SD 1.5)	BI, mNIHSS	No differences between groups in BI or mNIHSS	Very low
Fong et al. 2013 [7]	Cueing wristwatch + conventional rehabilitation vs Sham wristwatch + conventional rehabilitation	3 weeks	Wristwatch- OTs<br><br>Conventional rehab- OT, PT, ST	Motor FIM	Cueing wristwatch group (n=19) Mean motor FIM 25.6 (SD 8.3)<br><br>Sham wristwatch group (n=16)<br>Mean motor FIM 28.2 (SD 10.0)	UL FMA, FTHUE, motor FIM, total number of UL movements	No differences between groups for UL FMA, FTHUE and motor FIM. More total UL movements in cueing wristwatch group but not significantly different between groups.	Low
Franceschini et al. 2009 [8]	BWS treadmill gait training + conventional treatment vs Conventional treatment	4 weeks	PTs	BI^	Treadmill training group (n=52)<br>Median BI 6 (IQR 3-9)<br>Median FAC 0 (IQR 0-0)<br><br>Conventional group (n=45)<br>Median BI 5 (IQR 3-7)<br>Median FAC 0 (IQR 0-0)	MI, TCT, mRS, BI, FAC, AS, LL proprioception, 6MWT, 10MWT, BS, WHS	No differences between groups. All patients were able to walk at discharge.	Low

For peer review only

Katz-Leurer et al. 2003 [9]	Leg cycle ergometer + regular therapy<br>vs<br>Regular therapy	8 weeks	Leg cycle ergometer- PTs<br><br>Regular therapy- PT, OT, ST	SSS	Leg cycle ergometer and regular rehabilitation groups- actual number of patients with severe stroke (SSS <30) not reported	FAI	No differences in decline in FAI between groups	Low
Liang et al. 2012 [20]	Thermal stimulation + standard rehabilitation<br>vs<br>Standard rehabilitation	6 weeks	Thermal stimulation- PTs<br><br>Standard rehabilitation- PTs and OTs	BI*	Thermal stimulation group (n=15)<br>Mean BI 30.3 (SD 11.1)<br><br>Standard rehab group (n=15)<br>Mean BI 27.7 (SD 14.3)	LL FMA, LL MRC, FAC, BBS, mMAS, BI	Improvements in LL FMA, LL MRC, FAC and mMAS in thermal stimulation group post-intervention and at 3-month follow-up. Improvements in BBS and BI in thermal stimulation group only at 3-month follow-up. Except for LL-FMA, all improvements disappeared at 6-month and 12-month follow-up.	Low
Lincoln et al. 1999 [11]	Standard physiotherapy + additional qualified PT therapy<br>vs<br>Standard physiotherapy + additional PTA therapy<br>vs<br>Standard physiotherapy	5 weeks	PTs/ PTAs	BI^	Qualified PT group (n=94)<br>Median BI 6 (IQR 3-9)<br><br>PTA group (n=93)<br>Median BI 6 (IQR 4-8)<br><br>Standard PT group (n=95)<br>Median BI 7 (IQR 3-9)	RMA- arm scale, ARAT, THPT, grip strength, mAS, BI, MCA	No differences between the groups across all outcomes	Low
Min et al. 2008 [12]	Acupuncture + systemic functional exercise<br>vs<br>Systemic functional exercise	? 3 months	Not reported	BI*	Acupuncture group (n=30)<br>Mean BI 27.28 (SD 5.41)<br><br>Systemic exercise group (n=30)<br>Mean BI 28.01 (SD 4.48)	FMA, BI	Acupuncture group demonstrated greater improvements in FMA and BI compared to the systemic exercise group	Very low

For peer review only

Ochi et al. 2015 [13]	Robot-assisted treadmill gait training + standard physiotherapy<br>vs<br>Conventional overground gait training + standard physiotherapy	4 weeks	Robot-assisted gait training- not reported<br>Conventional gait training- PTs	FIM mobility<br>FAC	Robot-assisted group (n=13) Median FAC 0 (IQR 0-1)<br>Median FIM mobility 7 (IQR 6-10)<br><br>Conventional group (n=13)<br>Median FAC 1 (IQR 0-1)<br>Median FIM mobility 7 (IQR 7-9)	FAC, FMA, LL muscle torque, 10MWT, FIM (mobility scores)	Robot-assisted gait training group demonstrated greater improvements in FAC and peak LL muscle torque compared to the conventional group	Low
Rosewilliam et al. 2012 [14]	Wrist and finger NMES + usual care<br>vs<br>Usual care	6 weeks	NMES- staff group not reported, patients and carers<br><br>Usual care- PTs	BI^	NMES group (n=31)<br>Mean BI 4.4 (SD 3.9)<br>Mean ARAT 0.0 (SD 0.0)<br><br>Usual care group (n=36)<br>Mean BI 2.5 (SD 2.9)<br>Mean ARAT 0.6 (SD 3.5)	ARAT, BI, wrist AROM, wrist strength, grip strength	No differences in ARAT, BI or wrist AROM between groups. Improvements in wrist extensor and grip strength in the NMES group post-intervention but not maintained at follow-up.	Moderate
Sanchez-Sanchez et al. 2014 [15]	Functionally targeted physiotherapy techniques + conventional physiotherapy<br>vs<br>Conventional physiotherapy	Not reported	PTs	BI*	Functional techniques group (n=5)<br>Mean BI 13 (SD 10.95)<br><br>Conventional therapy group (n=8)<br>Mean BI 11.43 (SD 13.13)	BI	Functionally targeted physiotherapy group demonstrated greater improvement compared to the conventional physiotherapy group when using functional principal component analysis	Very low
Tang et al. 2014 [16]	Contemporary Bobath approach with early sitting, standing and walking<br>vs<br>Contemporary Bobath approach	8 weeks	PTs	STREAM<br>BBS	Early contemporary group (n=24)<br>Mean STREAM 1.4 (SD 1.0)<br>Mean BBS 0 (SD 0)<br><br>Contemporary group (n=24)<br>Mean STREAM 1.3 (SD 0.9)<br>Mean BBS 0 (SD 0)	STREAM, BBS	Improvements in STREAM and BBS in the contemporary Bobath approach with early mobilisation group	Low

For peer review only

Supplementary Table 2- Studies conducted in the acute – late subacute (<6 months) phase post-stroke

Study	Intervention Description	Intervention Duration	Intervention Delivered By	Stroke Severity Measure	Sample Size and Characteristics	Main Outcome Measures	Main Results	Quality of Evidence
Bai et al. 2014 [17]	Staged physical rehabilitation interventions + routine care vs Routine care	6 months	PTs and OTs	BI*	Staged rehab group (n=83)<br>Mean BI 28 (range 24-31)<br><br>Routine care group (n=82)<br>Mean BI 23 (range 19-27)	BI, mAS	Staged rehab group demonstrated higher BI scores than the routine care group at 1, 3- and 6-months post-stroke. 42.9% of patients in the routine care group demonstrated spasticity in at least one body part compared to 36.4% of patients in the staged rehab group.	Low
Calabrò et al. 2015 [18]	Robotic verticalisation + standard physiotherapy vs Physiotherapy-assisted verticalisation + standard physiotherapy	6 weeks	PTs	PASS<br>LL FMA	Robotic group (n=10)<br>Mean PASS 3 (SD 1)<br>Mean LL FMA 13 (SD 3)<br><br>Physiotherapy group (n=10)<br>Mean PASS 3 (SD 3)<br>Mean LL FMA 12 (SD 6)	PASS, LL FMA, MRC, vertical posture tolerance	Both interventions were well tolerated. Robotic group demonstrated greater improvements in MRC, LL FMA and PASS compared to the physiotherapy group	Very low
Chaiyawat and Kulkantrakorn 2012 [19,20]	Home based physiotherapy programme vs Usual care	6 months	PTs	BI*	Home PT group (n=30)<br>Mean BI 31.7 (SD 5.9)<br>Mean NIHSS 16.4 (SD 4.1)<br><br>Usual care group (n=30)<br>Mean BI 33.2 (SD 4.8)<br>Mean NIHSS 17.8 (SD 3.9)	BI, HADS, mRS, EQ-5D	Home therapy group demonstrated greater improvements in BI, HADS, mRS and EQ-5D compared to the usual care group which were maintained at 2-year follow-up.	Very low
Jongbloed et al. 1989 [21]	Functional treatment approach vs Sensorimotor integrative treatment approach	8 weeks	OTs	BI*	Functional treatment group (n=13)<br>Mean BI 31.5<br><br>Sensorimotor integrative treatment group (n=9)<br>Mean BI 30	BI, meal preparation, eight subtests of Sensorimotor Integration Test Battery	No differences between groups on all outcome measures	Very low

For peer review only

Kwakkel et al.<br>1999 [22]<br>2002 [23]<br>2002 [24]	Additional UL training + usual care<br>vs<br>Additional LL training + usual care<br>vs<br>UL/LL pressure splint immobilisation + usual care	20 weeks	PTs and OTs	BI^	UL training group (n=33)<br>Median BI 5 (IQR 3-7)<br>LL training (n=31)<br>Median BI 6 (IQR 3-8)<br>Splint control group (n=37)<br>Median BI 5.5 (IQR 3-7)	BI, FAC, ARAT, 10MWT, SIP, NHP, FAI	LL training group had significantly higher BI, FAC, walking speed and ARAT than splint control group post-intervention. UL training group had significantly higher ARAT than splint control group post-intervention. No significant differences in all outcomes were seen between groups from 6 months onwards up until 12-month follow-up.	Moderate
					CRP sub-study<br>UL training group (n=18)<br>Mean BI 5.0 (SD 2.0)<br>LL training (n=17)<br>Mean BI 6.3 (SD 2.7)<br>Splint control group (n=18)<br>Mean BI 5.3 (SD 2.7)	10MWT, mean CRP of arm/leg movements	LL training group had significantly higher comfortable walking speed than UL and splint control groups post-intervention. No differences were seen for the mean CRP of arm/leg movements between groups.	
Morone et al.<br>2011 [25]<br>2012 [26]	Robot-assisted BWS treadmill gait training + standard physiotherapy<br>vs<br>Conventional gait training + standard physiotherapy	3 months	PTs	BI*	Robotic groups<br>Low motricity (n=12)<br>Mean BI 14.2 (SD 11.8)<br>High motricity (n=12)<br>Mean BI 20.0 (SD 17.2)<br><br>Conventional groups<br>Low motricity (n=12)<br>Mean BI 7.9 (SD 8.9)<br>High motricity (n=12)<br>Mean BI 24.6 (SD 15.3)	FAC, LL AS, RMI, MI, TCT, CNS, BI, RS, 6MWT, 10MWT	Higher FAC in low motricity robotic training group compared to low motricity conventional training group post-intervention. At discharge, higher RMI, BI, TCT, RS and 6MWT in low motricity robotic training group compared to low motricity conventional training group. No differences were seen between the higher motricity groups post-intervention or on discharge.<br>At 12-month follow-up, low motricity robotic training group had higher FAC, BI and RMI compared to low motricity conventional training group. No differences were seen between the higher motricity groups.	Very low

For peer review only

Yang et al. 2014 [27]	Acupuncture + rehabilitation training vs Rehabilitation training	8 weeks	Acupuncture- not reported<br><br>Rehabilitation- PTs	NIHSS BI*	Acupuncture group (n=33)<br>Mean NIHSS 25.5 (SD 2.4)<br>Mean BI 39.4 (SD 3.9)<br><br>Rehabilitation group (n=31)<br>Mean NIHSS 24.1 (SD 3.1)<br>Mean BI 38.1 (SD 4.3)	NIHSS, FMA, BI	Acupuncture group demonstrated higher scores on all outcome measures compared to the rehabilitation group	Very low
Yue et al. 2012 [28]	Acupressure treatment + routine care vs Routine care	3 months	Nurses	BI*	Acupressure group (n=35)<br>Mean BI 26.8 (SD 15.2)<br><br>Routine care group (n=34)<br>Mean BI 24.4 (SD 16.8)	FMA, BI	Acupressure group demonstrated greater improvements in BI and FMA only at 3-month time frame	Very low

For peer review only

S3 Table- Studies conducted in the chronic (>6 months) phase post-stroke

Study	Intervention Description	Intervention Duration	Intervention Delivered By	Stroke Severity Measure	Sample Size and Characteristics	Main Outcome Measures	Main Results	Quality of Evidence
Rodrigues et al. 2017 [29]	Robot-assisted BWS treadmill gait training with progressively increased speeds<br>vs<br>Robot-assisted bodyweight supported treadmill gait training with progressively decreased speeds	6 weeks	Not reported	LL FMA<br>FAC	Faster speed group (n=10)<br>Median FAC 1.5 (1–2)<br>Mean LL FMA 19.5 (SD 4.6)<br><br>Slower speed group (n=10)<br>Median FAC 1 (1–2)<br>Mean LL FMA 17.5 (SD 2.8)	FAC, TUG, 6MWT, 10MWT, BBS, LL FMA	Improvements in FAC, FMA, TUG and 6MWT in the slower speed group compared to the faster speed group.	Very low
Sackley et al. 2015 [30]	OT intervention<br>vs<br>Usual care	3 months	OTs	BI^	OT intervention group-<br>BI 0-4 n=268<br>BI 5-9 n=129<br><br>Usual care group-<br>BI 0-4 n=234<br>BI 5-9 n=104	BI, RMI, GDS, EQ-5D-3L	No differences between the groups on any outcome measure at 3-, 6- and 12-months post-randomisation. Higher fall rate per resident in OT intervention group at 3 months.	High
Volpe et al. 2008 [31]	Intensive standard UL therapy<br>vs<br>Intensive robot-assisted UL therapy	6 weeks	Therapists	NIHSS	Therapist group (n=10)<br>Mean NIHSS 17 (SD 1)<br><br>Robot group (n=11)<br>Mean NIHSS 17 (SD 1)	FMA- UL, MRC-shoulder/ elbow, mAS, UL PROM, SIS, ARAT, BDS, shoulder dislocation, pain	No difference between groups in shoulder and elbow strength and motor function. No improvements in other outcome measures for both groups.	Very low
Zhang and Li 2014 [32]	Trunk acupuncture + rehabilitation training<br>vs<br>Rehabilitation training alone	16 weeks	Not reported	BI*	Acupuncture group (n=30)<br>Mean BI 22.50 (SD 6.79)<br><br>Rehabilitation group (n=29)<br>Mean BI 24.48 (SD 7.23)	BI, BBS	Acupuncture group demonstrated higher scores on BI and BBS compared to the rehabilitation group.	Very low

For peer review only

ARAT- Action Research Arm Test, AROM- active range of movement, AS- Ashworth Scale, BBS- Berg Balance Scale, BDS- Becks Depression Scale, BI*- Barthel Index (original version scored out of 100), BI^ - Barthel Index(revised version score out of 20), BS- Borg Scale, BWS- bodyweight supported, CNS- Canadian Neurological Scale, CRP- continuous relative phase, CSI- Caregiver Strain Index, EQ-5D-3L- EuroQoL questionnaire, FAC- Functional Ambulation Category, FAI- Frenchay Activities Index, FIM- Functional Independence Measure, FMA- Fugl-Meyer Assessment, FTHUE- Functional Test for the Hemiplegic Upper Extremity, GDS- Geriatric Depression Scale, GHQ-28- General Health Questionnaire-28, HADS- Hospital Anxiety and Depression Scale, LL- lower limb, MAS- Motor Assessment Scale, mAS- Modified Ashworth Scale, MCA- Motor Club Assessment, MI- Motricity Index, mMAS- Modified Motor Assessment Scale, MMSE- Mini-Mental State Examination, mNIHSS- Modified National Institutes of Health Stroke Scale, mRS- Modified Rankin Scale, MRC- Medical Research Council Scale for Muscle Strength, NEADL- Nottingham Extended Activities of Daily Living, NHP- Nottingham Health Profile, NIHSS- National Institutes of Health Stroke Scale, OT- occupational therapist, PASS- Postural Assessment Scale for Stroke Patients, PROM- passive range of movement, PT- physiotherapist, PTA- physiotherapy assistant, RMA- Rivermead Motor Assessment, RMI- Rivermead Mobility Index, RS- Rankin Scale, SIP- Stroke Impact Profile, SIS- Stroke Impact Scale, ST- speech therapist, STREAM- Stroke Rehabilitation Assessment of Movement, TCT- Trunk Control Test, THPT- Ten-Hole Peg Test, TUG- Timed Up and Go, UL- upper limb, WHS- Walking Handicap Scale, 6MWT- 6 minute walk test, 10MWT- 10 metre walk test

For peer review only

Supplementary Results- Outcomes Supported by Low or Very Low-Quality Evidence

Body function

Cardiorespiratory Function

Two studies explored participants' cardiorespiratory response to different types of treadmill gait training within the acute to early subacute phase post-stroke.[4,8] There was low-quality evidence that 2 weeks of robot-assisted bodyweight supported treadmill gait training delivered in the first 6 weeks post-stroke improved peak VO_2 compared to conventional gait training. [4] There was low-quality evidence that a 4-week course of bodyweight supported treadmill training delivered in the first 3 months post-stroke was not perceived to be more effortful than conventional gait training.[8]

Neurological Impairment

Three studies evaluated changes in neurological function. [6,25,27] In the acute to early subacute phase post-stroke, there was very low-quality evidence that there was no difference in an intensive or ordinary 2-week acute physical rehabilitation programme on reducing neurological impairment at 2 weeks and 6 months post-stroke.[6] In the acute to late subacute phase post-stroke, there was very low-quality evidence that a 3-month course of robot-assisted bodyweight supported treadmill gait training commenced within the first 6 weeks post-stroke was just as effective as conventional gait training on improving neurological function.[25] There was very low-quality evidence that an 8-week course of acupuncture provided in conjunction with rehabilitation during the subacute phase of stroke reduced neurological impairment compared to rehabilitation alone.[27]

Sensorimotor Function

Sixteen studies evaluated changes in sensorimotor function. Nine studies were performed in the acute to early subacute phase post-stroke, [3-5,7,8,10-13] five studies in the acute to late subacute phase post-stroke,[18,21,25,27,28] and two studies in the chronic phase post-stroke.[29,31] In the acute to early subacute phase post-stroke, there was low quality evidence from two studies that thermal stimulation in conjunction with standard rehabilitation resulted in improvements in lower limb sensorimotor function and strength when compared to standard rehabilitation alone.[5,10] Improvements in lower limb sensorimotor function were maintained at 12 months post-intervention. There was low quality evidence that 2 weeks of robot-assisted bodyweight supported treadmill gait training resulted in improvements in lower limb sensorimotor function but not strength compared to conventional gait training.[4] There was low quality evidence that there was no difference between: 4 weeks of robot-assisted treadmill gait training and conventional gait training on improving lower limb sensorimotor function;[13] wearing a cueing wristwatch and wearing a sham wristwatch for 3 hours per weekday for 3 weeks during rehabilitation on improving upper limb sensorimotor function and number of arm movements;[7] a 4-week course of bodyweight supported treadmill training and conventional overground gait training on improving lower limb strength;[8] and a 5-week course of additional upper limb therapy provided by a qualified physiotherapist or a physiotherapy assistant and standard physiotherapy on improving upper limb motor activity and grip strength.[11]

There was very low-quality evidence that a thrice weekly, 6-week course of electromyography (EMG) biofeedback combined with conventional physiotherapy had no

For peer review only

effect on improving lower limb active range of movement when compared to conventional physiotherapy alone.[3] There was very low-quality evidence that a 3-month course of acupuncture in conjunction with rehabilitation resulted in better upper and lower limb sensorimotor function when compared to rehabilitation alone.[12]

In the acute to late subacute phase post-stroke, there was very low quality evidence that a 6-week course of robotic tilt-table verticalisation that combines cyclic leg movements and FES and used in conjunction with standard physiotherapy resulted in better lower limb strength and sensorimotor function compared to physiotherapy-assisted verticalisation using a standard tilt-table and used in conjunction with standard physiotherapy.[18] There was very low-quality evidence that an 8-week course of acupuncture provided in conjunction with rehabilitation resulted in improvements in upper and lower limb sensorimotor function compared to rehabilitation alone.[27] There was very low-quality evidence that a 3-month course of nurse-led acupressure resulted in improvements in upper and lower limb motor function compared to routine care.[28] There was very low quality evidence that there was no difference between: a functionally-orientated and a sensorimotor integrative occupational therapy treatment approach delivered over 8 weeks on improving upper limb sensorimotor function;[21] and a 3-month course of robot-assisted bodyweight supported treadmill gait training and conventional gait training on improving lower limb power.[25]

In the chronic phase post-stroke, there was very low-quality evidence that a 6-week course of robot-assisted bodyweight supported treadmill gait training using slower treadmill speeds resulted in improvements in lower limb sensorimotor function compared to similar treadmill training using faster treadmill speeds.[29] There was very low-quality evidence that either an intensive therapist-driven UL protocol or an intensive robotic-driven UL protocol delivered thrice weekly for 6 weeks resulted in an improvement in shoulder and elbow sensorimotor function.[31]

Activity

Activities of Daily Living

Sixteen studies explored independence and ability to perform activities of daily living (ADLs). Nine studies were completed in the acute to early subacute phase,[2,6-8,10-13,15] six studies were completed in acute to late subacute phase[17,19-21,25,27,28] and one study was completed in the chronic phase.[32]

In the acute to early subacute phase, there was low quality evidence that a 6-week course of thermal stimulation used in conjunction with standard rehabilitation resulted in improvements in ADL independence 3 months post-stroke compared to standard rehabilitation alone, although improvements were not seen at 6 months post-stroke.[10]

There was low quality evidence that there was no difference between: regular physiotherapy and regular physiotherapy in conjunction with use of an Oswestry standing frame delivered over 14 consecutive weekdays in the first 3 months post-stroke on ADL independence;[2] wearing a cueing wristwatch and wearing a sham wristwatch for 3 hours per weekday for 3 weeks during rehabilitation on ADL independence;[7] a 4-week course of bodyweight supported treadmill training and conventional overground gait training on improving ADL independence;[8] a 5-week course of additional upper limb therapy provided by a qualified physiotherapist or a physiotherapy assistant and standard physiotherapy on improving ADL independence;[11] and 4 weeks of robot-assisted treadmill gait and conventional overground gait training on ADL independence.[13]

For peer review only

There was very low-quality evidence that there was no difference in an intensive or ordinary 2-week acute physical rehabilitation programme in improving ADL independence at 2 weeks and 6 months post-stroke.[6] There was very low-quality evidence that a 3-month course of acupuncture in conjunction with rehabilitation resulted in better ADL independence when compared to rehabilitation alone.[12] There was very low-quality evidence that providing additional physiotherapy in conjunction to regular rehabilitation in the first few weeks post-stroke resulted in improvements in ADL independence at 6 months post-stroke compared to regular rehabilitation alone.[15]

In the acute to late subacute phase, there was low quality evidence that a 6-month course of a staged physical rehabilitation programme resulted in greater improvements in ADL independence compared to usual care that did not involve formal rehabilitation.[17] There was very low-quality evidence that a monthly home-based physiotherapy programme delivered over 6 months resulted in improvements in ADL independence compared to standard care.[19,20] There was very low-quality evidence that there was no difference between a functionally orientated or a sensorimotor integrative occupational therapy treatment approach delivered over 8 weeks on ADL independence.[21] There was very low-quality evidence that a 3-month course of robot-assisted bodyweight supported treadmill gait training resulted in improvements in ADL independence compared to conventional gait training.[25] Improvements were only seen in the cohort of participants who demonstrated significant motor impairment. Improvements were maintained at the 2-year follow-up.[26] There was very low-quality evidence that an 8-week course of acupuncture provided in conjunction with rehabilitation during the subacute phase of stroke improved ADL independence compared to rehabilitation alone.[27] There was very low-quality evidence that a 3-month course of nurse-led acupressure resulted in improvements in ADL independence compared to routine care.[28]

In the chronic phase, there was very low-quality evidence that a 16-week course of trunk acupuncture combined with rehabilitation training resulted in greater improvements in ADL independence compared to rehabilitation training alone.[32]

Balance and Postural Control

Eight studies investigated balance and postural control. Four studies were completed in the acute to early subacute phase,[2,5,10,16] two studies were completed in the acute to late subacute phase[18,25] and two studies were completed in the chronic phase.[29,32]

In the acute to early subacute phase, there was low quality evidence that a 6-week course of thermal stimulation in conjunction with standard rehabilitation resulted in improvements in trunk postural control but not balance compared to standard rehabilitation alone.[5] In a separate study, there was low quality evidence that a 6-week course of thermal stimulation in conjunction with standard rehabilitation resulted in improvements in balance 3 months post-stroke compared to standard rehabilitation alone, although improvements were not seen at 6 months post-stroke.[10] There was low quality evidence that there was no difference between regular physiotherapy and regular physiotherapy in conjunction with use of an Oswestry standing frame delivered over 14 consecutive weekdays in the first 3 months post-stroke on trunk postural control.[2] There was low quality evidence that an 8-week course of physiotherapy involving early mobilisation combined with the Bobath approach resulted in improvements in balance when compared to physiotherapy just involving the Bobath approach.[16]

For peer review only

In the acute to late subacute phase, there was very low quality evidence that a 6-week course of robotic tilt-table verticalisation that combines cyclic leg movements and FES and used in conjunction with standard physiotherapy resulted in improved postural control during different activities compared to physiotherapy-assisted verticalisation using a standard tilt-table and used in conjunction with standard physiotherapy.[18] There was very low-quality evidence that a 3-month course of robot-assisted bodyweight supported treadmill gait training resulted in improvements in trunk control compared to conventional gait training.[25] Improvements were only seen in the cohort of participants who demonstrated significant motor impairment.

In the chronic phase, there was very low-quality evidence that a 6-week course of robot-assisted bodyweight supported treadmill gait training resulted in improvements in balance regardless if slower or faster treadmill training speeds were used.[29] There was very low-quality evidence that a 16-week course of trunk acupuncture combined with rehabilitation training resulted in greater improvements in balance compared to rehabilitation training alone.[32]

Gait

Eight studies investigated gait, which included gait ability and gait speed. Six studies were performed in the acute to early subacute phase,[3-5,8,10,13] one study was performed in the acute to late subacute phase[25] and one study was performed in the chronic phase.[29] In the acute to early subacute phase, there was low quality evidence from two studies that a 6-week course of thermal stimulation in conjunction with standard rehabilitation resulted in improvements in gait ability compared to standard rehabilitation alone.[5,10] There was low quality evidence that 4 weeks of robot-assisted treadmill gait training resulted in better gait ability than conventional gait training.[13] There was low quality evidence that there was no difference between: a 2-week course of robot-assisted bodyweight supported treadmill gait training and conventional gait training delivered in the first 6 weeks post-stroke on improving gait ability;[4] a 4-week course of bodyweight supported treadmill training and conventional overground gait training on improving gait ability;[8] and a thrice weekly, 6-week course of EMG biofeedback combined with conventional physiotherapy and conventional physiotherapy alone in improving gait speed.[3] In the acute to late subacute phase, there was very low-quality evidence that a 3-month course of robot-assisted bodyweight supported treadmill gait training resulted in improvements in gait ability compared to conventional gait training.[25] Improvements were only seen in the cohort of participants who demonstrated significant motor impairment. Improvements were maintained at the 2-year follow-up.[26]

In the chronic phase, there was very low-quality evidence that a 6-week course of robot-assisted bodyweight supported treadmill gait training using slower treadmill speeds resulted in improvements gait ability compared to similar treadmill training using faster treadmill speeds.[29]

General Physical Activity

Seven studies examined the effects of different interventions on improving general physical activity. Six studies were performed in the acute to early subacute phase[2,3,5,10,11,16] and one study was performed in the acute to late subacute phase.[25] In the acute to early subacute phase, there was low quality evidence from two studies that thermal stimulation in conjunction with standard rehabilitation resulted in improvements in physical activity when compared to standard rehabilitation alone.[5,10] Improvements were seen up until 3 months

post-intervention but disappeared at the 6-month follow-up. There was low quality evidence that an 8-week course of physiotherapy involving early mobilisation combined with the Bobath approach resulted in improvements in physical activity when compared to physiotherapy just involving the Bobath approach.[16] There was low quality evidence that there was no difference between: regular physiotherapy and regular physiotherapy in conjunction with use of an Oswestry standing frame delivered over 14 consecutive weekdays in the first 3 months post-stroke on physical activity;[2] and a 5-week course of additional upper limb therapy provided by a qualified physiotherapist or a physiotherapy assistant and standard physiotherapy on improving physical activity.[11] There was very low-quality evidence that there was no difference between a thrice weekly, 6-week course EMG biofeedback combined with conventional physiotherapy and conventional physiotherapy alone on improving physical activity.[3]
In the acute to late subacute phase, there was very low-quality evidence that a 3-month course of robot-assisted bodyweight supported treadmill gait training resulted in improvements in physical activity compared to conventional gait training.[25] Improvements were only seen in the cohort of participants who demonstrated significant motor impairment. Improvements were maintained at the 2-year follow-up.[26]

Upper Limb Function

Two studies investigated changes in upper limb function.[11,31] In the acute to early subacute phase, there was low quality evidence that a 5-week course of additional upper limb therapy provided by a qualified physiotherapist was no more effective at improving upper limb function than additional upper limb therapy provided by a physiotherapy assistant or to standard physiotherapy.[11] In the chronic phase, there was very low-quality evidence that there was no improvement in upper limb function with either an intensive therapist-driven UL protocol or an intensive robotic-driven UL protocol delivered thrice weekly for 6 weeks.[31]

Participation

Extended Activities of Daily Living

Four studies investigated the effect of different interventions on extended ADLs.[2,3,9,21] In the acute to early subacute phase, there was low quality evidence that there was no difference between: regular physiotherapy and regular physiotherapy in conjunction with use of an Oswestry standing frame delivered over 14 consecutive weekdays on ability to perform extended ADLs at 6 months post-stroke,[2] and an 8-week course of rehabilitation with the addition of a leg cycling machine compared to regular rehabilitation alone on extended ADLs 6 months post stroke.[9] There was very low-quality evidence that there was no difference between a thrice weekly, 6-week course of electromyography (EMG) biofeedback combined with conventional physiotherapy and conventional physiotherapy alone in improving performance in extended ADLs time.[3]
In the acute to late subacute phase, there was very low-quality evidence that there was no difference between a functionally orientated or a sensorimotor integrative occupational therapy treatment approach delivered over 8 weeks on the ability to prepare meals.[21]

Perceived Health Status

Two studies explored carers' and patients' perceived health status.[2,31] In the acute to early subacute phase, there was low quality evidence that there was no difference between regular physiotherapy and regular physiotherapy in conjunction with use of an Oswestry

For peer review only

standing frame delivered over 14 consecutive weekdays on carer's perceived health status at 12 weeks and 6 months post-stroke.[2] In the chronic phase, there was very low-quality evidence that there was no change in patient's perceived health status with the provision of either an intensive therapist-driven UL protocol or an intensive robotic-driven UL protocol delivered thrice weekly for 6 weeks.[31]

Quality of Life

There was very low-quality evidence that a monthly home-based physiotherapy programme delivered over 6 months resulted in an improvement in quality of life compared to standard care.[19]

Complications

Caregiver Burden

There was low quality evidence that there was no difference between regular physiotherapy and regular physiotherapy in conjunction with use of an Oswestry standing frame delivered over 14 consecutive weekdays in the first 3 months post-stroke on caregiver strain and psychological well-being at 12 weeks and 6 months post-stroke.[2]

Depression

Three studies explored changes in depression.[2,20,31] In the acute to early subacute phase, there was low quality evidence that there was no difference between regular physiotherapy and regular physiotherapy in conjunction with use of an Oswestry standing frame delivered over 14 consecutive weekdays on depression at 12 weeks and 6 months post-stroke.[2] In the acute to late subacute phase, there was very low-quality evidence that a monthly home-based physiotherapy programme delivered over 6 months resulted in a reduction in level of depression compared to standard care.[20] In the chronic phase, there was very low-quality evidence that there was no difference between an intensive therapist-driven UL protocol and an intensive robotic-driven UL protocol delivered thrice weekly for 6 weeks in reducing depression.[31]

Shoulder Pain/Dislocation

There was very low-quality evidence that either an intensive therapist-driven UL protocol or an intensive robotic-driven UL protocol delivered thrice weekly for 6 weeks had no effect on shoulder pain nor caused any shoulder dislocation when delivered to participants in the chronic phase post-stroke.[31]

Spasticity

Six studies explored the effect of different interventions on spasticity.[3,8,11,17,25,31] In the acute to early subacute phase, there was low quality evidence that there was no difference between: bodyweight supported treadmill training and conventional overground gait training delivered over 4 weeks on reducing lower limb spasticity;[8] and a 5-week course of additional upper limb therapy provided by a qualified physiotherapist or a physiotherapy assistant and standard physiotherapy on reducing upper limb spasticity.[11] There was very low-quality evidence that there was no reduction in spasticity with a 6-week course of conventional physiotherapy with or without EMG biofeedback.[3]

In the acute to late subacute phase, there was low quality evidence that a 6-month course of a staged physical rehabilitation programme resulted in a lower incidence of upper and

For peer review only

lower limb spasticity compared to usual care that did not involve formal rehabilitation.[17] There was very low-quality evidence that a 3-month course of either robot-assisted bodyweight supported treadmill training or conventional gait training had no effect on reducing lower limb spasticity.[25]

In the chronic phase, there was very low-quality evidence that there was no difference between an intensive therapist-driven UL protocol and an intensive robotic-driven UL protocol delivered thrice weekly for 6 weeks in reducing UL spasticity.[31]

Supplemental References

1 AVERT Trial Collaboration group. Efficacy and safety of very early mobilisation within 24 h of stroke onset (AVERT): a randomised controlled trial. *Lancet* 2015;386:46-55.
2 Bagley P, Hudson M, Forster A, et al. A randomized trial evaluation of the Oswestry Standing Frame for patients after stroke. *Clin Rehabil* 2005;19:354-364.
3 Bradley L, Hart BB, Mandana S, et al. Electromyographic biofeedback for gait training after stroke. *Clin Rehabil* 1998;12:11-22.
4 Chang WH, Kim MS, Huh JP, et al. Effects of Robot-Assisted Gait Training on Cardiopulmonary Fitness in Subacute Stroke Patients: A Randomized Controlled Study. *Neurorehab Neural Re* 2012;26(4):318-324.
5 Chen JC, Lin CH, Wei YC, et al. Facilitation of motor and balance recovery by thermal intervention for the paretic lower limb of acute stroke: a single-blind randomized clinical trial. *Clin Rehabil* 2011;25(9):823-832.
6 Di Lauro A, Pellegrino L, Savastano G, et al. A randomized trial on the efficacy of intensive rehabilitation in the acute phase of ischemic stroke. *J Neurol* 2003;250:1206-1208.
7 Fong K, Yang N, Chan M, et al. Combined effects of sensory cueing and limb activation on unilateral neglect in subacute left hemiplegic stroke patients: a randomized controlled pilot study. *Clin Rehabil* 2013;27(7):628-637.
8 Franceschini M, Carda S, Agosti M, et al. Walking After Stroke: What Does Treadmill Training With Body Weight Support Add to Overground Gait Training in Patients Early After Stroke? A Single-Blind, Randomized, Controlled Trial. *Stroke* 2009;40:3079-3085.
9 Katz-Leurer M, Carmeli E and Shochina M. The effect of early aerobic training on independence six months post stroke. *Clin Rehabil* 2003;17:735-741.
10 Liang CC, Hsieh TC, Lin CH, et al. Effectiveness of Thermal Stimulation for the Moderately to Severely Paretic Leg After Stroke: Serial Changes at One-Year Follow-Up. *Arch Phys Med Rehab* 2012;93:1903-1910.
11 Lincoln NB, Parry RH and Vass CD. Randomized, Controlled Trial to Evaluate Increased Intensity of Physiotherapy Treatment of Arm Function After Stroke. *Stroke* 1999;30:573-579.
12 Min M, Xin C, Yuefeng C, et al. Stage-oriented Comprehensive Acupuncture Treatment plus Rehabilitation Training for Apoplectic Hemiplegia. *J Tradit Chin Med* 2008;28(2):90-93.

For peer review only

13 Ochi M, Wada F, Saeki S, et al. Gait training in subacute non-ambulatory stroke patients using a full weight-bearing gait-assistance robot: A prospective, randomized, open, blinded-endpoint trial. *J Neurol Sci* 2015;353:130-136.
14 Rosewilliam S, Malhotra S, Roffe C, et al. Can surface neuromuscular electrical stimulation of the wrist and hand combined with routine therapy facilitate recovery of arm function in patients with stroke? *Arch Physl Med Rehab* 2012;93(10):1715-1721.
15 Sanchez-Sanchez ML, Belda-Lois JM, Horno SM, et al. Functional principal component analysis as a new methodology for the analysis of the impact of two rehabilitation protocols in functional recovery after stroke. *J Neuroeng Rehabil* 2014;11:134.
16 Tang Q, Tan L, Li B, et al. Early Sitting, Standing, and Walking in Conjunction With Contemporary Bobath Approach for Stroke Patients With Severe Motor Deficit. *Top Stroke Rehabil* 2014;21(2):120-127.
17 Bai YL, Hu YS, Wu Y, et al. Long-term three-stage rehabilitation intervention alleviates spasticity of the elbows, fingers, and plantar flexors and improves activities of daily living in ischemic stroke patients: a randomized, controlled trial. *Neuroreport* 2014;25:998-1005.
18 Calabro RS, Naro A, Russo M, et al. Do post-stroke patients benefit from robotic verticalization? A pilot-study focusing on a novel neurophysiological approach. *Restor Neurol and Neuros* 2015:33:671-681.
19 Chaiyawat P and Kulkantrakorn K. Effectiveness of home rehabilitation program for ischemic stroke upon disability and quality of life: A randomized controlled trial. *Clin Neurol Neuros* 2012:114:866-870.
20 Chaiyawat P and Kulkantrakorn K. Randomized controlled trial of home rehabilitation for patients with ischemic stroke: impact upon disability and elderly depression. *Psychogeriatrics* 2012;12:193-199.
21 Jongbloed L, Stacey S and Brighton C. Stroke Rehabilitation: Sensorimotor Integrative Treatment Versus Functional Treatment. *Am J Occup Ther* 1989;43(6):391-397.
22 Kwakkel G, Wagenaar RC, Twisk JWR, et al. Intensity of leg and arm training after primary middle-cerebral artery stroke: a randomised trial. *Lancet* 1999;354:191-196.
23 Kwakkel G, Kollen BJ and Wagenaar RC. Long term effects of intensity of upper and lower limb training after stroke: a randomised trial. *J Neurol Neurosur Ps* 2002;72:473-479.
24 Kwakkel G and Wagenaar RC. Effect of Duration of Upper- and Lower-Extremity Rehabilitation Sessions and Walking Speed on Recovery of Interlimb Coordination in Hemiplegic Gait. *Phys Ther* 2002;82(5):432-448.
25 Morone G, Bragoni M, Iosa M, De Angelis D, Venturiero V, Coiro P. Who May Benefit From Robotic- Assisted Gait Training? A Randomized Clinical Trial in Patients With Subacute Stroke. *Neurorehab Neural Re* 2011;25(7):636-644.
26 Morone G, Iosa M, Bragoni M, De Angelis D, Venturiero V, Coiro P. Who May Have Durable Benefit From Robotic Gait Training? A 2-Year Follow-Up Randomized Controlled Trial in Patients With Subacute Stroke. *Stroke* 2012;43:1140-1142.
27 Yang XH, Bing L and Ba-si O. Effect of acupuncture combined with rehabilitative training on neural functional recovery of stroke patients during recovery phase: a randomized controlled trial. *World J Acupunct Mox* 2014;24(4):17-23.

For peer review only

28 Yue S, Jian X and Wong T. Effects of a nurse-led acupressure programme for stroke patients in China. *J Clin Nurs* 2012;22:1182-1188.
29 Rodrigues TA, Goroso DG, Westgate PM, et al. Slow Versus Fast Robot-Assisted Locomotor Training After Severe Stroke. A Randomized Controlled Trial. *Am J Phys Med Rehab* 2017;96(Suppl):S165–S170.
30 Sackley CM, Walker MF, Burton CR, et al. An occupational therapy intervention for residents with stroke related disabilities in UK care homes (OTCH): cluster randomised controlled trial. *BMJ* 2015;350:h468.
31 Volpe BT, Lynch D, Rykman-Berland A, et al. Intensive Sensorimotor Arm Training Mediated by Therapist or Robot Improves Hemiparesis in Patients With Chronic Stroke. *Neurorehabi Neural Re* 2008;22:305-310.
32 Zhang HY and Li PF. Observation on efficacy of acupuncture combined with rehabilitation training for post-stroke balance disorders. *World J Acupunct Mox* 2014;24(2):25-29.

For peer review only

The effect of rehabilitation interventions on physical function and immobility-related complications in severe stroke- a systematic review

Journal:	*BMJ Open*
Manuscript ID	bmjopen-2019-033642.R1
Article Type:	Original research
Date Submitted by the Author:	02-Dec-2019

Complete List of Authors:	McGlinchey, Mark; King's College School, School of Population Health and Environmental Sciences; Guy's and Saint Thomas' Hospitals NHS Trust, Physiotherapy Department<br>James, Jimmy; Guy's and Saint Thomas' Hospitals NHS Trust, Physiotherapy Department<br>McKevitt, Christopher; King's College School, School of Population Health and Environmental Sciences<br>Douiri, Abdel; King's College School, School of Population Health and Environmental Sciences<br>Sackley, Catherine; King's College School, School of Population Health and Environmental Sciences

**Primary Subject Heading**:	Rehabilitation medicine
Secondary Subject Heading:	Neurology, Health services research
Keywords:	STROKE MEDICINE, PHYSIOTHERAPY, OCCUPATIONAL THERAPY, REHABILITATION MEDICINE

SCHOLARONE™
Manuscripts

The effect of rehabilitation interventions on physical function and immobility-related complications in severe stroke- a systematic review

Authors
Mark P McGlinchey [1,2], Jimmy James [2], Christopher McKevitt [1], Abdel Douiri [1], Catherine M Sackley [1]

1- School of Population Health and Environmental Sciences, Faculty of Life Sciences and Medicine, King's College London, London, SE1 1UL, England
2- Physiotherapy Department, St Thomas' Hospital, Guy's and St Thomas' NHS Foundation Trust, London, England

Corresponding Author
Mark P McGlinchey
School of Population Health and Environmental Sciences, Faculty of Life Sciences and Medicine, 6th floor Addison House, Guy's Campus, King's College London, London, SE1 1UL, England
Email address: mark.p.mcglinchey@kcl.ac.uk
Telephone: 0207 278 3456

Word Count- 4,953

For peer review only - http://bmjopen.bmj.com/site/about/guidelines.xhtml

Abstract

Objective: To evaluate the effectiveness of rehabilitation interventions on physical function and immobility-related complications in severe stroke.

Design: Systematic review of electronic databases (MEDLINE, EMBASE, CINAHL, AMED, PEDro, DORIS, CENTRAL) searched between January 1987 and November 2018.

Methods: The PRISMA statement guided the review. Randomised controlled trials comparing the effect of one type of rehabilitation intervention to another intervention, usual care or no intervention on physical function and immobility-related complications for patients with severe stroke were included. Studies that recruited participants with all levels of stroke severity were included only if subgroup analysis based on stroke severity was performed. Two reviewers screened search results, selected studies using pre-defined selection criteria, extracted data and assessed risk of bias for selected studies using piloted proformas. Marked heterogeneity prevented meta-analysis and a descriptive review was performed. The GRADE approach was used to assess evidence strength.

Results: 28 studies (n=2,677, mean age 72.7 years, 49.3% male) were included in the review. 24 studies were rated low or very low quality due to high risk of bias and small sample sizes. There was high quality evidence that very early mobilisation (i.e. mobilisation with 24 hours post-stroke) and occupational therapy in care homes were no more effective than usual care. There was moderate quality evidence supporting short-term benefits of wrist and finger neuromuscular electrical stimulation in improving wrist extensor and grip strength, additional upper limb training on improving upper limb function and additional lower limb training on improving upper limb function, independence in activities of daily living, gait speed, and gait independence.

Conclusions: There is a paucity of high-quality evidence to support the use of rehabilitation interventions to improve physical function and reduce immobility-related complications after severe stroke. Future research investigating more commonly used rehabilitation interventions, particularly to reduce post-stroke complications, is required.

PROSPERO registration number: CRD42017077737

Keywords: stroke rehabilitation, physiotherapy, occupational therapy

For peer review only - http://bmjopen.bmj.com/site/about/guidelines.xhtml

Strengths and Limitations of this Study

- This is the first systematic review to investigate rehabilitation interventions specifically to survivors of severe stroke
- The review included outcomes on physical function and immobility-related post-stroke complications, of which the latter contribute to high levels of caregiver burden and are less commonly reported outcomes in stroke rehabilitation research
- Marked heterogeneity of included studies prevented meta-analysis
- Most included studies were rated as low or very low-quality evidence due to unclear or high risk of bias as well as recruitment of very small samples

For peer review only

For peer review only - http://bmjopen.bmj.com/site/about/guidelines.xhtml

INTRODUCTION

Despite advances in stroke management over recent decades, stroke remains one of the most common causes of death and disability globally.|1,2| The mainstay of treating stroke is stroke rehabilitation, which aims to enable a person to achieve their optimal physical, cognitive, communicative, emotional and social level of function.|3-5| Rehabilitation of physical function comprises a large component of stroke rehabilitation programmes delivered by health-care professionals, such as physiotherapists and occupational therapists.|6-8| Whilst several systematic reviews support the use of rehabilitation interventions to improve aspects of physical function, such as motor function, balance, walking speed and activities of daily living,|9-11| it is not clear from these reviews if these interventions are effective for survivors of differing levels of stroke severity, particularly severe stroke.

Severe stroke can be understood as a stroke resulting in a significant amount of brain tissue damage and multiple neurological impairments, which leads to a significant loss of function and residual disability.|12| Dependent upon how it is measured, 14 - 31% of people who sustain a stroke globally are classified as having a severe stroke,|13-18| a cohort of the stroke population that experiences worse outcomes compared to survivors of less severe stroke.|19-30| In the initial hospitalisation phase post-stroke, they are more likely to develop acute medical complications, which are negatively associated with functional recovery.|19| Three month mortality can be as high as 40%, compared to just under 5% for those patients with mild stroke.|20-22| Survivors of severe stroke spend longer in hospital, resulting in increased hospital costs, and demonstrate slower and less functional recovery, resulting in greater dependency when they are discharged from hospital.|14,15,23,25| For those discharged from hospital, survivors of severe stroke are at least eight times more likely to be discharged to a nursing home.|25,26| Longer-term care costs, which mostly support survivors of severe stroke, represent 49% of total stroke care spending globally.|27| In the first year post severe stroke, mortality can be as high as 60%|20| and survivors of severe stroke also experience very high levels of immobility-related complications, such as falls, contracture, pain, and pressure sores.|28,29| Due to this residual disability, the physical assistance provided by caregivers to look after survivors of

For peer review only - http://bmjopen.bmj.com/site/about/guidelines.xhtml

severe stroke as well as the psychosocial and emotional impact of the stroke on caregivers result in high levels of caregiver burden.|30|

As there are a number of significant issues faced by survivors of severe stroke, rehabilitation of severe stroke should focus on addressing these poor outcomes, particularly reduced physical function and its associated complications. However, the extent to which rehabilitation can address these outcomes is not clear. A previous systematic review demonstrated positive benefits of inpatient stroke rehabilitation, such as reduced mortality and hospital length of stay, and uncertain benefit on improving functional recovery.|31| However, this review did not explore the effect of specific interventions delivered within inpatient rehabilitation on improving physical function or on reducing immobility-related complications. Most trials investigating the efficacy of rehabilitation interventions on physical function have either not recruited survivors of severe stroke or not reported results specifically for survivors of severe stroke.|9-11| Therefore, it is not known if research findings are applicable to survivors of severe stroke. It is not clear whether rehabilitation should focus more on functional restoration, which may be incomplete or not possible, or reducing immobility-related complications, which may lessen longer-term burden for caregivers of severe stroke survivors. Due to this lack of clarity, there is an urgent need to summarise evidence-based rehabilitation interventions designed to optimise physical function and reduce immobility-related complications for this cohort of the stroke population.

This systematic review aims to establish the effectiveness of rehabilitation interventions on physical function and immobility-related complications for survivors of severe stroke and identify areas for future rehabilitation research for these patients.

METHODS

The systematic review has been reported according to the Preferred Reporting Items for Systematic Reviews and Meta-analysis (PRISMA) statement (see Supplementary File 1).|32| The protocol for the systematic review has been published previously.|33|

For peer review only - http://bmjopen.bmj.com/site/about/guidelines.xhtml

Study design

The systematic review included randomised controlled trials (RCTs). The systematic review excluded quasi-experimental, correlational and descriptive study designs. Studies were selected according to the PICO (participant, intervention, comparator and outcome) format. The systematic review protocol provides full details of the PICO components|33| and a brief summary of the components is reported below. There were no deviations from the protocol PICO.

Participants

The review included studies of adult (≥ 18 years) stroke patients with severe stroke. Stroke severity was defined using a score on a validated and routinely used outcome measure (e.g. National Institutes of Health Stroke Scale (NIHSS), Functional Independence Measure (FIM), Barthel Index (BI). |34-36|

Interventions

The review included studies that involved the provision of rehabilitation interventions used to manage problems relating to physical function or immobility-related complications post-stroke. A rehabilitation intervention was defined as any non-surgical or non-pharmacological intervention used in current clinical practice as part of the usual rehabilitative care of stroke patients.

Comparators

The review included studies that had a comparator, which included any of the following: another type of rehabilitation intervention, usual care or no intervention. Usual care was defined as the rehabilitation that the patient would normally receive as part of undergoing stroke rehabilitation.

Outcomes

The review included studies that focused on the primary outcomes of physical function and post-stroke complications. As per the definition of function in the International Classification of Functioning, Disability and Health, physical function was assessed using measures of body function (e.g. Fugl-Meyer Assessment), activity (e.g. BI), and participation (e.g. Stroke

For peer review only - http://bmjopen.bmj.com/site/about/guidelines.xhtml

Impact Scale). |37, 38| An immobility-related complication was defined as any medical problem arising after a stroke because of immobility or reduced physical activity. |39|

Search strategy

Information sources

Electronic searches of the following databases were conducted: MEDLINE, EMBASE, Cumulative Index to Nursing and Allied Health Literature (CINAHL), Allied and Complementary Medicine Database (AMED), Physiotherapy Evidence Database (PEDro), Database of Research in Stroke (DORIS) and the Cochrane Central Register of Controlled Trials (CENTRAL). An example search strategy is shown in Supplementary File 2. Databases were searched from January 1987 to November 2018. The search timeframe was guided by a scoping review of the literature (demonstrating very few published RCTs before 2000) and a consideration to include studies reflecting current clinical practice. Ongoing studies were identified by searching the Stroke Trials Registry (www.strokecenter.org/trials/) and clinicaltrials.gov. These sources were searched from 2012 to 2018 as it was assumed that studies before these dates would have been completed and published. References from included studies were hand searched and any potentially relevant study was included for review. Forward citation checks of included studies were also performed. To avoid language or cultural bias, studies in any language or geographical location were included.

Data management and study selection

The results from the literature search were uploaded to a reference management programme (Refworks) and duplicate references were removed. A final list of non-duplicated references was generated by one author (MM). The titles and abstracts of the search results were screened independently by two review authors (MM and JJ) and full text articles were obtained for relevant studies. Full text articles were reviewed by the same two authors (MM and JJ) independently to determine if studies met the inclusion criteria using an inclusion/exclusion checklist previously piloted. Two review authors (MM and JJ) independently performed data extraction for all eligible articles using a data extraction proforma previously piloted. Any differences in opinion between the two authors at any

For peer review only - http://bmjopen.bmj.com/site/about/guidelines.xhtml

For peer review only

stage of the study selection and data extraction process were resolved by a third review author (CS).

Risk of bias and quality assessment

Risk of bias was assessed by two review authors independently (MM and JJ) using the Cochrane Collaboration tool for assessing the risk of bias across six main domains (sequence generation, allocation concealment, blinding, incomplete outcome data, selective outcome reporting, other bias) .|40| A risk of bias judgement of 'high', 'low' or 'unclear' was determined for each of these main domains. The strength of evidence was assessed using the Grading of Recommendations Assessment, Development and Evaluation (GRADE) approach.|40| The five criteria considered by the GRADE approach included risk of bias, inconsistencies between studies, indirectness, imprecision and publication bias. Studies were given a baseline rating of 'high' and downgraded if any of the five criteria were present. The quality of the evidence was ranked 'high', 'medium', 'low' or 'very low' by two review authors independently (MM and JJ). Any differences in opinion between the two authors at any stage of the study selection and data extraction process were resolved by a third reviewer (CS).

Data analysis

Due to the limited number of studies investigating each individual intervention and the marked heterogeneity of the selected studies, it was not appropriate to undertake a meta-analysis. Heterogeneity was seen in the rehabilitation interventions (type, dosage, method of delivery, timeframe completed post-stroke) as well as outcomes (type and timeframe completed post-stroke). Therefore, a descriptive review of results was performed. As there may be differences in recovery rates and outcomes according to the time post-stroke, studies were grouped into three timeframes post-stroke based on when participants were recruited to the study and when the study finished. These timeframes were the acute to early subacute stage (up to 3 months post-stroke), acute to late-subacute stage (up to 6 months post-stroke) and chronic stage (greater than 6 months post-stroke). These timeframes were chosen based on recommendations for the standardised measurement of

For peer review only - http://bmjopen.bmj.com/site/about/guidelines.xhtml

sensorimotor recovery in stroke trials.|41| Study findings were presented according to these three timeframes.

Patient and public involvement

There was no patient involvement in this study.

RESULTS

The initial literature review identified 7589 articles (Figure 1). After removing duplicates and screening titles and abstracts, 1083 full text articles were assessed for eligibility. 28 studies were included in the systematic review.|42-73| 2677 participants were recruited to these studies- mean participant age was 72.7 years, 49.3% were male and 87% of patients sustained a cerebral infarction. The main reasons for excluding studies were due to not recruiting participants with severe stroke, not providing results separately for participants with severe stroke or not providing sufficient information to determine if the participants had sustained a severe stroke. There was an excellent level of agreement between the two authors in selecting the included articles (Cohen's κ 0.93, percentage of agreement 97.7%).

The characteristics of the included studies are provided in Supplementary File 2 (Supplementary Tables 1 – 3, Supplemental References). 16 studies were completed within the acute-early subacute phase, eight studies were completed within the acute-late subacute phase and four studies were completed within the chronic phase post-stroke. 20 different interventions were evaluated across the 28 studies. The assessment of risk of bias for each study is presented in Figure 2.

Outcomes

60 measures of physical function and immobility-related post-stroke complications were identified across the studies. The measures were classified as measures of body function (n=18), activity (n=26), participation (n=8) and post-stroke complications (n=8). These measures were grouped together as 16 different outcomes. An overview of these measures and outcomes have been included in Supplementary File 2 (Supplementary Table 4).

For peer review only - http://bmjopen.bmj.com/site/about/guidelines.xhtml

For each outcome, there was usually only one study investigating the effectiveness of a specific rehabilitation intervention in each time frame post-stroke. Most of these studies were rated as providing very low or low-quality evidence for these outcomes (see Supplementary File 2). Outcomes which were supported by studies providing moderate or high quality of evidence are reported in this section. Outcomes which were supported by studies providing low or very low quality of evidence are reported in Supplementary File 2 (Supplementary Results, Supplemental References).

Body function

Sensorimotor Function

Seventeen studies evaluated changes in sensorimotor function. Ten studies were completed in the acute to early subacute phase post-stroke,| 44-46,48,49,51-55| five studies were completed in the acute to late subacute phase post-stroke|59,62,66,68,69| and two studies were completed in the chronic phase post-stroke.|70,72| The most frequently used outcome measures of sensorimotor function were the Fugl-Meyer Assessment, used in 11 studies, |45,46,48,51,53,54,59,68-70,72| and the MRC scale for muscle strength, used in 5 studies. |46,51,52,59,72|

In the acute to early subacute phase post-stroke, there was moderate quality evidence from one study that a 6-week course of neuromuscular electrical stimulation (NMES) applied to the wrist and finger extensors in conjunction with usual therapy resulted in no improvement in wrist active movement compared to usual therapy.|55| Wrist strength and grip strength improved in the NMES group during the treatment period although these improvements were not evident at the 9-month follow-up.

Activity

Activities of Daily Living

Twenty studies explored independence and ability to perform activities of daily living (ADLs). Eleven studies were completed in the acute to early subacute phase,|42,43,47-49,51-56| seven studies were completed in acute to late subacute phase|58,60-63,66-69| and two studies were completed in the chronic phase.|71,73| Eighteen studies used the Barthel Index as the main outcome measure to assess independence in ADLs. |43,47,49,51-

For peer review only - http://bmjopen.bmj.com/site/about/guidelines.xhtml

53,55,56,58,60-63,66-69,71,73|Four studies used the Modified Rankin Scale |42,49,60,61| and three studies used the Functional Independence Measure. |48,50,54|

In the acute to early subacute phase, there was high quality evidence that frequent, very early mobilisation (median of 6.5 times per day) commencing within 24 hours post-stroke did not result in more patients being less dependent in ADLs at 3 months post-stroke compared to usual care, which traditionally started more than 24 hours post-stroke and averaged 3 times per day.|42| However, caution is required with interpreting this finding as the sub-group analysis of patients with severe stroke was not powered for this outcome. There was moderate quality evidence that a 6-week course of NMES applied to the wrist and finger extensors in conjunction with usual therapy resulted in no difference in ADL independence compared to usual care.|55|

In the acute to late subacute phase, there was moderate quality evidence that additional lower limb (LL) therapy in conjunction with regular physical rehabilitation performed in the first 20 weeks post-stroke improved ADL independence whilst the intervention was being delivered when compared to regular physical rehabilitation alone.|63| However, these improvements were not seen 6 months post-stroke.

In the chronic phase, there was high quality evidence that a 3-month occupational therapy (OT) intervention provided to residents in care homes resulted in no difference in ADL independence compared to usual care.|71| Similar caution is required with interpreting this finding as the sub-group analysis of patients who were severely or very severely disabled was not powered for this outcome.

Gait

Nine studies investigated gait, which included gait ability and gait speed. Six studies were performed in the acute to early subacute phase,|44-46,49,51,54| two studies were performed in the acute to late subacute phase|63,66| and one study was performed in the chronic phase.|70| The Functional Ambulation Classification was used in eight studies, making it the most frequently used outcome measure of gait ability.|45,46,49,51,54,63,66,70| The 10-metre walk test was used in five studies, making it the most frequently used outcome measure of gait speed.|44,49,54,66,70|

Only one study demonstrated moderate quality evidence. |63| In the acute to late subacute phase, additional LL therapy in conjunction with regular physical rehabilitation performed in

For peer review only - http://bmjopen.bmj.com/site/about/guidelines.xhtml

the first 20 weeks post-stroke improved gait ability and speed when compared to regular physical rehabilitation alone.|| However, these improvements were not seen 6 months post-stroke.

General Physical Activity

Eight studies examined the effects of different interventions on improving general physical activity. Six studies were performed in the acute to early subacute phase,|43,44,46,51,52,57| one study was performed in the acute to late subacute phase|66| and one study was performed in the chronic phase.|71| General physical activity was defined as a composite of multiple physical tasks completed within one assessment, such as upper limb (UL) or LL function, transfers, gait and balance. Outcome measures used to assess general physical activity included the Rivermead Mobility Index, Rivermead Mobility Assessment and Motor Assessment Scale. Only one study demonstrated high quality evidence.|71| In the chronic phase, a 3-month OT intervention provided to residents in care homes resulted in no difference in physical activity compared to usual care.

Upper Limb Function

Four studies investigated changes in UL function,|52,55,63,72| of which two provided moderate quality evidence.|55,63| In the acute to early subacute phase, a 6-week course of NMES applied to the wrist and finger extensors in conjunction with usual therapy resulted in no difference in UL function compared to usual care.|55| In the acute to late subacute phase, additional UL or LL therapy in conjunction with regular physical rehabilitation performed in the first 20 weeks post-stroke improved UL function 6 months post-stroke when compared to regular rehabilitation.|63|

Participation

Instrumental Activities of Daily Living

Five studies investigated the effect of different interventions on instrumental ADLs,|43,44,50,62,63| of which one provided moderate quality evidence. Instrumental ADLs are those activities that enable an individual to live independently within their

For peer review only - http://bmjopen.bmj.com/site/about/guidelines.xhtml

community. In the acute to late subacute phase, additional UL or LL therapy in conjunction with regular physical rehabilitation performed in the first 20 weeks post-stroke improved performance in instrumental ADLs 6 months post-stroke when compared to regular rehabilitation.|63|

Quality of Life

Three studies examined quality of life,|60,63,71| of which two were moderate or high quality.|63,71| In the acute to late subacute phase, there was moderate quality evidence that there was no benefit of additional UL or LL therapy to regular physical rehabilitation performed in the first 20 weeks post-stroke on improving quality of life 6 months post-stroke.|63| In the chronic phase, there was high quality evidence that a 3-month OT intervention provided to residents in care homes resulted in no difference in quality of life compared to usual care.|71|

Complications

Depression

Four studies explored changes in depression,|43,61,71,72| of which one was high quality.|71| In the chronic phase, a 3-month OT intervention provided to residents in care homes resulted in no difference in depression compared to usual care.|71|

Mortality

One study investigated the effect of very early mobilisation on mortality.|42| There was high quality evidence that frequent, higher dose, very early mobilisation commencing within 24 hours post-stroke did not result in more patients dying at 3 months when compared to usual care, which traditionally started more than 24 hours post-stroke.

Other Outcomes

There was low quality of evidence for cardiorespiratory function (2 studies)|45,49| and caregiver burden (1 study).|43| There was very low to low quality of evidence for neurological impairment (3 studies),|47,66,68| balance and postural control (8 studies),|43,46,51,57,59,66,70,73| perceived health status (2 studies),|43,72| shoulder

For peer review only - http://bmjopen.bmj.com/site/about/guidelines.xhtml

pain and dislocation (1 study),|72| and spasticity (6 studies).|44,49,52,58,66,72| Further details of these outcome and studies are included in Supplementary File 2.

DISCUSSION

Main Findings

Although 28 RCTs investigating 20 different rehabilitation interventions were identified in this review, there was a paucity of high-quality evidence to support the use of these interventions to improve physical function and reduce immobility-related complications after severe stroke. Most studies were rated as low or very low-quality evidence due to unclear or high risk of bias as well as recruitment of very small samples (refer to Supplementary Table 1). However, compared to data from national (United Kingdom) and global estimates of stroke incidence and prevalence, participants recruited to these studies were similar in terms of stroke type and gender but slightly younger (median age of stroke in the United Kingdom is 77 years). |1,2,18|Therefore, participants were generally representative of the wider stroke population.

Physical Function

Two large, multi-centre studies provided high quality evidence that their respective treatment interventions were no more effective at improving different aspect of physical function than usual care.|42,71| However, patients with severe stroke or severe disability post-stroke comprised a smaller sample within these larger trials. Analyses of data from these sub-groups may not be powered to detect changes between the treatment and usual care interventions and therefore caution is required in interpreting the studies' findings.
In AVERT (A Very Early Rehabilitation Trial),|42| very early and frequent mobilisation commencing within 24 hours post-stroke did not result in more patients being less dependent in ADLs 3 months post-stroke compared to usual care, which traditionally started more than 24 hours post-stroke. Although the data seemed to favour usual care practice for patients with severe stroke, this finding did not achieve statistical significance. It could be argued that patients with severe stroke may be less likely to tolerate very early and intensive therapy in the first few days after stroke due to fatigue and reduced exercises tolerance. |74|This would suggest that mobilising patients less intensively after 24 hours

For peer review only - http://bmjopen.bmj.com/site/about/guidelines.xhtml

may be more beneficial at improving functional recovery than very early and frequent mobilisation. However, this finding was not seen in AVERT.

In the OT in care home trial,|71| a 3-month, goal-orientated OT intervention for stroke survivors living in care homes did not result in improved ADL ability or quality of life up to 1-year post-intervention. The authors hypothesised that the lack of treatment effect may have been due to the care home residents' disability severity, which may have limited their engagement in therapy. However, a content analysis of the OT intervention by the research team revealed that the mean number of OT visits over the period was 5.1 (SD 3.0), the median session time was 30 minutes (IQR 15-60 minutes) and only 15% of OT time was used to provide ADL and mobility training. Although session length and duration were dependent upon the care home resident's ability to engage, it is possible that a more frequent OT intervention that focussed more on ADL and mobility training may have resulted in different findings.

Two additional studies provided moderate quality evidence that their respective treatment interventions were effective at improving different aspects of physical function. In both studies, improvements were seen in different aspects of physical function that were specifically trained with the treatment intervention. Kwakkel et al. demonstrated that, compared to usual care, a 20-week course of additional upper limb therapy resulted in improvements in upper limb function and additional lower limb training resulted in improvements in upper limb function, independence in ADLs, gait speed and gait independence. |63|However, these improvements were not maintained after 6 months post-stroke once the additional therapy had discontinued.|64| Rosewilliam et al. demonstrated that the addition of wrist and finger neuromuscular electrical stimulation to usual therapy care resulted in improvements in wrist extensor and grip strength but no difference in upper limb function nor independence in ADLs. |55|As the electrical stimulation provided to patients was limited to cyclical movements of the wrist and did not involves multiple limb segments, it seems reasonable that upper limb function and independence in ADLs, which were not specifically trained for with the neuromuscular stimulation, did not improve.

Immobility-Related Complications

For peer review only - http://bmjopen.bmj.com/site/about/guidelines.xhtml

As demonstrated in Supplementary Table 2, there were relatively fewer complication outcomes investigated across all studies compared to physical function outcomes. This observation may reflect that the primary focus of stroke rehabilitation is to optimise functional recovery. |3-5|Therefore, the primary focus of stroke rehabilitation research investigating the effectiveness of rehabilitation interventions may be on improving functional recovery post-stroke rather than reducing immobility-related complications. Only two high-quality studies investigated the effectiveness of their respective interventions at reducing immobility-related complications. In AVERT, very early and frequent mobilisation commencing within 24 hours post-stroke did not result in more patients dying at 3 months post-stroke compared to usual care. |42|Whilst this finding is obviously positive, very early and frequent mobilisation did not result in less patient dependency as reported earlier in the discussion. Therefore, the optimal time and frequency to commence the mobilisation of patients with severe stroke is not clear.

In the OT in care home trial,|71| a 3-month, goal-orientated OT intervention for stroke survivors living in care homes did not result in reduced depression up to 1-year post-intervention. Whilst post-stroke depression has a multi-factorial cause, it has been reported that mental distress associated with residual disability may contribute to the development of post-stroke depression.|75| Therefore, reductions in residual disability may alleviate depressive symptoms post-stroke. As the OT intervention did not result in improved ADL ability, it is possible that depression did not significantly change due to the lack of improvement in ADL ability.

Implications for Practice and Research

In light of these findings, it may be necessary to re-evaluate the design of future trials investigating rehabilitation interventions in severe stroke. As it is not known if survivors of severe stroke respond to interventions in the same ways as survivors of milder stroke, there may be a need for more proof of concept studies to understand the mechanisms of recovery in severe stroke more fully. The high number of small, low-quality, single-centre RCTs investigating a broad range of interventions may suggest that larger, high-quality multi-centre RCTs investigating fewer interventions are warranted. However, outcome evaluations alone are insufficient to understand why certain interventions do or do not work. It is recommended that evaluations of complex interventions, such as stroke

For peer review only - http://bmjopen.bmj.com/site/about/guidelines.xhtml

rehabilitation, use process evaluations alongside outcome evaluations.|76| Process evaluations enable an understanding of how to implement an intervention as well as how participants respond to and interact with the intervention. Therefore, future trials should be guided by more proof of concept research and involve both outcome and process evaluations.

In this review, the most frequently investigated outcomes were functional tasks, such as ADLs and gait ability. However, Pereira et al. has suggested that individuals with severe stroke are likely to make limited functional improvement with inpatient rehabilitation in the their review of rehabilitation after severe stroke.|31| They also advocated more focus on discharge planning and reducing post-stroke complications during inpatient rehabilitation for patients with severe stroke. Whilst the extent to which patients can improve functionally after severe stroke is not clear, there is merit in further exploring the effect of rehabilitation in the prevention and management of post-stroke complications in severe stroke. Sackley et al. investigated the prevalence of immobility-related complications in the first year after severely disabling stroke and found a very high prevalence of falls, contractures, pain and pressure sores.|28| However, with the exception of spasticity, there was very little focus on the prevention or management of post-stroke complications in the studies selected for our systematic review. In addition to a lack of focus on immobility-related complications, only one study explored caregiver burden, known to be very high amongst carers looking after survivors of severe stroke.|30| Future research in the rehabilitation of severe stroke should therefore focus more on the effectiveness of rehabilitation interventions in the prevention and management of immobility-related complications in severe stroke.

This review identified several studies investigating technological interventions, such as treadmill training and robot-assistive devices, and more novel interventions, such as thermal stimulation. However, it is not clear how commonly used these interventions are in clinical practice. Additionally, there were no trials studies of interventions commonly used with survivors of severe stroke, such as positioning, sitting balance and seating.|77| This mismatch between available research evidence, which may not reflect current practice, and clinical practice, which may have limited research evidence to support its use, may present a dilemma for therapists, who are expected to base healthcare decisions on the best available and relevant evidence.|78| Therefore, future research is required to understand what interventions are currently being used in clinical practice. Knowledge of currently used

For peer review only - http://bmjopen.bmj.com/site/about/guidelines.xhtml

rehabilitation interventions may guide future trials investigating their efficacy in improving physical function and reducing immobility-related post-stroke complications.

Strengths and Limitations

In terms of strengths, this is the first systematic review to investigate rehabilitation interventions specifically to survivors of severe stroke, who tend to be underrepresented in stroke rehabilitation research, and the identification of topics for future rehabilitation research will hopefully guide much needed research for this cohort of the stroke population. As well, the outcomes of the review focussed on not just physical function but immobility-related post-stroke complications, which are known to be higher in the severe stroke population and contribute to high levels of caregiver burden.|28-30| In terms of limitations, it has been reported that the defining severe stroke is difficult due to different criteria used to classify severity.|79| The use of objective scores on validated outcome measures to classify stroke severity in our systematic review was deemed necessary to ensure that participants had actually sustained a severe stroke. In our review, the BI was the most commonly used measure to classify stroke severity, reported in 17 out of 28 studies. Using a pre-specified score on the BI to classify severe stroke (≤9/20 or ≤45/100)|33| enabled the identification of patients with severely disabling stroke. However, the use of an alternative measure of stroke severity, such as the NIHSS, may have resulted in the inclusion of a study with participants with a slightly different clinical presentation than participants measured with the BI. Alternatively, we may have excluded studies that used a different scoring system to classify stroke severity. However, these studies were discussed in detail amongst three review authors to determine suitability for inclusion and therefore it is likely that the number of relevant studies excluded from the review was minimal. Another limitation is the use of data from subgroups within larger clinical trials. As subgroup analyses may not be powered to detect changes between groups, caution is required in the interpretation of findings from these trials.

CONCLUSION

There was a paucity of high-quality evidence to support the use of rehabilitation interventions to improve physical function and reduced immobility-related complications after severe stroke. Two high quality studies suggested that very early mobilisation and

For peer review only - http://bmjopen.bmj.com/site/about/guidelines.xhtml

occupational therapy in care homes were no more effective than usual care. One moderate quality study supported wrist and finger neuromuscular electrical stimulation in improving wrist extensor and grip strength. One moderate quality study supported that use of additional upper limb training on improving upper limb function and additional lower limb training on improving upper limb function, independence in ADLs, gait speed and gait independence. Future research should be guided by more proof of concept studies and involve outcome and process evaluations to more fully understand the impact of different interventions on patients with severe stroke. Future research should investigate the effect of more clinically used interventions, such as positioning, sitting balance and seating. Future research should also investigate the effect of interventions on post-stroke complications known to be high after severe stroke, such as contracture, pressure sores and caregiver burden.

Authors' Contributions

MM is the guarantor of the review. MM, CS and CM were involved in the design of the protocol and systematic review. MM conducted scoping searches. MM and JJ piloted the inclusion/exclusion form. MM piloted the data extraction form. MM was the first reviewer and JJ was the second reviewer for the systematic review. AD provided statistical support for the systematic review. MM drafted the manuscript. All authors read and approved the final manuscript.

Competing Interests

None declared.

Funding

This project forms part of MM's PhD which is funded by The Dunhill Medical Trust [grant number RT62/0116]. The funder has had no input on the design of the protocol and will have no input on the analysis and interpretation of the results of the systematic review, or publication of the systematic review.

Patient consent

Not required.

For peer review only - http://bmjopen.bmj.com/site/about/guidelines.xhtml

For peer review only

Data availability statement

All data relevant to the study are included in the article or uploaded as supplementary information.

For peer review only

REFERENCES

1 GBD 2016 Causes of Death Collaborators. Global, regional, and national age-sex specific mortality for 264 causes of death, 1980–2016: a systematic analysis for the Global Burden of Disease Study 2016. *Lancet* 2017;390:1151–1210.

2 GBD 2016 DALYs and HALE Collaborators. Global, regional, and national disability-adjusted life-years (DALYs) for 333 diseases and injuries and healthy life expectancy (HALE) for 195 countries and territories, 1990–2016: a systematic analysis for the Global Burden of Disease Study 2016. *Lancet* 2017;390:1260–1344.

3 National Institute for Health and Care Excellence. Stroke rehabilitation in adults, https://www.nice.org.uk/guidance/cg162 (2013, accessed 1 August 2019).

4 Hebert D, Lindsay MP, McIntyre A, et al. Canadian stroke best practice recommendations: Stroke rehabilitation practice guidelines, update 2015. *Int J Stroke* 2016;11(4):459-484.

5 Langhorne P, Bernhardt J and Kwakkel G. Stroke rehabilitation. *Lancet* 2011;377:1693–1702.

6 Bode R, Heinemann A, Semik P, et al. Patterns of Therapy Activity Across Length of Stay and Impairment Levels: Peering Inside the “Black Box” of Inpatient Stroke Rehabilitation. *Arch Phys Med Rehab* 2004;85:1901-1908.

7 De Wit L, Putman K, Lincoln N, et al. Stroke rehabilitation in Europe- what do physiotherapists and occupational therapists actually do? *Stroke* 2006;37:1483-1489.

8 Lang C, MacDonald J, Reisman D, et al. Observation of Amounts of Movement Practice Provided During Stroke Rehabilitation. *Arch Phys Med Rehab* 2009;90:1692-1698.

9 Veerbeek JM, van Wegen E, van Peppen R, et al. What is the evidence for physical therapy poststroke? A systematic review and meta-analysis. *PLoS One* 2014;9(2)e87987.

For peer review only - http://bmjopen.bmj.com/site/about/guidelines.xhtml

10 Pollock A, Baer G, Campbell P, et al. Physical rehabilitation approaches for the recovery of function and mobility following stroke. *Cochrane DB Syst Rev* 2014. doi: 10.1002/14651858.CD001920.pub3
11 Legg LA, Lewis SR, Schofield-Robinson OJ, et al. Occupational therapy for adults with problems in activities of daily living after stroke. *Cochrane DB Syst Rev* 2017, doi:10.1002/14651858.CD003585.pub3
12 Teasell R, Pereira S and Cotoi A. The Rehabilitation of Severe Stroke, http://www.ebrsr.com/sites/default/files/v18-SREBR-CH22-NET-1.pdf (2018, accessed 1 August 2019).
13 Kelly-Hayes M, Wolf PA, Kennel WB, et al. Factors influencing survival and need for institutionalization following stroke: the Framingham study. *Arch Phys Med Rehab* 1988;69:415-418.
14 Jorgensen HS, Nakayama H, Raaschou HO, et al. Outcome and time course of recovery in stroke. Part I: Outcome. The Copenhagen stroke study. *Arch Phys Med Rehab* 1995;76:399-405.
15 Jorgensen HS, Nakayama H, Rancho HO, et al. Outcome and time course of recovery in stroke. Part II: Time Course of Recovery. The Copenhagen stroke study. *Arch Phys Med Rehab* 1995;76:406-412.
16 Islam MS, Anderson CS, Hankey GJ, et al. Trends in incidence and outcome of stroke in Perth, Western Australia during 1989 to 2001. The Perth community stroke study. *Stroke* 2008;39:776-782.
17 Luengo-Fernandez R, Paul N, Gray A, et al. Population-Based Study of Disability and Institutionalization After Transient Ischemic Attack and Stroke 10-Year Results of the Oxford Vascular Study. *Stroke* 2013;44:2854-2861.
18 McGlinchey MP, Paley L, Hoffman A, et al. Physiotherapy provision to hospitalised stroke patients: analysis from the UK Sentinel Stroke National Audit Programme. *Eur Stroke J* 2019;4(1):75-84
19 Kim BR, Lee, J, Sohn MK, Kim DY, Lee SG, Shin YI et al. Risk factors and functional impact of medical complications in stroke. *Ann Rehabil Med* 2017;41(5):753-760.
20 Bamford J, Santrock P, Dennis M, et al. Classification and natural history of clinically identifiable subtypes of cerebral infarction. *Lancet* 1991;337:1521-1526.
21 Heuschmann P, Kolominsky-Rabas P, Misselwitz B, et al. Predictors of In-Hospital Mortality and Attributable Risks of Death After Ischemic Stroke: The German Stroke Registers Study Group. *Arch Intern Med* 2004;1761-1768.
22 Bhaskar S, Stanwell P, Bivard A, et al. The influence of initial stroke severity on mortality, overall functional outcome and in-hospital placement at 90 days following acute ischemic stroke: A tertiary hospital stroke register study. *Neurol India* 2017;65(6):1252-1259.
23 Xu XM, Vestesson E, Paley L, et al. The economic burden of stroke care in England, Wales and Northern Ireland: Using a national stroke register to estimate and report patient-level health economic outcomes in stroke. *Eur Stroke J* 2018;3(1):82-91.
24 Douiri A, Grace J, Sarker SJ, et al. Patient-specific prediction of functional recovery after stroke. *Int J Stroke* 2017;12(5):539-548
25 Saxena SK, Ng TP, Yong D, et al. Total direct cost, length of hospital stay, institutional discharges and their determinants from rehabilitation settings in stroke patients. *Acta Neurol Scand* 2006;114:307-314.

For peer review only - http://bmjopen.bmj.com/site/about/guidelines.xhtml

26 McKenna K, Tooth L, Strong J, et al. Predicting discharge outcomes for stroke patients in Australia. *Am J Phys Med Rehab* 2002;81:47-56.
27 Evers SM, Struijs JN, Ament AJ, et al. International comparison of stroke cost studies. *Stroke* 2004;35:1209–1215
28 Sackley C, Brittle N, Patel S, et al. The prevalence of joint contractures, pressure sores, painful shoulder, other pain, falls and depression in the year after a severely disabling stroke. *Stroke* 2008;39:3329-3334.
29 Langhorne P, Stott DJ, Robertson L, et al. Medical Complications After Stroke: A Multicentre Study. *Stroke* 2000;31:1223-1229.
30 Rigby H, Gubitz G and Phillips S. A systematic review of caregiver burden following stroke. *Int J Stroke* 2009;4:285-292.
31 Pereira S, Graham JR, Shahabaz A, et al. Rehabilitation of individuals with severe stroke: synthesis of best evidence and challenges in implementation. *Top Stroke Rehabil* 2012;19(2),122-131.
32 Moher D, Liberati A, Tetzlaff J, et al. Preferred reporting Items for systematic reviews and meta-Analyses: The PRISMA statement. *PLoS Med* 2009;6(7):e1000097.
33 McGlinchey MP, James J, McKevitt C, et al. The effect of rehabilitation interventions of physical function and immobility-related complications in severe stroke- protocol for a systematic review. *Syst Rev* 2018;7:197
34 Brott TG, Adams HP, Olinger CP, Marler JR, Barsan WG, Biller J, et al. Measurements of acute cerebral infarction: a clinical examination scale. *Stroke* 1989;20:864–70.
35 Keith RA, Granger CV, Hamilton BB, et al. The functional independence measure: a new tool for rehabilitation. *Adv Clin Rehabil* 1987;1:6 –18. 38.
36 Mahoney FI, Barthel DW. Functional evaluation: the Barthel index. *Md State Med J* 1965;14:61 –5
37 Fugl-Meyer AR, Jaasko L, Leyman I, et al. The post-stroke hemiplegic patient. 1. A method for evaluation of physical performance. *Scand J Rehabil Med* 1975;975:13 –31
38 Duncan PW, Bode RK, Min Lai S, et al. Rasch analysis of a new stroke specific outcome scale: the Stroke Impact Scale. *Arch Phys Med Rehabil* 2003; 84:950–963.
39 Bhalla A, Birns J. Management of post-stroke complications. Switzerland: Springer International 2015: 1-6.
40 Higgins JPT and Green S. Cochrane handbook for systematic reviews of interventions Version 5.1.0. The Cochrane Collaboration, http://handbook-5-1.cochrane.org/ (2011, Accessed 1 August 2019).
41 Kwakkel G, Lannin NA, Borschmann K, et al. Standardised measurement of sensorimotor recovery in stroke trials: consensus-based core recommendations from the Stroke Recovery and Rehabilitation Roundtable. *Int J Stroke* 2017;12:451-461.
42 AVERT Trial Collaboration group. Efficacy and safety of very early mobilisation within 24 h of stroke onset (AVERT): a randomised controlled trial. *Lancet* 2015;386:46-55.
43 Bagley P, Hudson M, Forster A, et al. A randomized trial evaluation of the Oswestry Standing Frame for patients after stroke. *Clin Rehabil* 2005;19:354-364.
44 Bradley L, Hart BB, Mandana S, et al. Electromyographic biofeedback for gait training after stroke. *Clin Rehabil* 1998;12:11-22.
45 Chang WH, Kim MS, Huh JP, et al. Effects of Robot-Assisted Gait Training on Cardiopulmonary Fitness in Subacute Stroke Patients: A Randomized Controlled Study. *Neurorehab Neural Re* 2012;26(4):318-324.

For peer review only - http://bmjopen.bmj.com/site/about/guidelines.xhtml

46 Chen JC, Lin CH, Wei YC, et al. Facilitation of motor and balance recovery by thermal intervention for the paretic lower limb of acute stroke: a single-blind randomized clinical trial. *Clin Rehabil* 2011;25(9):823-832.
47 Di Lauro A, Pellegrino L, Savastano G, et al. A randomized trial on the efficacy of intensive rehabilitation in the acute phase of ischemic stroke. *J Neurol* 2003;250:1206-1208.
48 Fong K, Yang N, Chan M, et al. Combined effects of sensory cueing and limb activation on unilateral neglect in subacute left hemiplegic stroke patients: a randomized controlled pilot study. *Clin Rehabil* 2013;27(7):628-637.
49 Franceschini M, Carda S, Agosti M, et al. Walking After Stroke: What Does Treadmill Training With Body Weight Support Add to Overground Gait Training in Patients Early After Stroke? A Single-Blind, Randomized, Controlled Trial. *Stroke* 2009;40:3079-3085.
50 Katz-Leurer M, Carmeli E and Shochina M. The effect of early aerobic training on independence six months post stroke. *Clin Rehabil* 2003;17:735-741.
51 Liang CC, Hsieh TC, Lin CH, et al. Effectiveness of Thermal Stimulation for the Moderately to Severely Paretic Leg After Stroke: Serial Changes at One-Year Follow-Up. *Arch Phys Med Rehab* 2012;93:1903-1910.
52 Lincoln NB, Parry RH and Vass CD. Randomized, Controlled Trial to Evaluate Increased Intensity of Physiotherapy Treatment of Arm Function After Stroke. *Stroke* 1999;30:573-579.
53 Min M, Xin C, Yuefeng C, et al. Stage-oriented Comprehensive Acupuncture Treatment plus Rehabilitation Training for Apoplectic Hemiplegia. *J Tradit Chin Med* 2008;28(2):90-93.
54 Ochi M, Wada F, Saeki S, et al. Gait training in subacute non-ambulatory stroke patients using a full weight-bearing gait-assistance robot: A prospective, randomized, open, blinded-endpoint trial. *J Neurol Sci* 2015;353:130-136.
55 Rosewilliam S, Malhotra S, Roffe C, et al. Can surface neuromuscular electrical stimulation of the wrist and hand combined with routine therapy facilitate recovery of arm function in patients with stroke? *Arch Physl Med Rehab* 2012;93(10):1715-1721.
56 Sanchez-Sanchez ML, Belda-Lois JM, Horno SM, et al. Functional principal component analysis as a new methodology for the analysis of the impact of two rehabilitation protocols in functional recovery after stroke. *J Neuroeng Rehabil* 2014;11:134.
57 Tang Q, Tan L, Li B, et al. Early Sitting, Standing, and Walking in Conjunction With Contemporary Bobath Approach for Stroke Patients With Severe Motor Deficit. *Top Stroke Rehabil* 2014;21(2):120-127.
58 Bai YL, Hu YS, Wu Y, et al. Long-term three-stage rehabilitation intervention alleviates spasticity of the elbows, fingers, and plantar flexors and improves activities of daily living in ischemic stroke patients: a randomized, controlled trial. *Neuroreport* 2014;25:998-1005.
59 Calabro RS, Naro A, Russo M, et al. Do post-stroke patients benefit from robotic verticalization? A pilot-study focusing on a novel neurophysiological approach. *Restor Neurol and Neuros* 2015:33:671-681.

For peer review only - http://bmjopen.bmj.com/site/about/guidelines.xhtml

60 Chaiyawat P and Kulkantrakorn K. Effectiveness of home rehabilitation program for ischemic stroke upon disability and quality of life: A randomized controlled trial. *Clin Neurol Neuros* 2012:114:866-870.
61 Chaiyawat P and Kulkantrakorn K. Randomized controlled trial of home rehabilitation for patients with ischemic stroke: impact upon disability and elderly depression. *Psychogeriatrics* 2012;12:193-199.
62 Jongbloed L, Stacey S and Brighton C. Stroke Rehabilitation: Sensorimotor Integrative Treatment Versus Functional Treatment. *Am J Occup Ther* 1989;43(6):391-397.
63 Kwakkel G, Wagenaar RC, Twisk JWR, et al. Intensity of leg and arm training after primary middle-cerebral artery stroke: a randomised trial. *Lancet* 1999;354:191-196.
64 Kwakkel G, Kollen BJ and Wagenaar RC. Long term effects of intensity of upper and lower limb training after stroke: a randomised trial. *J Neurol Neurosur Ps* 2002;72:473-479.
65 Kwakkel G and Wagenaar RC. Effect of Duration of Upper- and Lower-Extremity Rehabilitation Sessions and Walking Speed on Recovery of Interlimb Coordination in Hemiplegic Gait. *Phys Ther* 2002;82(5):432-448.
66 Morone G, Bragoni M, Iosa M, De Angelis D, Venturiero V, Coiro P. Who May Benefit From Robotic- Assisted Gait Training? A Randomized Clinical Trial in Patients With Subacute Stroke. *Neurorehab Neural Re* 2011;25(7):636-644.
67 Morone G, Iosa M, Bragoni M, De Angelis D, Venturiero V, Coiro P. Who May Have Durable Benefit From Robotic Gait Training? A 2-Year Follow-Up Randomized Controlled Trial in Patients With Subacute Stroke. *Stroke* 2012;43:1140-1142.
68 Yang XH, Bing L and Ba-si O. Effect of acupuncture combined with rehabilitative training on neural functional recovery of stroke patients during recovery phase: a randomized controlled trial. *World J Acupunct Mox* 2014;24(4):17-23.
69 Yue S, Jian X and Wong T. Effects of a nurse-led acupressure programme for stroke patients in China. *J Clin Nurs* 2012;22:1182-1188.
70 Rodrigues TA, Goroso DG, Westgate PM, et al. Slow Versus Fast Robot-Assisted Locomotor Training After Severe Stroke. A Randomized Controlled Trial. *Am J Phys Med Rehab* 2017;96(Suppl):S165–S170.
71 Sackley CM, Walker MF, Burton CR, et al. An occupational therapy intervention for residents with stroke related disabilities in UK care homes (OTCH): cluster randomised controlled trial. *BMJ* 2015;350:h468.
72 Volpe BT, Lynch D, Rykman-Berland A, et al. Intensive Sensorimotor Arm Training Mediated by Therapist or Robot Improves Hemiparesis in Patients With Chronic Stroke. *Neurorehabi Neural Re* 2008;22:305-310.
73 Zhang HY and Li PF. Observation on efficacy of acupuncture combined with rehabilitation training for post-stroke balance disorders. *World J Acupunct Mox* 2014;24(2):25-29.
74 Asplund K and Britton M. Ethics of life support in patients with severe stroke. *Stroke* 1989;20(8):1107-1102.
75 Loubinoux I, Kronenberg G, Endres, M, et al. Post-stroke depression: mechanisms, translation and therapy. *J Cell Mol Med* 2012:16(9):1961-1969.
76 Moore G, Audrey S, Barker M, et al. Process evaluation of complex interventions: Medical Research Council guidance. *BMJ* 2015;350:h1258.

For peer review only

77 McGlinchey M, Walmsley N and Cluckie G. Positioning and pressure care. In: Bhalla A, Birns J,eds. Management of Post-Stroke Complications. Switzerland: Springer 2015:189-225.
78 Dawes M, Summerskill W, Glasziou P, et al. Sicily statement on evidence-based practice. *BMC Med Educ* 2005;5(1) doi:10.1186/1472-6920-5-1.
79 Peters S, Ivanona TD, Teasell R, et al. Is the Recovery of Functional Balance and Mobility Accompanied by Physiological Recovery in People With Severe Impairments After Stroke? *Neurorehab Neural Re* 2014;28(9):847-855.

For peer review only

FIGURE LEGENDS

Figure 1- Flow chart of studies

Figure 2- Risk of bias of individual domains in the included studies

For peer review only - http://bmjopen.bmj.com/site/about/guidelines.xhtml

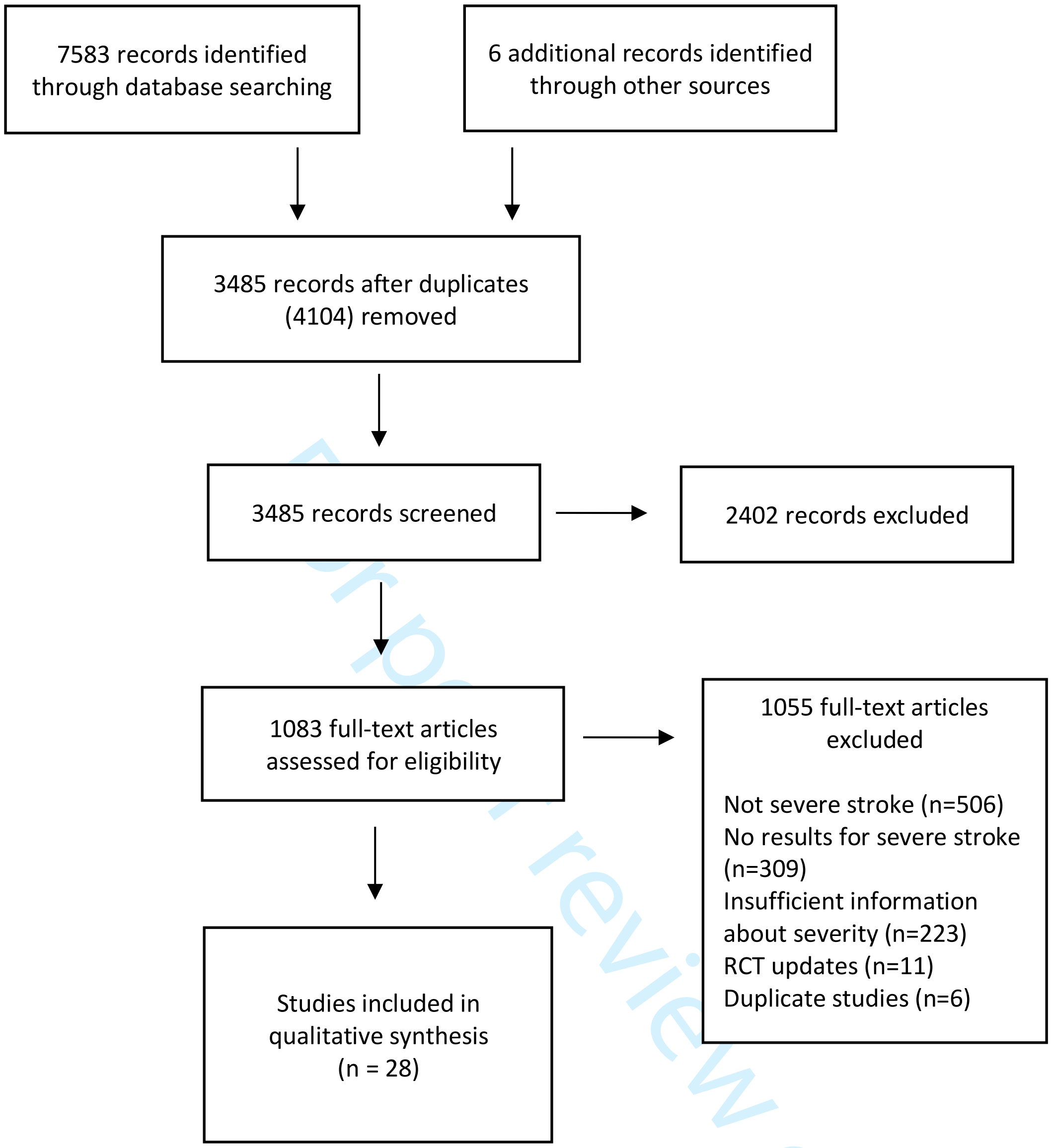

For peer review only

	Random sequence generation (selection bias)	Allocation concealment (selection bias)	Blinding of participants and personnel (performance bias)	Blinding of outcome assessment (detection bias)	Incomplete outcome data (attrition bias)	Selective reporting (reporting bias)	Other bias
AVERT Triallists' Colloboration 2015	+	+	+	+	+	+	?
Bagley et al. 2005	+	+	−	+	+	?	−
Bai et al. 2014	+	?	−	+	+	−	−
Bradley et al. 1998	+	?	−	+	?	?	+
Calabro et al. 2015	+	?	?	+	?	?	+
Chaiyawat and Kulkantrakorn 2012	+	+	−	−	+	?	−
Chang et al. 2012	+	+	?	+	+	?	+
Chen et al. 2011	+	+	?	+	+	?	+
di Lauro et al. 2003	+	+	?	+	+	?	−
Fong et al. 2013	+	+	?	+	+	?	+
Franceschini et al. 2009	+	?	?	+	−	?	?
Jongbloed et al. 1989	+	?	+	+	?	?	+
Katz-Leurer et al. 2003	+	?	?	+	?	?	+
Kwakkel et al. 1999	+	+	+	+	+	?	+
Liang et al. 2012	+	+	?	+	+	?	+
Lincoln et al. 1999	+	+	−	+	?	?	+
Min et al. 2008	?	?	−	?	?	?	+
Morone et al. 2011	+	?	?	+	+	?	+
Ochi et al. 2015	+	+	?	+	+	?	+
Rodrigues et al. 2017	+	+	?	+	?	?	+
Rosewilliam et al. 2012	+	+	?	+	+	?	?
Sackley et al. 2015	+	+	+	+	+	+	+
Sanchez-Sanchez et al. 2014	+	?	−	+	?	?	−
Tang et al. 2014	+	+	+	+	+	?	+
Volpe et al. 2008	?	?	?	+	+	?	+
Yang et al. 2014	+	?	−	?	+	?	+
Yue et al. 2012	+	+	−	?	+	?	+
Zhang and L. 2014	−	?	−	?	?	?	+

For peer review only - http://bmjopen.bmj.com/site/about/guidelines.xhtml

PRISMA 2009 Checklist

Section/topic	#	Checklist item	Reported on page #
**TITLE**			
Title	1	Identify the report as a systematic review, meta-analysis, or both.	Title page
**ABSTRACT**			
Structured summary	2	Provide a structured summary including, as applicable: background; objectives; data sources; study eligibility criteria, participants, and interventions; study appraisal and synthesis methods; results; limitations; conclusions and implications of key findings; systematic review registration number.	Abstract page
**INTRODUCTION**			
Rationale	3	Describe the rationale for the review in the context of what is already known.	1-2
Objectives	4	Provide an explicit statement of questions being addressed with reference to participants, interventions, comparisons, outcomes, and study design (PICOS).	2
**METHODS**			
Protocol and registration	5	Indicate if a review protocol exists, if and where it can be accessed (e.g., Web address), and, if available, provide registration information including registration number.	Abstract page, 2
Eligibility criteria	6	Specify study characteristics (e.g., PICOS, length of follow-up) and report characteristics (e.g., years considered, language, publication status) used as criteria for eligibility, giving rationale.	3
Information sources	7	Describe all information sources (e.g., databases with dates of coverage, contact with study authors to identify additional studies) in the search and date last searched.	4
Search	8	Present full electronic search strategy for at least one database, including any limits used, such that it could be repeated.	Supplementary file
Study selection	9	State the process for selecting studies (i.e., screening, eligibility, included in systematic review, and, if applicable, included in the meta-analysis).	3, 4
Data collection process	10	Describe method of data extraction from reports (e.g., piloted forms, independently, in duplicate) and any processes for obtaining and confirming data from investigators.	3, 4
Data items	11	List and define all variables for which data were sought (e.g., PICOS, funding sources) and any assumptions and simplifications made.	3
Risk of bias in individual studies	12	Describe methods used for assessing risk of bias of individual studies (including specification of whether this was done at the study or outcome level), and how this information is to be used in any data synthesis.	4
Summary measures	13	State the principal summary measures (e.g., risk ratio, difference in means).	N/A
Synthesis of results	14	Describe the methods of handling data and combining results of studies, if done, including measures of consistency (e.g., I^2) for each meta-analysis.	N/A

For peer review only - http://bmjopen.bmj.com/site/about/guidelines.xhtml

PRISMA 2009 Checklist

Section/topic	#	Checklist item	Reported on page #
Risk of bias across studies	15	Specify any assessment of risk of bias that may affect the cumulative evidence (e.g., publication bias, selective reporting within studies).	N/A
Additional analyses	16	Describe methods of additional analyses (e.g., sensitivity or subgroup analyses, meta-regression), if done, indicating which were pre-specified.	N/A
**RESULTS**			
Study selection	17	Give numbers of studies screened, assessed for eligibility, and included in the review, with reasons for exclusions at each stage, ideally with a flow diagram.	5
Study characteristics	18	For each study, present characteristics for which data were extracted (e.g., study size, PICOS, follow-up period) and provide the citations.	Supplementary file
Risk of bias within studies	19	Present data on risk of bias of each study and, if available, any outcome level assessment (see item 12).	5
Results of individual studies	20	For all outcomes considered (benefits or harms), present, for each study: (a) simple summary data for each intervention group (b) effect estimates and confidence intervals, ideally with a forest plot.	Supplementary file, 6-9
Synthesis of results	21	Present results of each meta-analysis done, including confidence intervals and measures of consistency.	N/A
Risk of bias across studies	22	Present results of any assessment of risk of bias across studies (see Item 15).	N/A
Additional analysis	23	Give results of additional analyses, if done (e.g., sensitivity or subgroup analyses, meta-regression [see Item 16]).	N/A
**DISCUSSION**			
Summary of evidence	24	Summarize the main findings including the strength of evidence for each main outcome; consider their relevance to key groups (e.g., healthcare providers, users, and policy makers).	9-12
Limitations	25	Discuss limitations at study and outcome level (e.g., risk of bias), and at review-level (e.g., incomplete retrieval of identified research, reporting bias).	12
Conclusions	26	Provide a general interpretation of the results in the context of other evidence, and implications for future research.	12, 13
**FUNDING**			
Funding	27	Describe sources of funding for the systematic review and other support (e.g., supply of data); role of funders for the systematic review.	13

From: Moher D, Liberati A, Tetzlaff J, Altman DG, The PRISMA Group (2009). Preferred Reporting Items for Systematic Reviews and Meta-Analyses: The PRISMA Statement. PLoS Med 6(7): e1000097. doi:10.1371/journal.pmed1000097

For more information, visit: **www.prisma-statement.org**.

For peer review only

For peer review only - http://bmjopen.bmj.com/site/about/guidelines.xhtml

Supplementary File 2

Supplementary Material- Medline Search Strategy

1. exp Stroke/
2. severe stroke.mp.
3. stroke severit*.mp.
4. stroke disabilit*.mp.
5. exp Physical Therapy Modalities/
6. exp Occupational Therapy/
7. exp Nursing Care/
8. physical rehabilitation.mp.
9. exp Stroke Rehabilitation/
10. exp Patient Positioning/
11. exp Posture/
12. exp Exercise/
13. exp Exercise Therapy/
14. passive exercise.mp.
15. exp "Range of Motion, Articular"/
16. manual technique.mp.
17. active exercise.mp.
18. Resistance Training/
19. exp Muscle Stretching Exercises/
20. exp Electric Stimulation/
21. exp Electric Stimulation Therapy/
22. exp Wheelchairs/
23. seat?.mp.
24. exp "Equipment and Supplies"/
25. exp Teaching/
26. exp Education/
27. exp Motor Skills/
28. exp Movement/
29. motor function.mp.
30. motor recovery.mp.
31. exp "Recovery of Function"/
32. exp "Activities of Daily Living"/
33. functional independence.mp.
34. physical independence.mp.
35. complicatio*.mp.
36. exp Pain/
37. exp Contracture/
38. exp Pressure Ulcer/
39. exp Respiratory Tract Infections/
40. exp Urinary Tract Infections
41. Muscle Spasticity/
42. Venous Thrombosis/
44. exp Pulmonary Embolism/
44. exp Accidental Falls/
45. exp Fatigue/
46. exp Depression/
47. 1 or 2 or 3 or 4

For peer review only

48. 5 or 6 or 7 or 8 or 9 or 10 or 11 or 12 or 13 or 14 or 15 or 16 or 17 or 18 or 19 or 20 or 21 or 22 or 23 or 24 or 25 or 26
49. 27 or 28 or 29 or 30
50. 31 or 32 or 33 or 34
51. 35 or 36 or 37 or 38 or 39 or 40 or 41 or 42 or 43 or 44 or 45 or 46
52. 47 and 48 and 49
53. 47 and 48 and 50
54. 47 and 48 and 51
55. limit 52 to ("all adult (19 plus years)" and randomized controlled trial)
56. limit 53 to ("all adult (19 plus years)" and randomized controlled trial)
57. limit 54 to ("all adult (19 plus years)" and randomized controlled trial)

For peer review only

Supplementary Table 1- Studies conducted in the acute – early subacute (<3 months) phase post-stroke

Study	Intervention Description	Intervention Duration	Intervention Delivered By	Stroke Severity Measure	Sample Size and Characteristics	Main Outcome Measures	Main Results	Quality of Evidence
AVERT trial collaboration group 2015 [1]	Very early mobilisation vs Usual care	Up to 14 days	PT and nursing staff	NIHSS	Very early mobilisation group NIHSS >16 (n=147)<br>Usual care group NIHSS >16 (n=144)	Favourable outcome (mRS 0-2) and mortality at 3 months	No difference in favourable outcome or mortality between groups	High
Bagley et al. 2005 [2]	Oswestry standing frame + standard physiotherapy vs Standard physiotherapy	14 daily sessions	PTs	BI^	Oswestry group (n=71) Median BI 1 (IQR 0-3)<br>Control group (n=69) Median BI 2 (IQR 1-3)	RMI, BI, HADS, NEADL, RMA, MAS (balance, sit to stand sections), TCT, CSI, GHQ-28	No differences between groups for all outcome measures. No differences in number of treatment sessions between groups or number of staff members required to treat each patient.	Low
Bradley et al. 1998 [3]	EMG biofeedback + conventional physiotherapy vs Placebo EMG + conventional physiotherapy	6 weeks	PTs	RMI	EMG group RMI ≤3 (n=7)<br>Conventional PT group RMI ≤3 (n=6)	MBS, mAS, 10MWT, RMI, sensation, proprioception NEADL	No differences between groups for MBS, RMI, NEADL and 10MWT. No improvements in mAS, sensation and proprioception for both groups.	Very low
Chang et al. 2012 [4]	Robot-assisted BWS treadmill gait training + conventional physiotherapy vs Conventional physiotherapy	2 weeks	PTs	FAC<br>LL FMA	Robot-assisted group (n=20) Mean FAC 0.5 (SD 0.5) Mean LL FMA 17.2 (SD 5.5)<br>Conventional group (n=17) Mean FAC 0.4 (SD 0.5) Mean LL FMA 16.8 (SD 5.7)	FAC, LL MI, LL FMA, Peak VO_2	Improvements in LL FMA and peak VO_2 in robot-assisted gait training group. No improvements in LL MI and FAC for both groups.	Low

For peer review only

Chen et al. 2011 [5]	Thermal stimulation + standard rehabilitation vs Standard rehabilitation	6 weeks	Thermal stimulation- PTs<br><br>Standard rehabilitation- PTs and OTs	FAC<br>LL FMA	Thermal stimulation group (n=17)<br>Median FAC 0 (IQR 0-1)<br>Median LL FMA 7 (4-11.5)<br><br>Standard rehab group (n=16)<br>Median FAC 0 (IQR 0-1)<br>Median LL FMA 6 (4.3-12.0)	LL FMA, LL MRC, mAS, mMAS, PASS (trunk control items), BBS, FAC	Thermal stimulation group demonstrated greater recovery gains compared to standard rehabilitation group in all outcomes except PASS. No difference between groups in MAS.	Low
Di Lauro et al. 2003 [6]	Intensive rehabilitative treatment vs Ordinary rehabilitative treatment	14 days	Therapists and nursing staff	BI^	Intensive rehab group (n=29)<br>Mean BI 1.4 (SD 1.4)<br><br>Ordinary rehab group (n=31)<br>Mean BI 1.5 (SD 1.5)	BI, mNIHSS	No differences between groups in BI or mNIHSS	Very low
Fong et al. 2013 [7]	Cueing wristwatch + conventional rehabilitation vs Sham wristwatch + conventional rehabilitation	3 weeks	Wristwatch- OTs<br><br>Conventional rehab- OT, PT, ST	Motor FIM	Cueing wristwatch group (n=19) Mean motor FIM 25.6 (SD 8.3)<br><br>Sham wristwatch group (n=16)<br>Mean motor FIM 28.2 (SD 10.0)	UL FMA, FTHUE, motor FIM, total number of UL movements	No differences between groups for UL FMA, FTHUE and motor FIM. More total UL movements in cueing wristwatch group but not significantly different between groups.	Low
Franceschini et al. 2009 [8]	BWS treadmill gait training + conventional treatment vs Conventional treatment	4 weeks	PTs	BI^	Treadmill training group (n=52)<br>Median BI 6 (IQR 3-9)<br>Median FAC 0 (IQR 0-0)<br><br>Conventional group (n=45)<br>Median BI 5 (IQR 3-7)<br>Median FAC 0 (IQR 0-0)	MI, TCT, mRS, BI, FAC, AS, LL proprioception, 6MWT, 10MWT, BS, WHS	No differences between groups. All patients were able to walk at discharge.	Low

For peer review only

Katz-Leurer et al. 2003 [9]	Leg cycle ergometer + regular therapy vs Regular therapy	8 weeks	Leg cycle ergometer- PTs<br><br>Regular therapy- PT, OT, ST	SSS	Leg cycle ergometer and regular rehabilitation groups- actual number of patients with severe stroke (SSS <30) not reported	FAI	No differences in decline in FAI between groups	Low
Liang et al. 2012 [20]	Thermal stimulation + standard rehabilitation vs Standard rehabilitation	6 weeks	Thermal stimulation- PTs<br><br>Standard rehabilitation- PTs and OTs	BI*	Thermal stimulation group (n=15)<br>Mean BI 30.3 (SD 11.1)<br><br>Standard rehab group (n=15)<br>Mean BI 27.7 (SD 14.3)	LL FMA, LL MRC, FAC, BBS, mMAS, BI	Improvements in LL FMA, LL MRC, FAC and mMAS in thermal stimulation group post-intervention and at 3-month follow-up. Improvements in BBS and BI in thermal stimulation group only at 3-month follow-up. Except for LL-FMA, all improvements disappeared at 6-month and 12-month follow-up.	Low
Lincoln et al. 1999 [11]	Standard physiotherapy + additional qualified PT therapy vs Standard physiotherapy + additional PTA therapy vs Standard physiotherapy	5 weeks	PTs/ PTAs	BI^	Qualified PT group (n=94)<br>Median BI 6 (IQR 3-9)<br><br>PTA group (n=93)<br>Median BI 6 (IQR 4-8)<br><br>Standard PT group (n=95)<br>Median BI 7 (IQR 3-9)	RMA- arm scale, ARAT, THPT, grip strength, mAS, BI, MCA	No differences between the groups across all outcomes	Low
Min et al. 2008 [12]	Acupuncture + systemic functional exercise vs Systemic functional exercise	? 3 months	Not reported	BI*	Acupuncture group (n=30)<br>Mean BI 27.28 (SD 5.41)<br><br>Systemic exercise group (n=30)<br>Mean BI 28.01 (SD 4.48)	FMA, BI	Acupuncture group demonstrated greater improvements in FMA and BI compared to the systemic exercise group	Very low

For peer review only

Ochi et al. 2015 [13]	Robot-assisted treadmill gait training + standard physiotherapy vs Conventional overground gait training + standard physiotherapy	4 weeks	Robot-assisted gait training- not reported Conventional gait training- PTs	FIM mobility FAC	Robot-assisted group (n=13) Median FAC 0 (IQR 0-1) Median FIM mobility 7 (IQR 6-10) Conventional group (n=13) Median FAC 1 (IQR 0-1) Median FIM mobility 7 (IQR 7-9)	FAC, FMA, LL muscle torque, 10MWT, FIM (mobility scores)	Robot-assisted gait training group demonstrated greater improvements in FAC and peak LL muscle torque compared to the conventional group	Low
Rosewilliam et al. 2012 [14]	Wrist and finger NMES + usual care vs Usual care	6 weeks	NMES- staff group not reported, patients and carers Usual care- PTs	BI^	NMES group (n=31) Mean BI 4.4 (SD 3.9) Mean ARAT 0.0 (SD 0.0) Usual care group (n=36) Mean BI 2.5 (SD 2.9) Mean ARAT 0.6 (SD 3.5)	ARAT, BI, wrist AROM, wrist strength, grip strength	No differences in ARAT, BI or wrist AROM between groups. Improvements in wrist extensor and grip strength in the NMES group post-intervention but not maintained at follow-up.	Moderate
Sanchez-Sanchez et al. 2014 [15]	Functionally targeted physiotherapy techniques + conventional physiotherapy vs Conventional physiotherapy	Not reported	PTs	BI*	Functional techniques group (n=5) Mean BI 13 (SD 10.95) Conventional therapy group (n=8) Mean BI 11.43 (SD 13.13)	BI	Functionally targeted physiotherapy group demonstrated greater improvement compared to the conventional physiotherapy group when using functional principal component analysis	Very low
Tang et al. 2014 [16]	Contemporary Bobath approach with early sitting, standing and walking vs Contemporary Bobath approach	8 weeks	PTs	STREAM BBS	Early contemporary group (n=24) Mean STREAM 1.4 (SD 1.0) Mean BBS 0 (SD 0) Contemporary group (n=24) Mean STREAM 1.3 (SD 0.9) Mean BBS 0 (SD 0)	STREAM, BBS	Improvements in STREAM and BBS in the contemporary Bobath approach with early mobilisation group	Low

For peer review only

Supplementary Table 2- Studies conducted in the acute – late subacute (<6 months) phase post-stroke

Study	Intervention Description	Intervention Duration	Intervention Delivered By	Stroke Severity Measure	Sample Size and Characteristics	Main Outcome Measures	Main Results	Quality of Evidence
Bai et al. 2014 [17]	Staged physical rehabilitation interventions + routine care vs Routine care	6 months	PTs and OTs	BI*	Staged rehab group (n=83) Mean BI 28 (range 24-31)<br><br>Routine care group (n=82) Mean BI 23 (range 19-27)	BI, mAS	Staged rehab group demonstrated higher BI scores than the routine care group at 1, 3- and 6-months post-stroke. 42.9% of patients in the routine care group demonstrated spasticity in at least one body part compared to 36.4% of patients in the staged rehab group.	Low
Calabrò et al. 2015 [18]	Robotic verticalisation + standard physiotherapy vs Physiotherapy-assisted verticalisation + standard physiotherapy	6 weeks	PTs	PASS LL FMA	Robotic group (n=10) Mean PASS 3 (SD 1) Mean LL FMA 13 (SD 3)<br><br>Physiotherapy group (n=10) Mean PASS 3 (SD 3) Mean LL FMA 12 (SD 6)	PASS, LL FMA, MRC, vertical posture tolerance	Both interventions were well tolerated. Robotic group demonstrated greater improvements in MRC, LL FMA and PASS compared to the physiotherapy group	Very low
Chaiyawat and Kulkantrakorn 2012 [19,20]	Home based physiotherapy programme vs Usual care	6 months	PTs	BI*	Home PT group (n=30) Mean BI 31.7 (SD 5.9) Mean NIHSS 16.4 (SD 4.1)<br><br>Usual care group (n=30) Mean BI 33.2 (SD 4.8) Mean NIHSS 17.8 (SD 3.9)	BI, HADS, mRS, EQ-5D	Home therapy group demonstrated greater improvements in BI, HADS, mRS and EQ-5D compared to the usual care group which were maintained at 2-year follow-up.	Very low
Jongbloed et al. 1989 [21]	Functional treatment approach vs Sensorimotor integrative treatment approach	8 weeks	OTs	BI*	Functional treatment group (n=13) Mean BI 31.5<br><br>Sensorimotor integrative treatment group (n=9) Mean BI 30	BI, meal preparation, eight subtests of Sensorimotor Integration Test Battery	No differences between groups on all outcome measures	Very low

For peer review only

Kwakkel et al.<br>1999 [22]<br>2002 [23]<br>2002 [24]	Additional UL training + usual care<br>vs<br>Additional LL training + usual care<br>vs<br>UL/LL pressure splint immobilisation + usual care	20 weeks	PTs and OTs	BI^	UL training group (n=33)<br>Median BI 5 (IQR 3-7)<br>LL training (n=31)<br>Median BI 6 (IQR 3-8)<br>Splint control group (n=37)<br>Median BI 5.5 (IQR 3-7)	BI, FAC, ARAT, 10MWT, SIP, NHP, FAI	LL training group had significantly higher BI, FAC, walking speed and ARAT than splint control group post-intervention. UL training group had significantly higher ARAT than splint control group post-intervention. No significant differences in all outcomes were seen between groups from 6 months onwards up until 12-month follow-up.	Moderate
					CRP sub-study<br>UL training group (n=18)<br>Mean BI 5.0 (SD 2.0)<br>LL training (n=17)<br>Mean BI 6.3 (SD 2.7)<br>Splint control group (n=18)<br>Mean BI 5.3 (SD 2.7)	10MWT, mean CRP of arm/leg movements	LL training group had significantly higher comfortable walking speed than UL and splint control groups post-intervention. No differences were seen for the mean CRP of arm/leg movements between groups.	
Morone et al.<br>2011 [25]<br>2012 [26]	Robot-assisted BWS treadmill gait training + standard physiotherapy<br>vs<br>Conventional gait training + standard physiotherapy	3 months	PTs	BI*	Robotic groups<br>Low motricity (n=12)<br>Mean BI 14.2 (SD 11.8)<br>High motricity (n=12)<br>Mean BI 20.0 (SD 17.2)<br>Conventional groups<br>Low motricity (n=12)<br>Mean BI 7.9 (SD 8.9)<br>High motricity (n=12)<br>Mean BI 24.6 (SD 15.3)	FAC, LL AS, RMI, MI, TCT, CNS, BI, RS, 6MWT, 10MWT	Higher FAC in low motricity robotic training group compared to low motricity conventional training group post-intervention. At discharge, higher RMI, BI, TCT, RS and 6MWT in low motricity robotic training group compared to low motricity conventional training group. No differences were seen between the higher motricity groups post-intervention or on discharge.<br>At 12-month follow-up, low motricity robotic training group had higher FAC, BI and RMI compared to low motricity conventional training group. No differences were seen between the higher motricity groups.	Very low

For peer review only

Yang et al. 2014 [27]	Acupuncture + rehabilitation training<br>vs<br>Rehabilitation training	8 weeks	Acupuncture-not reported<br><br>Rehabilitation-PTs	NIHSS<br>BI*	Acupuncture group (n=33)<br>Mean NIHSS 25.5 (SD 2.4)<br>Mean BI 39.4 (SD 3.9)<br><br>Rehabilitation group (n=31)<br>Mean NIHSS 24.1 (SD 3.1)<br>Mean BI 38.1 (SD 4.3)	NIHSS, FMA, BI	Acupuncture group demonstrated higher scores on all outcome measures compared to the rehabilitation group	Very low
Yue et al. 2012 [28]	Acupressure treatment + routine care<br>vs<br>Routine care	3 months	Nurses	BI*	Acupressure group (n=35)<br>Mean BI 26.8 (SD 15.2)<br><br>Routine care group (n=34)<br>Mean BI 24.4 (SD 16.8)	FMA, BI	Acupressure group demonstrated greater improvements in BI and FMA only at 3-month time frame	Very low

For peer review only

Supplementary Table 3- Studies conducted in the chronic (>6 months) phase post-stroke

Study	Intervention Description	Intervention Duration	Intervention Delivered By	Stroke Severity Measure	Sample Size and Characteristics	Main Outcome Measures	Main Results	Quality of Evidence
Rodrigues et al. 2017 [29]	Robot-assisted BWS treadmill gait training with progressively increased speeds vs Robot-assisted bodyweight supported treadmill gait training with progressively decreased speeds	6 weeks	Not reported	LL FMA FAC	Faster speed group (n=10) Median FAC 1.5 (1–2) Mean LL FMA 19.5 (SD 4.6) Slower speed group (n=10) Median FAC 1 (1–2) Mean LL FMA 17.5 (SD 2.8)	FAC, TUG, 6MWT, 10MWT, BBS, LL FMA	Improvements in FAC, FMA, TUG and 6MWT in the slower speed group compared to the faster speed group.	Very low
Sackley et al. 2015 [30]	OT intervention vs Usual care	3 months	OTs	BI^	OT intervention group- BI 0-4 n=268 BI 5-9 n=129 Usual care group- BI 0-4 n=234 BI 5-9 n=104	BI, RMI, GDS, EQ-5D-3L	No differences between the groups on any outcome measure at 3-, 6- and 12-months post-randomisation. Higher fall rate per resident in OT intervention group at 3 months.	High
Volpe et al. 2008 [31]	Intensive standard UL therapy vs Intensive robot-assisted UL therapy	6 weeks	Therapists	NIHSS	Therapist group (n=10) Mean NIHSS 17 (SD 1) Robot group (n=11) Mean NIHSS 17 (SD 1)	FMA- UL, MRC-shoulder/ elbow, mAS, UL PROM, SIS, ARAT, BDS, shoulder dislocation, pain	No difference between groups in shoulder and elbow strength and motor function. No improvements in other outcome measures for both groups.	Very low
Zhang and Li 2014 [32]	Trunk acupuncture + rehabilitation training vs Rehabilitation training alone	16 weeks	Not reported	BI*	Acupuncture group (n=30) Mean BI 22.50 (SD 6.79) Rehabilitation group (n=29) Mean BI 24.48 (SD 7.23)	BI, BBS	Acupuncture group demonstrated higher scores on BI and BBS compared to the rehabilitation group.	Very low

For peer review only

ARAT- Action Research Arm Test, AROM- active range of movement, AS- Ashworth Scale, BBS- Berg Balance Scale, BDS- Becks Depression Scale, BI*- Barthel Index (original version scored out of 100), BI^ - Barthel Index(revised version score out of 20), BS- Borg Scale, BWS- bodyweight supported, CNS- Canadian Neurological Scale, CRP- continuous relative phase, CSI- Caregiver Strain Index, EQ-5D-3L- EuroQoL questionnaire, FAC- Functional Ambulation Category, FAI- Frenchay Activities Index, FIM- Functional Independence Measure, FMA- Fugl-Meyer Assessment, FTHUE- Functional Test for the Hemiplegic Upper Extremity, GDS- Geriatric Depression Scale, GHQ-28- General Health Questionnaire-28, HADS- Hospital Anxiety and Depression Scale, LL- lower limb, MAS- Motor Assessment Scale, mAS- Modified Ashworth Scale, MCA- Motor Club Assessment, MI- Motricity Index, mMAS- Modified Motor Assessment Scale, MMSE- Mini-Mental State Examination, mNIHSS- Modified National Institutes of Health Stroke Scale, mRS- Modified Rankin Scale, MRC- Medical Research Council Scale for Muscle Strength, NEADL- Nottingham Extended Activities of Daily Living, NHP- Nottingham Health Profile, NIHSS- National Institutes of Health Stroke Scale, OT- occupational therapist, PASS- Postural Assessment Scale for Stroke Patients, PROM- passive range of movement, PT- physiotherapist, PTA- physiotherapy assistant, RMA- Rivermead Motor Assessment, RMI- Rivermead Mobility Index, RS- Rankin Scale, SIP- Stroke Impact Profile, SIS- Stroke Impact Scale, ST- speech therapist, STREAM- Stroke Rehabilitation Assessment of Movement, TCT- Trunk Control Test, THPT- Ten-Hole Peg Test, TUG- Timed Up and Go, UL- upper limb, WHS- Walking Handicap Scale, 6MWT- 6 minute walk test, 10MWT- 10 metre walk test

For peer review only

Supplementary Table 4- Overview of Measures of Physical Function and Immobility-Related Complications

Body Function	Activity	Participation	Complications
**Cardiorespiratory Function**	**Activities of Daily Living**	**Instrumental Activities of Daily Living**	**Adverse Effects**
Aerobic capacity	Barthel Index	Frenchay Activities Index	Pain
Borg scale	Functional Independence Measure- motor	Nottingham Extended ADL Scale	Shoulder dislocation
Cardiovascular response	Functional Independence Measure- total	Meal preparation	
Ventilatory response	Modified Rankin Scale		
			**Caregiver Burden**
		**Perceived Health Status**	Caregiver Strain Index
**Neurological Impairment**	**Balance and Postural Control**	Stroke Impact Scale	
Canadian Neurological Scale	Berg Balance Scale	General Health Questionnaire-28	
National Institutes of Health Stroke Scale	Postural Assessment Scale for Stroke		**Depression**
	Trunk Control Test		Beck Depression Scale
	Vertical Posture Test	**Quality of Life**	Geriatric Depression Scale
**Sensorimotor Function**		EQ-5D	Hospital and Depression Scale
Active range of movement- UL		Nottingham Health Profile	
Grip strength	**Gait**	Sickness Impact Profile	
Fugl Meyer- UL	Continuous relative phase between UL/LL movement		**Mortality**
Fugl Meyer- LL	Comfortable/maximal walking speed		Mortality
Fugl Meyer- UL and LL	Functional Ambulation Category		
Motricity Index	Number of independent walkers		
Medical Research Council strength- UL	Time taken to walk 50 metres independently		**Spasticity**
Medical Research Council strength- LL	Walking Handicap Scale		Modified Ashworth Scale
Medical Research Council strength- UL and LL	6 minutes walking test		
Number of upper limb movements	10 metre walking test		
Sensation/proprioception			
Sensorimotor integration test			
	**General Physical Activity**		
	Modified Bobath Scale		
	Motor Assessment Scale		
	Rivermead Motor Assessment		
	Rivermead Mobility Index		
	Stroke Rehabilitation Assessment of Movement		
	Timed Up and Go		
	**Upper Limb Function**		
	Action Research Arm Test		
	Functional Test for Hemiplegic Upper Extremity		
	9 Hole Peg Test		
	10 Hole Peg Test		

For peer review only

Supplementary Results- Outcomes Supported by Low or Very Low-Quality Evidence

Body function

Cardiorespiratory Function

Two studies explored participants' cardiorespiratory response to different types of treadmill gait training within the acute to early subacute phase post-stroke.[4,8] There was low-quality evidence that 2 weeks of robot-assisted bodyweight supported treadmill gait training delivered in the first 6 weeks post-stroke improved peak VO_2 compared to conventional gait training.[4] There was low-quality evidence that a 4-week course of bodyweight supported treadmill training delivered in the first 3 months post-stroke was not perceived to be more effortful than conventional gait training.[8]

Neurological Impairment

Three studies evaluated changes in neurological function.[6,25,27] In the acute to early subacute phase post-stroke, there was very low-quality evidence that there was no difference in an intensive or ordinary 2-week acute physical rehabilitation programme on reducing neurological impairment at 2 weeks and 6 months post-stroke.[6] In the acute to late subacute phase post-stroke, there was very low-quality evidence that a 3-month course of robot-assisted bodyweight supported treadmill gait training commenced within the first 6 weeks post-stroke was just as effective as conventional gait training on improving neurological function.[25] There was very low-quality evidence that an 8-week course of acupuncture provided in conjunction with rehabilitation during the subacute phase of stroke reduced neurological impairment compared to rehabilitation alone.[27]

Sensorimotor Function

Sixteen studies evaluated changes in sensorimotor function. Nine studies were performed in the acute to early subacute phase post-stroke,[3-5,7,8,10-13] five studies in the acute to late subacute phase post-stroke,[18,21,25,27,28] and two studies in the chronic phase post-stroke.[29,31] In the acute to early subacute phase post-stroke, there was low quality evidence from two studies that thermal stimulation in conjunction with standard rehabilitation resulted in improvements in lower limb sensorimotor function and strength when compared to standard rehabilitation alone.[5,10] Improvements in lower limb sensorimotor function were maintained at 12 months post-intervention. There was low quality evidence that 2 weeks of robot-assisted bodyweight supported treadmill gait training resulted in improvements in lower limb sensorimotor function but not strength compared to conventional gait training.[4] There was low quality evidence that there was no difference between: 4 weeks of robot-assisted treadmill gait training and conventional gait training on improving lower limb sensorimotor function;[13] wearing a cueing wristwatch and wearing a sham wristwatch for 3 hours per weekday for 3 weeks during rehabilitation on improving upper limb sensorimotor function and number of arm movements;[7] a 4-week course of bodyweight supported treadmill training and conventional overground gait training on improving lower limb strength;[8] and a 5-week course of additional upper limb therapy provided by a qualified physiotherapist or a physiotherapy assistant and standard physiotherapy on improving upper limb motor activity and grip strength.[11]

There was very low-quality evidence that a thrice weekly, 6-week course of electromyography (EMG) biofeedback combined with conventional physiotherapy had no effect on improving lower limb active range of movement when compared to conventional

For peer review only

physiotherapy alone.[3] There was very low-quality evidence that a 3-month course of acupuncture in conjunction with rehabilitation resulted in better upper and lower limb sensorimotor function when compared to rehabilitation alone.[12]

In the acute to late subacute phase post-stroke, there was very low quality evidence that a 6-week course of robotic tilt-table verticalisation that combines cyclic leg movements and FES and used in conjunction with standard physiotherapy resulted in better lower limb strength and sensorimotor function compared to physiotherapy-assisted verticalisation using a standard tilt-table and used in conjunction with standard physiotherapy.[18] There was very low-quality evidence that an 8-week course of acupuncture provided in conjunction with rehabilitation resulted in improvements in upper and lower limb sensorimotor function compared to rehabilitation alone.[27] There was very low-quality evidence that a 3-month course of nurse-led acupressure resulted in improvements in upper and lower limb motor function compared to routine care.[28] There was very low quality evidence that there was no difference between: a functionally-orientated and a sensorimotor integrative occupational therapy treatment approach delivered over 8 weeks on improving upper limb sensorimotor function;[21] and a 3-month course of robot-assisted bodyweight supported treadmill gait training and conventional gait training on improving lower limb power.[25]

In the chronic phase post-stroke, there was very low-quality evidence that a 6-week course of robot-assisted bodyweight supported treadmill gait training using slower treadmill speeds resulted in improvements in lower limb sensorimotor function compared to similar treadmill training using faster treadmill speeds.[29] There was very low-quality evidence that either an intensive therapist-driven UL protocol or an intensive robotic-driven UL protocol delivered thrice weekly for 6 weeks resulted in an improvement in shoulder and elbow sensorimotor function.[31]

Activity

Activities of Daily Living

Sixteen studies explored independence and ability to perform activities of daily living (ADLs). Nine studies were completed in the acute to early subacute phase,[2,6-8,10-13,15] six studies were completed in acute to late subacute phase[17,19-21,25,27,28] and one study was completed in the chronic phase.[32]

In the acute to early subacute phase, there was low quality evidence that a 6-week course of thermal stimulation used in conjunction with standard rehabilitation resulted in improvements in ADL independence 3 months post-stroke compared to standard rehabilitation alone, although improvements were not seen at 6 months post-stroke.[10]

There was low quality evidence that there was no difference between: regular physiotherapy and regular physiotherapy in conjunction with use of an Oswestry standing frame delivered over 14 consecutive weekdays in the first 3 months post-stroke on ADL independence;[2] wearing a cueing wristwatch and wearing a sham wristwatch for 3 hours per weekday for 3 weeks during rehabilitation on ADL independence;[7] a 4-week course of bodyweight supported treadmill training and conventional overground gait training on improving ADL independence;[8] a 5-week course of additional upper limb therapy provided by a qualified physiotherapist or a physiotherapy assistant and standard physiotherapy on improving ADL independence;[11] and 4 weeks of robot-assisted treadmill gait and conventional overground gait training on ADL independence.[13]

There was very low-quality evidence that there was no difference in an intensive or ordinary 2-week acute physical rehabilitation programme in improving ADL independence at 2 weeks

For peer review only

and 6 months post-stroke.[6] There was very low-quality evidence that a 3-month course of acupuncture in conjunction with rehabilitation resulted in better ADL independence when compared to rehabilitation alone.[12] There was very low-quality evidence that providing additional physiotherapy in conjunction to regular rehabilitation in the first few weeks post-stroke resulted in improvements in ADL independence at 6 months post-stroke compared to regular rehabilitation alone.[15]

In the acute to late subacute phase, there was low quality evidence that a 6-month course of a staged physical rehabilitation programme resulted in greater improvements in ADL independence compared to usual care that did not involve formal rehabilitation.[17] There was very low-quality evidence that a monthly home-based physiotherapy programme delivered over 6 months resulted in improvements in ADL independence compared to standard care.[19,20] There was very low-quality evidence that there was no difference between a functionally orientated or a sensorimotor integrative occupational therapy treatment approach delivered over 8 weeks on ADL independence.[21] There was very low-quality evidence that a 3-month course of robot-assisted bodyweight supported treadmill gait training resulted in improvements in ADL independence compared to conventional gait training.[25] Improvements were only seen in the cohort of participants who demonstrated significant motor impairment. Improvements were maintained at the 2-year follow-up.[26]

There was very low-quality evidence that an 8-week course of acupuncture provided in conjunction with rehabilitation during the subacute phase of stroke improved ADL independence compared to rehabilitation alone.[27] There was very low-quality evidence that a 3-month course of nurse-led acupressure resulted in improvements in ADL independence compared to routine care.[28]

In the chronic phase, there was very low-quality evidence that a 16-week course of trunk acupuncture combined with rehabilitation training resulted in greater improvements in ADL independence compared to rehabilitation training alone.[32]

Balance and Postural Control

Eight studies investigated balance and postural control. Four studies were completed in the acute to early subacute phase,[2,5,10,16] two studies were completed in the acute to late subacute phase[18,25] and two studies were completed in the chronic phase.[29,32]

In the acute to early subacute phase, there was low quality evidence that a 6-week course of thermal stimulation in conjunction with standard rehabilitation resulted in improvements in trunk postural control but not balance compared to standard rehabilitation alone.[5] In a separate study, there was low quality evidence that a 6-week course of thermal stimulation in conjunction with standard rehabilitation resulted in improvements in balance 3 months post-stroke compared to standard rehabilitation alone, although improvements were not seen at 6 months post-stroke.[10] There was low quality evidence that there was no difference between regular physiotherapy and regular physiotherapy in conjunction with use of an Oswestry standing frame delivered over 14 consecutive weekdays in the first 3 months post-stroke on trunk postural control.[2] There was low quality evidence that an 8-week course of physiotherapy involving early mobilisation combined with the Bobath approach resulted in improvements in balance when compared to physiotherapy just involving the Bobath approach.[16]

In the acute to late subacute phase, there was very low quality evidence that a 6-week course of robotic tilt-table verticalisation that combines cyclic leg movements and FES and used in conjunction with standard physiotherapy resulted in improved postural control

during different activities compared to physiotherapy-assisted verticalisation using a standard tilt-table and used in conjunction with standard physiotherapy.[18] There was very low-quality evidence that a 3-month course of robot-assisted bodyweight supported treadmill gait training resulted in improvements in trunk control compared to conventional gait training.[25] Improvements were only seen in the cohort of participants who demonstrated significant motor impairment.

In the chronic phase, there was very low-quality evidence that a 6-week course of robot-assisted bodyweight supported treadmill gait training resulted in improvements in balance regardless if slower or faster treadmill training speeds were used.[29] There was very low-quality evidence that a 16-week course of trunk acupuncture combined with rehabilitation training resulted in greater improvements in balance compared to rehabilitation training alone.[32]

Gait

Eight studies investigated gait, which included gait ability and gait speed. Six studies were performed in the acute to early subacute phase,[3-5,8,10,13] one study was performed in the acute to late subacute phase[25] and one study was performed in the chronic phase.[29] In the acute to early subacute phase, there was low quality evidence from two studies that a 6-week course of thermal stimulation in conjunction with standard rehabilitation resulted in improvements in gait ability compared to standard rehabilitation alone.[5,10] There was low quality evidence that 4 weeks of robot-assisted treadmill gait training resulted in better gait ability than conventional gait training.[13] There was low quality evidence that there was no difference between: a 2-week course of robot-assisted bodyweight supported treadmill gait training and conventional gait training delivered in the first 6 weeks post-stroke on improving gait ability;[4] a 4-week course of bodyweight supported treadmill training and conventional overground gait training on improving gait ability;[8] and a thrice weekly, 6-week course of EMG biofeedback combined with conventional physiotherapy and conventional physiotherapy alone in improving gait speed.[3] In the acute to late subacute phase, there was very low-quality evidence that a 3-month course of robot-assisted bodyweight supported treadmill gait training resulted in improvements in gait ability compared to conventional gait training.[25] Improvements were only seen in the cohort of participants who demonstrated significant motor impairment. Improvements were maintained at the 2-year follow-up.[26]

In the chronic phase, there was very low-quality evidence that a 6-week course of robot-assisted bodyweight supported treadmill gait training using slower treadmill speeds resulted in improvements gait ability compared to similar treadmill training using faster treadmill speeds.[29]

General Physical Activity

Seven studies examined the effects of different interventions on improving general physical activity. Six studies were performed in the acute to early subacute phase[2,3,5,10,11,16] and one study was performed in the acute to late subacute phase.[25] In the acute to early subacute phase, there was low quality evidence from two studies that thermal stimulation in conjunction with standard rehabilitation resulted in improvements in physical activity when compared to standard rehabilitation alone.[5,10] Improvements were seen up until 3 months post-intervention but disappeared at the 6-month follow-up. There was low quality evidence that an 8-week course of physiotherapy involving early mobilisation combined with the Bobath approach resulted in improvements in physical activity when compared to

For peer review only

physiotherapy just involving the Bobath approach.[16] There was low quality evidence that there was no difference between: regular physiotherapy and regular physiotherapy in conjunction with use of an Oswestry standing frame delivered over 14 consecutive weekdays in the first 3 months post-stroke on physical activity;[2] and a 5-week course of additional upper limb therapy provided by a qualified physiotherapist or a physiotherapy assistant and standard physiotherapy on improving physical activity.[11] There was very low-quality evidence that there was no difference between a thrice weekly, 6-week course EMG biofeedback combined with conventional physiotherapy and conventional physiotherapy alone on improving physical activity.[3]

In the acute to late subacute phase, there was very low-quality evidence that a 3-month course of robot-assisted bodyweight supported treadmill gait training resulted in improvements in physical activity compared to conventional gait training.[25] Improvements were only seen in the cohort of participants who demonstrated significant motor impairment. Improvements were maintained at the 2-year follow-up.[26]

Upper Limb Function

Two studies investigated changes in upper limb function.[11,31] In the acute to early subacute phase, there was low quality evidence that a 5-week course of additional upper limb therapy provided by a qualified physiotherapist was no more effective at improving upper limb function than additional upper limb therapy provided by a physiotherapy assistant or to standard physiotherapy.[11] In the chronic phase, there was very low-quality evidence that there was no improvement in upper limb function with either an intensive therapist-driven UL protocol or an intensive robotic-driven UL protocol delivered thrice weekly for 6 weeks.[31]

Participation

Instrumental Activities of Daily Living

Four studies investigated the effect of different interventions on instrumental ADLs.[2,3,9,21] In the acute to early subacute phase, there was low quality evidence that there was no difference between: regular physiotherapy and regular physiotherapy in conjunction with use of an Oswestry standing frame delivered over 14 consecutive weekdays on ability to perform instrumental ADLs at 6 months post-stroke,[2] and an 8-week course of rehabilitation with the addition of a leg cycling machine compared to regular rehabilitation alone on instrumental ADLs 6 months post stroke.[9] There was very low-quality evidence that there was no difference between a thrice weekly, 6-week course of electromyography (EMG) biofeedback combined with conventional physiotherapy and conventional physiotherapy alone in improving performance in instrumental ADLs.[3]

In the acute to late subacute phase, there was very low-quality evidence that there was no difference between a functionally orientated or a sensorimotor integrative occupational therapy treatment approach delivered over 8 weeks on the ability to prepare meals.[21]

Perceived Health Status

Two studies explored carers' and patients' perceived health status.[2,31] In the acute to early subacute phase, there was low quality evidence that there was no difference between regular physiotherapy and regular physiotherapy in conjunction with use of an Oswestry standing frame delivered over 14 consecutive weekdays on carer's perceived health status at 12 weeks and 6 months post-stroke.[2] In the chronic phase, there was very low-quality evidence that there was no change in patient's perceived health status with the provision of

For peer review only - http://bmjopen.bmj.com/site/about/guidelines.xhtml

either an intensive therapist-driven UL protocol or an intensive robotic-driven UL protocol delivered thrice weekly for 6 weeks.[31]

Quality of Life
There was very low-quality evidence that a monthly home-based physiotherapy programme delivered over 6 months resulted in an improvement in quality of life compared to standard care.[19]

Complications
Caregiver Burden
There was low quality evidence that there was no difference between regular physiotherapy and regular physiotherapy in conjunction with use of an Oswestry standing frame delivered over 14 consecutive weekdays in the first 3 months post-stroke on caregiver strain and psychological well-being at 12 weeks and 6 months post-stroke.[2]

Depression
Three studies explored changes in depression.[2,20,31] In the acute to early subacute phase, there was low quality evidence that there was no difference between regular physiotherapy and regular physiotherapy in conjunction with use of an Oswestry standing frame delivered over 14 consecutive weekdays on depression at 12 weeks and 6 months post-stroke.[2] In the acute to late subacute phase, there was very low-quality evidence that a monthly home-based physiotherapy programme delivered over 6 months resulted in a reduction in level of depression compared to standard care.[20] In the chronic phase, there was very low-quality evidence that there was no difference between an intensive therapist-driven UL protocol and an intensive robotic-driven UL protocol delivered thrice weekly for 6 weeks in reducing depression.[31]

Shoulder Pain/Dislocation
There was very low-quality evidence that either an intensive therapist-driven UL protocol or an intensive robotic-driven UL protocol delivered thrice weekly for 6 weeks had no effect on shoulder pain nor caused any shoulder dislocation when delivered to participants in the chronic phase post-stroke.[31]

Spasticity
Six studies explored the effect of different interventions on spasticity.[3,8,11,17,25,31] In the acute to early subacute phase, there was low quality evidence that there was no difference between: bodyweight supported treadmill training and conventional overground gait training delivered over 4 weeks on reducing lower limb spasticity;[8] and a 5-week course of additional upper limb therapy provided by a qualified physiotherapist or a physiotherapy assistant and standard physiotherapy on reducing upper limb spasticity.[11] There was very low-quality evidence that there was no reduction in spasticity with a 6-week course of conventional physiotherapy with or without EMG biofeedback.[3]
In the acute to late subacute phase, there was low quality evidence that a 6-month course of a staged physical rehabilitation programme resulted in a lower incidence of upper and lower limb spasticity compared to usual care that did not involve formal rehabilitation.[17]
There was very low-quality evidence that a 3-month course of either robot-assisted

bodyweight supported treadmill training or conventional gait training had no effect on reducing lower limb spasticity.[25]

In the chronic phase, there was very low-quality evidence that there was no difference between an intensive therapist-driven UL protocol and an intensive robotic-driven UL protocol delivered thrice weekly for 6 weeks in reducing UL spasticity.[31]

Supplemental References

1 AVERT Trial Collaboration group. Efficacy and safety of very early mobilisation within 24 h of stroke onset (AVERT): a randomised controlled trial. *Lancet* 2015;386:46-55.

2 Bagley P, Hudson M, Forster A, et al. A randomized trial evaluation of the Oswestry Standing Frame for patients after stroke. *Clin Rehabil* 2005;19:354-364.

3 Bradley L, Hart BB, Mandana S, et al. Electromyographic biofeedback for gait training after stroke. *Clin Rehabil* 1998;12:11-22.

4 Chang WH, Kim MS, Huh JP, et al. Effects of Robot-Assisted Gait Training on Cardiopulmonary Fitness in Subacute Stroke Patients: A Randomized Controlled Study. *Neurorehab Neural Re* 2012;26(4):318-324.

5 Chen JC, Lin CH, Wei YC, et al. Facilitation of motor and balance recovery by thermal intervention for the paretic lower limb of acute stroke: a single-blind randomized clinical trial. *Clin Rehabil* 2011;25(9):823-832.

6 Di Lauro A, Pellegrino L, Savastano G, et al. A randomized trial on the efficacy of intensive rehabilitation in the acute phase of ischemic stroke. *J Neurol* 2003;250:1206-1208.

7 Fong K, Yang N, Chan M, et al. Combined effects of sensory cueing and limb activation on unilateral neglect in subacute left hemiplegic stroke patients: a randomized controlled pilot study. *Clin Rehabil* 2013;27(7):628-637.

8 Franceschini M, Carda S, Agosti M, et al. Walking After Stroke: What Does Treadmill Training With Body Weight Support Add to Overground Gait Training in Patients Early After Stroke? A Single-Blind, Randomized, Controlled Trial. *Stroke* 2009;40:3079-3085.

9 Katz-Leurer M, Carmeli E and Shochina M. The effect of early aerobic training on independence six months post stroke. *Clin Rehabil* 2003;17:735-741.

10 Liang CC, Hsieh TC, Lin CH, et al. Effectiveness of Thermal Stimulation for the Moderately to Severely Paretic Leg After Stroke: Serial Changes at One-Year Follow-Up. *Arch Phys Med Rehab* 2012;93:1903-1910.

11 Lincoln NB, Parry RH and Vass CD. Randomized, Controlled Trial to Evaluate Increased Intensity of Physiotherapy Treatment of Arm Function After Stroke. *Stroke* 1999;30:573-579.

12 Min M, Xin C, Yuefeng C, et al. Stage-oriented Comprehensive Acupuncture Treatment plus Rehabilitation Training for Apoplectic Hemiplegia. *J Tradit Chin Med* 2008;28(2):90-93.

13 Ochi M, Wada F, Saeki S, et al. Gait training in subacute non-ambulatory stroke patients using a full weight-bearing gait-assistance robot: A prospective, randomized, open, blinded-endpoint trial. *J Neurol Sci* 2015;353:130-136.

For peer review only

14 Rosewilliam S, Malhotra S, Roffe C, et al. Can surface neuromuscular electrical stimulation of the wrist and hand combined with routine therapy facilitate recovery of arm function in patients with stroke? *Arch Physl Med Rehab* 2012;93(10):1715-1721.

15 Sanchez-Sanchez ML, Belda-Lois JM, Horno SM, et al. Functional principal component analysis as a new methodology for the analysis of the impact of two rehabilitation protocols in functional recovery after stroke. *J Neuroeng Rehabil* 2014;11:134.

16 Tang Q, Tan L, Li B, et al. Early Sitting, Standing, and Walking in Conjunction With Contemporary Bobath Approach for Stroke Patients With Severe Motor Deficit. *Top Stroke Rehabil* 2014;21(2):120-127.

17 Bai YL, Hu YS, Wu Y, et al. Long-term three-stage rehabilitation intervention alleviates spasticity of the elbows, fingers, and plantar flexors and improves activities of daily living in ischemic stroke patients: a randomized, controlled trial. *Neuroreport* 2014;25:998-1005.

18 Calabro RS, Naro A, Russo M, et al. Do post-stroke patients benefit from robotic verticalization? A pilot-study focusing on a novel neurophysiological approach. *Restor Neurol and Neuros* 2015:33:671-681.

19 Chaiyawat P and Kulkantrakorn K. Effectiveness of home rehabilitation program for ischemic stroke upon disability and quality of life: A randomized controlled trial. *Clin Neurol Neuros* 2012:114:866-870.

20 Chaiyawat P and Kulkantrakorn K. Randomized controlled trial of home rehabilitation for patients with ischemic stroke: impact upon disability and elderly depression. *Psychogeriatrics* 2012;12:193-199.

21 Jongbloed L, Stacey S and Brighton C. Stroke Rehabilitation: Sensorimotor Integrative Treatment Versus Functional Treatment. *Am J Occup Ther* 1989;43(6):391-397.

22 Kwakkel G, Wagenaar RC, Twisk JWR, et al. Intensity of leg and arm training after primary middle-cerebral artery stroke: a randomised trial. *Lancet* 1999;354:191-196.

23 Kwakkel G, Kollen BJ and Wagenaar RC. Long term effects of intensity of upper and lower limb training after stroke: a randomised trial. *J Neurol Neurosur Ps* 2002;72:473-479.

24 Kwakkel G and Wagenaar RC. Effect of Duration of Upper- and Lower-Extremity Rehabilitation Sessions and Walking Speed on Recovery of Interlimb Coordination in Hemiplegic Gait. *Phys Ther* 2002;82(5):432-448.

25 Morone G, Bragoni M, Iosa M, De Angelis D, Venturiero V, Coiro P. Who May Benefit From Robotic- Assisted Gait Training? A Randomized Clinical Trial in Patients With Subacute Stroke. *Neurorehab Neural Re* 2011;25(7):636-644.

26 Morone G, Iosa M, Bragoni M, De Angelis D, Venturiero V, Coiro P. Who May Have Durable Benefit From Robotic Gait Training? A 2-Year Follow-Up Randomized Controlled Trial in Patients With Subacute Stroke. *Stroke* 2012;43:1140-1142.

27 Yang XH, Bing L and Ba-si O. Effect of acupuncture combined with rehabilitative training on neural functional recovery of stroke patients during recovery phase: a randomized controlled trial. *World J Acupunct Mox* 2014;24(4):17-23.

28 Yue S, Jian X and Wong T. Effects of a nurse-led acupressure programme for stroke patients in China. *J Clin Nurs* 2012;22:1182-1188.

For peer review only

29 Rodrigues TA, Goroso DG, Westgate PM, et al. Slow Versus Fast Robot-Assisted Locomotor Training After Severe Stroke. A Randomized Controlled Trial. *Am J Phys Med Rehab* 2017;96(Suppl):S165–S170.

30 Sackley CM, Walker MF, Burton CR, et al. An occupational therapy intervention for residents with stroke related disabilities in UK care homes (OTCH): cluster randomised controlled trial. *BMJ* 2015;350:h468.

31 Volpe BT, Lynch D, Rykman-Berland A, et al. Intensive Sensorimotor Arm Training Mediated by Therapist or Robot Improves Hemiparesis in Patients With Chronic Stroke. *Neurorehabi Neural Re* 2008;22:305-310.

32 Zhang HY and Li PF. Observation on efficacy of acupuncture combined with rehabilitation training for post-stroke balance disorders. *World J Acupunct Mox* 2014;24(2):25-29.

For peer review only
